# JARVIS3: an efficient encoder for genomic data

**DOI:** 10.1093/bioinformatics/btae725

**Published:** 2024-12-02

**Authors:** Maria J P Sousa, Armando J Pinho, Diogo Pratas

**Affiliations:** Institute of Electronics and Informatics Engineering of Aveiro (IEETA), University of Aveiro, Campus Universitário de Santiago, 3810-193 Aveiro, Portugal; Department of Electronics, Telecommunications and Informatics (DETI), University of Aveiro, Campus Universitário de Santiago, 3810-193 Aveiro, Portugal; Intelligent Systems Associate Laboratory (LASI), University of Aveiro, Campus Universitário de Santiago, 3810-193 Aveiro, Portugal; Institute of Electronics and Informatics Engineering of Aveiro (IEETA), University of Aveiro, Campus Universitário de Santiago, 3810-193 Aveiro, Portugal; Department of Electronics, Telecommunications and Informatics (DETI), University of Aveiro, Campus Universitário de Santiago, 3810-193 Aveiro, Portugal; Intelligent Systems Associate Laboratory (LASI), University of Aveiro, Campus Universitário de Santiago, 3810-193 Aveiro, Portugal; Institute of Electronics and Informatics Engineering of Aveiro (IEETA), University of Aveiro, Campus Universitário de Santiago, 3810-193 Aveiro, Portugal; Department of Electronics, Telecommunications and Informatics (DETI), University of Aveiro, Campus Universitário de Santiago, 3810-193 Aveiro, Portugal; Intelligent Systems Associate Laboratory (LASI), University of Aveiro, Campus Universitário de Santiago, 3810-193 Aveiro, Portugal; Department of Virology (DoV), University of Helsinki, Haartmaninkatu 3, 00014 Helsinki, Finland

## Abstract

**Motivation:**

Large-scale genomic projects grapple with the complex challenge of reducing medium- and long-term storage space and its associated energy consumption, monetary costs, and environmental footprint.

**Results:**

We present JARVIS3, an advanced tool engineered for the efficient reference-free compression of genomic sequences. JARVIS3 introduces a pioneering approach, specifically through enhanced table memory models and probabilistic lookup-tables applied in repeat models. These optimizations are pivotal in substantially enhancing computational efficiency. JARVIS3 offers three distinct profiles: (i) rapid computation with moderate compression, (ii) a balanced trade-off between time and compression, and (iii) slower computation with significantly higher compression ratios. The implementation of JARVIS3 is rooted in the C programming language, building upon the success of its predecessor, JARVIS2. JARVIS3 shows substantial speed improvements relative to JARVIS2 while providing slightly better compression. Furthermore, we provide a versatile C/Bash implementation, facilitating the application in FASTA and FASTQ data, including the capability for parallel computation. In addition, JARVIS3 includes a mode for outputting bit information, as well as providing the Normalized Compression and bit rates, facilitating compression-based analysis. This establishes JARVIS3 as an open-source solution for genomic data compression and analysis.

**Availability and implementation:**

JARVIS3 is freely available at https://github.com/cobilab/jarvis3.

## 1 Introduction

Genomic data, found across a vast array of environments, poses distinct challenges for compression and storage. Effectively compressing genomic sequences necessitates the ability to model their heterogeneous, dynamic, and often incomplete nature, while also accounting for specific genomic features such as a high level of substitutions and inverted repeats.

Over the past three decades, extensive research has yielded numerous genomic data compression methods. Evolving from Biocompress in 1993 (Grumbach and Tahi 1993) over forty specialized algorithms emerged. These methods, embrace diverse strategies from simple bit encoding to context modeling and dictionary-based approaches (Hosseini *et al.* 2016, Hernaez *et al.* 2019). A few examples are XM (Cao *et al.* 2007), the GeCo series (Pratas *et al.* 2016, 2020, Silva *et al.* 2020), and the JARVIS series (Pratas *et al.* 2019, Pratas and Pinho 2023).

The widespread availability of complete genomes and their varied representations has prompted the development of the FASTA/FASTQ format, facilitating the integration of genomic sequences alongside annotations and quality scores. Tailored compression algorithms, complemented by other efficient coding techniques, have been devised specifically for these formats. Noteworthy compression tools for FASTA files include MFCompress (Pinho and Pratas 2014), NAF (Kryukov *et al.* 2019), MBGC (Grabowski and Kowalski 2022), and AGC (Deorowicz *et al.* 2023). Similarly, for FASTQ files, compression solutions such as Fqzcomp (Bonfield and Mahoney 2013), DSRC2 (Roguski and Deorowicz 2014), LEON (Benoit *et al.* 2015), and SPRING (Chandak *et al.* 2019) are available.

In addition, reference-free compression techniques are pivotal not only for efficient data storage but also for enabling various analytical applications, including genome reconstruction and classification, rearrangement detection, metagenomic inference, and more. Consequently, advancements in compression tools directly enhance the accuracy of compression-based analysis methods.

In this paper, we introduce JARVIS3, an advanced genomic data compression tool that can accommodate FASTA and FASTQ data. JARVIS3 integrates specialized models, neural networks for context mixing, cache mechanisms to optimize memory utilization and lookup-tables in repeat models to enhance computational efficiency. Our results demonstrate that JARVIS3 achieves competitive or improved compression ratios while significantly reducing computational memory and time demands. These features position JARVIS3 as a valuable tool for current and future challenges in genomic data compression.

## 2 Methodology

### 2.1 Finite-context models

Finite-context models (FCMs) are statistical models assuming the Markov property. A FCM of an information source assigns probability estimates to the symbols of the alphabet, according to a conditioning context computed over a finite and fixed number, *k*, of past outcomes (order-*k* FCM) (see, e.g. Pinho *et al.* 2011). One of the extensions of a FCM is a substitution tolerant context model (STCM) (Pratas *et al.* 2016, 2017). A STCM is a probabilistic-algorithmic context model that acts as a short program enabling to set the number of allowed substitutions in a certain context depth. In practice, it assigns probabilities according to a conditioning context that considers the last symbol, from the sequence to occur, as the most probable, given the occurrences stored in the memory instead of the true occurring symbol.

### 2.2 Repeat models

A repeat model (RM), also known as copy model, predicts that the next outcome will be a symbol that has occurred in the past by maintaining a pointer referring to the symbol being copied, as well as some additional information, needed to estimate how reliable are those predictions.

Let us denote by $x_{1}^{n}=x_{1}x_{2}\ldots x_{n},\;x_{i}\in \Sigma$, the sequence of outputs (symbols from the source alphabet Σ) that the information source has generated until instant *n*. In practical terms, we need to estimate the probability of the next symbol, $x_{n+1}$, being the same as the currently predicted symbol, $x_{n-p}$, where p≥0 is the pointer handled by the copy model.

A repeat model is started after the previous *k*-mer, namely $x_{n-k+1}^{n}$, has been seen somewhere in the past of the sequence. After starting the repeat model, the counts are collected to represent the number of times that each symbol has been correctly predicted (*N_h_* as hits), and the number of times that it was wrongly predicted (*N_m_* as misses), and use these counters for estimating the probability as


(1) $$P(\text{hit})=\frac{N_{h}+1}{N_{h}+N_{m}+2}.$$

The probability of the non hit symbols is uniformly distributed over the complementary probability according to


(2) $$P(s)=\frac{1-P(\text{hit})}{|\Sigma |-1}.$$

Because the repeat model usually does not operate well during a large number of symbols (typically <200), the calculation of (1) and (2) for the possible combinations of *N_h_* and *N_m_* up to $N_{h}+N_{m}\leq 200$ are loaded into two lookup-tables. For values larger than 200 the calculations are analytically performed for each case. This optimization provides substantial increase in the speed, specially when a substantial number of repeat models are used.

The performance of a repeat model along its existence, *Y_i_*, is smoothed using a simple low-pass filter


(3) $$Y_{i}=X_{i}+\beta Y_{i-1},$$

where *X_i_* denotes the instantaneous performance of the model. When *Y_i_* reaches a limit value, *L*, i.e. when $Y_{i}>L$, the repeat model is stopped. Usually, β=0.9 and *L* = 7, but these values depend on the redundancy of the sequence, the number of models being used, and the size of repeating patterns.

To store the positions of occurring *k*-mers along the sequence, various methodologies exist, including cache-tables and hash-tables. We opted for a cache-table implementation, which imposes a maximum of *c* positions while retaining only the most recent *c* entries. To initiate a repeat model with *c* entries, we select a position using pseudo-random selection via a linear congruential generator with a fixed seed. This cache-table approach significantly reduces computation time while maintaining good precision. However, the size of the cache-table is limited to $4^{k}c$. Consequently, for *k* > 15, RAM requirements become prohibitive, making it unfeasible to run without an implementation based, for instance, on a hash-table. Nonetheless, we achieve excellent results with k=12,13,14, even for repetitive and larger sequences, utilizing <4 GB of RAM.

### 2.3 Model mixing

There are two types of model mixing, namely soft-blending and neural network mixing. Although the soft-blending is substantially faster than mixing with a neural network, the latter provides additional compression gains, namely 1%–3%, depending on the parameters. The mixture of models is provided by a soft-blending cooperation with certain forgetting factors. The mixture is derived from Pinho *et al.* (2011) and has a forgetting factor for each model. On the other hand, as in JARVIS2, we use a neural network that is imported from GeCo3 (Silva *et al.* 2020) with minor adaptations. More information about the model mixing is available in Supplementary Section S1, including the soft-blending and neural network approaches.

### 2.4 Pre-set models

JARVIS3 includes 40 preset models. These models are within three main groups, namely

1. Efficient: Modest compression ratios with reduced computational demands; employs either a single repeat model or multiple repeat models with soft-blending cooperation for optimal results.
2. Optimized: Focused on achieving a favorable compression ratio with moderate computational investment; employs a combination of models including FCMs and RMs, with soft-blending cooperation for enhanced performance.
3. Maximal: Dedicated to achieving high to very high compression levels, without using many restrictions on computational time and memory; utilizes a blend of multiple models integrated with neural networks to maximize compression potential.

## 3 Benchmark

To benchmark JARVIS3, we employed ten datasets: the complete Telomere-to-Telomere (T2T) human genome (Nurk *et al.* 2022), the African Cassava TME204 genome (Qi *et al.* 2022), a viral database (Pyöriä *et al.* 2023), a corpus of DNA sequences (Pratas and Pinho 2019), the human reference Y-chromosome (hg38), and the UCSC hg38 7way knownCanonical-exonNuc (Kent *et al.* 2002) (also utilized in Kryukov *et al.* 2020). In addition, the datasets ERR3307082, SRR1284073, SRR8858470, and SRR9046049, retrieved from the SRA and used in Lee and Song (2022), were considered.

Supplementary Sections S2 and S3 offer further insights into the JARVIS3 tool, including its parameters and functionalities, while Supplementary Sections S4 and S5 detail how to reproduce the complete benchmark, including dataset downloads, and the computing characteristics. Supplementary Sections S1.3 and S1.4 provide extended results.

**Figure 1.**
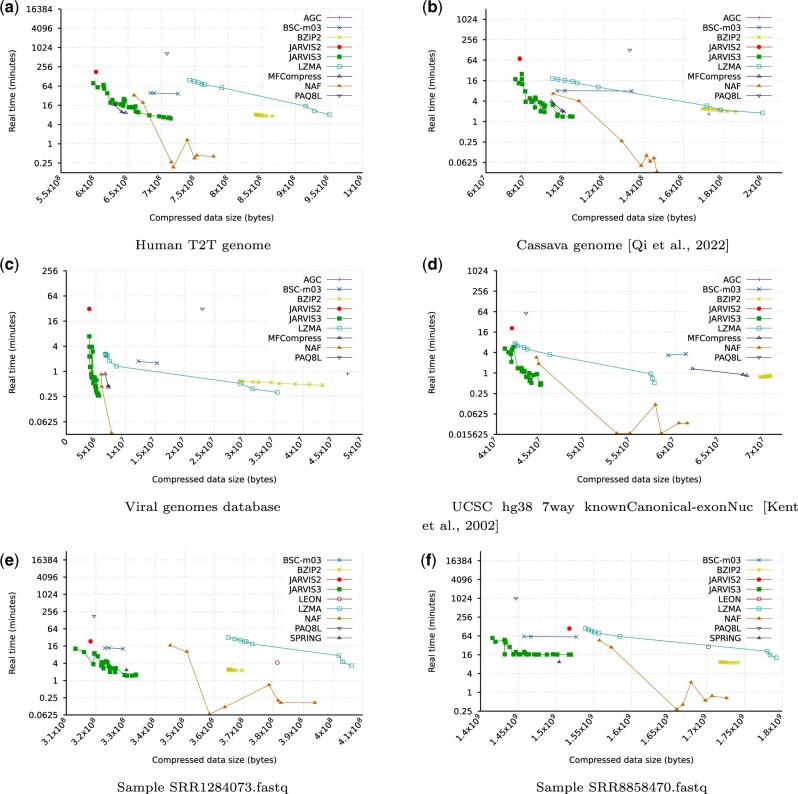
Compression benchmark depicting the compression time and size for 11 compression tools over 6 datasets represented in FASTA and FASTQ formats. The *y*-axis is represented as a logarithmic scale of base 2. The result for the ACG was not represented in (d) as it was an outlier.

Figure 1 illustrates the compression results, demonstrating JARVIS3’s consistent higher compression compared to all other benchmarked data compression tools, achieved with substantially fewer computational resources than its predecessor, JARVIS2. On average, JARVIS3 reduced the compression and decompression time by approximately 84% while improving compression by 1.2% in relation to JARVIS2. In addition to compressors designed for genomic data, we included well-known general-purpose data compression tools such as LZMA, BSC, BZIP2, and PAQ8L for comparison, specifically to assess computational resource usage and to discern the clear advantages of utilizing specialized data compression tools.

In terms of computational memory usage, except for when aiming for maximal compression, JARVIS3 utilizes a maximum of 4 GB of RAM. For faster modes (efficient group), it requires <300 Megabytes.

One drawback of JARVIS3, noted in comparison to tools like AGC and NAF, as well as general-purpose compression tools, is that it requires approximately the same decompression time. In contrast, other tools often achieve decompression with significantly less computational time.

Notably, JARVIS3 has achieved the best compression results to date in the human genome compression benchmark (https://github.com/cobilab/humangenome), compressing approximately 4MB more than the previous best version which was an already very parameter optimized entry. The T2T complete human genome has been compressed to approximately 539 MB.

Lastly, JARVIS3 includes a mode for outputting bit information, as well as providing Normalized Compression and bit rates, facilitating compression-based analysis.

## 4 Conclusions

We introduced JARVIS3, an advanced computational tool designed for compressing genomic data without loss of information. Through benchmarking against other tools, JARVIS3 consistently demonstrated improved compression performance. Building upon the success of its predecessor, JARVIS2, JARVIS3 incorporates enhanced table memory models and probabilistic lookup-tables, representing an evolutionary leap in genomic data compression. Notably, JARVIS3 achieves improved compression results while requiring substantially lower computational resources compared to the best results obtained with JARVIS2. Furthermore, we have made JARVIS3 freely available as an open-source tool, ensuring accessibility and fostering collaboration in the scientific community.

## Supplementary Material

btae725_Supplementary_Data
