# Supplementary material for: JARVIS3: an efficient encoder for genomic data
Source: Bioinformatics. 2024 Dec 2;40(12):btae725. doi: 10.1093/bioinformatics/btae725 (PMC11645547; doi:10.1093/bioinformatics/btae725)
Supplement: btae725_Supplementary_Data [file btae725_supplementary_data.pdf]

Supplementary material of "JARVIS3: an efficient encoder for genomic data"

M. J. Sousa, A. J. Pinho, and D. Pratas

1 Model mixing

There are two types of model mixing, namely soft-blending and neural network mixing. Although the soft-blending is substantially faster than mixing with a neural network, the latter provides additional compression gains, namely 1 to 3%, depending on the parameters and sequences.

1.1 Soft-blending cooperation

The mixture of models is provided by a soft-blending cooperation with a certain global forgetting factor. Specifically, for a certain model, the probability of each symbol, x_i, assuming that the sequence is composed by $x_1, x_2, ..., x_n$, is given by

$$P(x_i) = \sum_{m \in \mathcal{M}} P_m(x_i|x_{i-k}^{i-1})\, w_{m,i}. \tag{1}$$

Notice that $P_m(x_i|x_{i-k}^{i-1})$ is the probability assigned to the next symbol by a model, k is the order of the corresponding model m, and where $w_{m,i}$ denotes the corresponding weighting factor, with

$$w_{m,i} \propto (w_{m,i-1})^{\gamma_m} P_m(x_i|x_{i-k}^{i-1}), \tag{2}$$

where the sum of the weights, for all the respective models, is constrained to one. The $\gamma_m \in [0, 1)$ is derived from [1] and acts as a forgetting factor for each model.

1.2 Artificial Neural Network

We use an ANN that is imported from GeCo3 [2] and JARVIS2 [3] with minor adaptations. Accordingly, we use a multilayer perceptron (MLP), which confers accurate and efficient predictions [4], while being one of the most resource-efficient neural networks regarding time and memory usage. Moreover, it is straightforward to implement and validate, and has proven to provide improved compression ratios both for genomic [2, 3] and proteomic sequences [5, 6].

Specifically, the network has a single hidden layer. The activation function is the sigmoid and the loss function is the mean squared error. The probabilities are stretched according to the works of Matt Mahoney (`http://mattmahoney.net/dc/dce.html`). The inputs to the network are the outputs of the models. One node per symbol is used as output from the network. After the result is transferred to the arithmetic encoder, the network is trained with the current symbol using the stochastic gradient descent algorithm without momentum [7]. This process requires only two parameters, namely the number of nodes of the hidden layer and the learning rate.

1.3 Results of the Benchmark - tabular data

Table S1: Results obtained when compressing the Cassava genome [8].

Compressor	Compressed data size (bytes)	Compression time (minutes)	Compression memory (MB)	Decompression time (minutes)	Decompression memory (MB)	Compression options	Decompression options
AGC	172846185.00	1.67	6668.50	0.03	880.19	./agc create -t 1 CASSAVA.fa	./agc getcol -t 1 AGC-OUT.agc
JARVIS2	77127680.00	71.58	1210.50	70.03	1210.75	./JARVIS2.sh --fasta --level 1 --block 600MB --threads 1 --fasta --input CASSAVA.fa	./JARVIS2.sh --decompress --threads 1 --fasta --input CASSAVA.fa.tar
JARVIS2	77127680.00	70.13	1210.50	70.65	1210.75	./JARVIS2.sh --fasta --level 2 --block 600MB --threads 1 --fasta --input CASSAVA.fa	./JARVIS2.sh --decompress --threads 1 --fasta --input CASSAVA.fa.tar
JARVIS2	77127680.00	69.95	1210.62	70.38	1211.00	./JARVIS2.sh --fasta --level 3 --block 600MB --threads 1 --fasta --input CASSAVA.fa	./JARVIS2.sh --decompress --threads 1 --fasta --input CASSAVA.fa.tar
JARVIS2	77127680.00	70.13	1210.75	70.32	1210.62	./JARVIS2.sh --fasta --level 7 --block 600MB --threads 1 --fasta --input CASSAVA.fa	./JARVIS2.sh --decompress --threads 1 --fasta --input CASSAVA.fa.tar
NAF	146530209.00	0.03	7.00	0.08	6.25	./ennaf --temp-dir tmp/ --dna --level 1 -o NAF-OUT.naf CASSAVA.fa	./unnaf -o NAD-D-OUT.naf NAF-OUT.naf
NAF	138576313.00	0.05	9.00	0.07	6.75	./ennaf --temp-dir tmp/ --dna --level 2 -o NAF-OUT.naf CASSAVA.fa	./unnaf -o NAD-D-OUT.naf NAF-OUT.naf
NAF	143120256.00	0.07	13.50	0.08	7.75	./ennaf --temp-dir tmp/ --dna --level 3 -o NAF-OUT.naf CASSAVA.fa	./unnaf -o NAD-D-OUT.naf NAF-OUT.naf
NAF	145032843.00	0.08	19.62	0.08	7.75	./ennaf --temp-dir tmp/ --dna --level 4 -o NAF-OUT.naf CASSAVA.fa	./unnaf -o NAD-D-OUT.naf NAF-OUT.naf
NAF	141291791.00	0.10	24.88	0.08	7.75	./ennaf --temp-dir tmp/ --dna --level 5 -o NAF-OUT.naf CASSAVA.fa	./unnaf -o NAD-D-OUT.naf NAF-OUT.naf
NAF	128676746.00	0.27	132.50	0.07	9.75	./ennaf --temp-dir tmp/ --dna --level 11 -o NAF-OUT.naf CASSAVA.fa	./unnaf -o NAD-D-OUT.naf NAF-OUT.naf
NAF	107013628.00	4.02	419.25	0.08	13.75	./ennaf --temp-dir tmp/ --dna --level 19 -o NAF-OUT.naf CASSAVA.fa	./unnaf -o NAD-D-OUT.naf NAF-OUT.naf
NAF	93997905.00	6.57	3379.75	0.08	133.75	./ennaf --temp-dir tmp/ --dna --level 22 -o NAF-OUT.naf CASSAVA.fa	./unnaf -o NAD-D-OUT.naf NAF-OUT.naf
LZMA	199973488.00	1.78	9.37	0.35	3.25	./xz -1 -f -k CASSAVA.fa.orig	./xz -f -k -d CASSAVA.fa.orig.xz
LZMA	178669148.00	2.18	15.00	0.30	4.25	./xz -2 -f -k CASSAVA.fa.orig	./xz -f -k -d CASSAVA.fa.orig.xz
LZMA	171851176.00	2.92	26.00	0.28	6.25	./xz -3 -f -k CASSAVA.fa.orig	./xz -f -k -d CASSAVA.fa.orig.xz
LZMA	116840860.00	10.28	42.13	0.18	6.25	./xz -4 -f -k CASSAVA.fa.orig	./xz -f -k -d CASSAVA.fa.orig.xz
LZMA	106151636.00	13.52	80.25	0.17	10.13	./xz -5 -f -k CASSAVA.fa.orig	./xz -f -k -d CASSAVA.fa.orig.xz
LZMA	103919724.00	15.12	80.25	0.17	10.25	./xz -6 -f -k CASSAVA.fa.orig	./xz -f -k -d CASSAVA.fa.orig.xz
LZMA	99989032.00	16.20	156.13	0.17	18.25	./xz -7 -f -k CASSAVA.fa.orig	./xz -f -k -d CASSAVA.fa.orig.xz
LZMA	96495252.00	17.37	308.00	0.17	34.25	./xz -8 -f -k CASSAVA.fa.orig	./xz -f -k -d CASSAVA.fa.orig.xz
LZMA	93717720.00	18.58	612.25	0.17	66.13	./xz -9 -f -k CASSAVA.fa.orig	./xz -f -k -d CASSAVA.fa.orig.xz
BZIP2	186384016.00	1.95	2.38	0.55	1.87	./bzip2 -1 -f -k CASSAVA.fa.orig	./bzip2 -f -k -d CASSAVA.fa.orig.bz2
BZIP2	181932230.00	2.02	3.25	0.57	2.25	./bzip2 -2 -f -k CASSAVA.fa.orig	./bzip2 -f -k -d CASSAVA.fa.orig.bz2
BZIP2	179001656.00	2.10	4.00	0.58	2.63	./bzip2 -3 -f -k CASSAVA.fa.orig	./bzip2 -f -k -d CASSAVA.fa.orig.bz2
BZIP2	176815987.00	2.18	4.75	0.58	3.00	./bzip2 -4 -f -k CASSAVA.fa.orig	./bzip2 -f -k -d CASSAVA.fa.orig.bz2
BZIP2	174866521.00	2.25	5.50	0.58	3.38	./bzip2 -5 -f -k CASSAVA.fa.orig	./bzip2 -f -k -d CASSAVA.fa.orig.bz2
BZIP2	173394999.00	2.27	6.25	0.58	3.75	./bzip2 -6 -f -k CASSAVA.fa.orig	./bzip2 -f -k -d CASSAVA.fa.orig.bz2
BZIP2	172119457.00	2.32	7.13	0.58	4.12	./bzip2 -7 -f -k CASSAVA.fa.orig	./bzip2 -f -k -d CASSAVA.fa.orig.bz2
BZIP2	170794951.00	2.37	7.88	0.58	4.50	./bzip2 -8 -f -k CASSAVA.fa.orig	./bzip2 -f -k -d CASSAVA.fa.orig.bz2
BZIP2	169634896.00	2.38	8.62	0.58	4.87	./bzip2 -9 -f -k CASSAVA.fa.orig	./bzip2 -f -k -d CASSAVA.fa.orig.bz2
BSC-m03	96090883.00	8.02	8847.40	7.13	8211.75	./bsc-m03 e CASSAVA.fa.orig CASSAVA.fa.bsc -b800000000	./bsc-m03 d CASSAVA.fa.bsc CASSAVA.fa.out
BSC-m03	99948804.00	8.08	4734.20	7.12	4407.62	./bsc-m03 e CASSAVA.fa.orig CASSAVA.fa.bsc -b400000000	./bsc-m03 d CASSAVA.fa.bsc CASSAVA.fa.out
BSC-m03	133801203.00	7.88	76.07	7.10	76.70	./bsc-m03 e CASSAVA.fa.orig CASSAVA.fa.bsc -b4096000	./bsc-m03 d CASSAVA.fa.bsc CASSAVA.fa.out
MFCompress	99413533.00	1.92	516.12	1.78	515.25	./MFCompressC -v -1 -p 1 -t 1 -o MFC-OUT.mfc CASSAVA.fa	./MFCompressD -o MFC-OUT.d MFC-OUT.mfc

MFCompress	98578999.00	2.08	516.63	2.23	515.50	./MFCompressC -v -2 -p 1 -t 1 -o MFC-OUT.mfc CASSAVA.fa	./MFCompressD -o MFC-OUT.d MFC-OUT.mfc
MFCompress	93210026.00	3.83	2324.38	2.98	2323.25	./MFCompressC -v -3 -p 1 -t 1 -o MFC-OUT.mfc CASSAVA.fa	./MFCompressD -o MFC-OUT.d MFC-OUT.mfc
PAQ8L	132819459.00	127.58	39.50	127.70	39.37	./paq8l -1 CASSAVA.fa	./paq8l -d CASSAVA.fa.paq8l tmp_dir
JARVIS3	103741440.00	1.40	210.25	1.37	210.00	./JARVIS3.sh --level 1 --block 600MB --threads 1 --fasta --input CASSAVA.fa	./JARVIS3.sh --decompress --threads 1 --fasta --input CASSAVA.fa.tar
JARVIS3	102635520.00	1.42	210.00	1.37	210.00	./JARVIS3.sh --level 2 --block 600MB --threads 1 --fasta --input CASSAVA.fa	./JARVIS3.sh --decompress --threads 1 --fasta --input CASSAVA.fa.tar
JARVIS3	98805760.00	1.40	402.25	1.42	402.12	./JARVIS3.sh --level 3 --block 600MB --threads 1 --fasta --input CASSAVA.fa	./JARVIS3.sh --decompress --threads 1 --fasta --input CASSAVA.fa.tar
JARVIS3	96143360.00	1.45	1170.12	1.48	1169.88	./JARVIS3.sh --level 4 --block 600MB --threads 1 --fasta --input CASSAVA.fa	./JARVIS3.sh --decompress --threads 1 --fasta --input CASSAVA.fa.tar
JARVIS3	96317440.00	1.62	210.25	1.65	210.00	./JARVIS3.sh --level 5 --block 600MB --threads 1 --fasta --input CASSAVA.fa	./JARVIS3.sh --decompress --threads 1 --fasta --input CASSAVA.fa.tar
JARVIS3	89866240.00	2.98	402.37	3.05	401.87	./JARVIS3.sh --level 6 --block 600MB --threads 1 --fasta --input CASSAVA.fa	./JARVIS3.sh --decompress --threads 1 --fasta --input CASSAVA.fa.tar
JARVIS3	89630720.00	1.92	401.87	1.97	402.00	./JARVIS3.sh --level 7 --block 600MB --threads 1 --fasta --input CASSAVA.fa	./JARVIS3.sh --decompress --threads 1 --fasta --input CASSAVA.fa.tar
JARVIS3	87705600.00	2.00	1170.12	2.05	1170.00	./JARVIS3.sh --level 8 --block 600MB --threads 1 --fasta --input CASSAVA.fa	./JARVIS3.sh --decompress --threads 1 --fasta --input CASSAVA.fa.tar
JARVIS3	84879360.00	2.60	1170.25	2.58	1170.00	./JARVIS3.sh --level 9 --block 600MB --threads 1 --fasta --input CASSAVA.fa	./JARVIS3.sh --decompress --threads 1 --fasta --input CASSAVA.fa.tar
JARVIS3	89651200.00	2.67	210.50	2.68	210.00	./JARVIS3.sh --level 10 --block 600MB --threads 1 --fasta --input CASSAVA.fa	./JARVIS3.sh --decompress --threads 1 --fasta --input CASSAVA.fa.tar
JARVIS3	83681280.00	4.23	914.25	4.22	914.00	./JARVIS3.sh --level 11 --block 600MB --threads 1 --fasta --input CASSAVA.fa	./JARVIS3.sh --decompress --threads 1 --fasta --input CASSAVA.fa.tar
JARVIS3	85186560.00	4.58	402.25	4.63	402.00	./JARVIS3.sh --level 12 --block 600MB --threads 1 --fasta --input CASSAVA.fa	./JARVIS3.sh --decompress --threads 1 --fasta --input CASSAVA.fa.tar
JARVIS3	84961280.00	5.07	402.25	5.10	402.00	./JARVIS3.sh --level 13 --block 600MB --threads 1 --fasta --input CASSAVA.fa	./JARVIS3.sh --decompress --threads 1 --fasta --input CASSAVA.fa.tar
JARVIS3	93921280.00	3.10	338.25	3.22	338.25	./JARVIS3.sh --level 14 --block 600MB --threads 1 --fasta --input CASSAVA.fa	./JARVIS3.sh --decompress --threads 1 --fasta --input CASSAVA.fa.tar
JARVIS3	88688640.00	3.35	402.37	3.48	402.12	./JARVIS3.sh --level 15 --block 600MB --threads 1 --fasta --input CASSAVA.fa	./JARVIS3.sh --decompress --threads 1 --fasta --input CASSAVA.fa.tar
JARVIS3	87316480.00	3.67	338.37	3.78	338.12	./JARVIS3.sh --level 16 --block 600MB --threads 1 --fasta --input CASSAVA.fa	./JARVIS3.sh --decompress --threads 1 --fasta --input CASSAVA.fa.tar
JARVIS3	83302400.00	3.80	1170.63	4.00	1170.25	./JARVIS3.sh --level 17 --block 600MB --threads 1 --fasta --input CASSAVA.fa	./JARVIS3.sh --decompress --threads 1 --fasta --input CASSAVA.fa.tar
JARVIS3	83087360.00	3.85	1170.50	3.98	1170.25	./JARVIS3.sh --level 18 --block 600MB --threads 1 --fasta --input CASSAVA.fa	./JARVIS3.sh --decompress --threads 1 --fasta --input CASSAVA.fa.tar
JARVIS3	82196480.00	4.83	402.50	4.95	402.25	./JARVIS3.sh --level 19 --block 600MB --threads 1 --fasta --input CASSAVA.fa	./JARVIS3.sh --decompress --threads 1 --fasta --input CASSAVA.fa.tar
JARVIS3	79892480.00	7.73	466.50	7.85	466.12	./JARVIS3.sh --level 20 --block 600MB --threads 1 --fasta --input CASSAVA.fa	./JARVIS3.sh --decompress --threads 1 --fasta --input CASSAVA.fa.tar
JARVIS3	78428160.00	12.60	530.25	12.75	530.00	./JARVIS3.sh --level 21 --block 600MB --threads 1 --fasta --input CASSAVA.fa	./JARVIS3.sh --decompress --threads 1 --fasta --input CASSAVA.fa.tar
JARVIS3	78264320.00	17.47	530.50	17.68	530.12	./JARVIS3.sh --level 22 --block 600MB --threads 1 --fasta --input CASSAVA.fa	./JARVIS3.sh --decompress --threads 1 --fasta --input CASSAVA.fa.tar
JARVIS3	78182400.00	25.05	594.37	25.17	593.87	./JARVIS3.sh --level 23 --block 600MB --threads 1 --fasta --input CASSAVA.fa	./JARVIS3.sh --decompress --threads 1 --fasta --input CASSAVA.fa.tar
JARVIS3	80097280.00	3.80	4242.50	3.92	4242.12	./JARVIS3.sh --level 24 --block 600MB --threads 1 --fasta --input CASSAVA.fa	./JARVIS3.sh --decompress --threads 1 --fasta --input CASSAVA.fa.tar
JARVIS3	76605440.00	13.40	4242.37	13.65	4242.12	./JARVIS3.sh --level 25 --block 600MB --threads 1 --fasta --input CASSAVA.fa	./JARVIS3.sh --decompress --threads 1 --fasta --input CASSAVA.fa.tar
JARVIS3	74926080.00	17.62	4242.37	17.87	4242.00	./JARVIS3.sh --level 26 --block 600MB --threads 1 --fasta --input CASSAVA.fa	./JARVIS3.sh --decompress --threads 1 --fasta --input CASSAVA.fa.tar

Table S2: Results obtained when compressing the DNA Corpus [9].

Compressor	Compressed data size (bytes)	Compression time (minutes)	Compression memory (MB)	Decompression time (minutes)	Decompression memory (MB)	Compression options	Decompression options
AGC	165969259.00	1.10	6493.10	0.05	2388.49	./agc create -t 1 CORPUS.fa	./agc getcol -t 1 AGC-OUT.agc
JARVIS2	139632640.00	33.90	1255.87	34.08	1256.00	./JARVIS2.sh --fasta --level 1 --block 600MB --threads 1 --fasta --input CORPUS.fa	./JARVIS2.sh --decompress --threads 1 --fasta --input CORPUS.fa.tar
JARVIS2	139632640.00	34.05	1256.00	34.13	1256.12	./JARVIS2.sh --fasta --level 2 --block 600MB --threads 1 --fasta --input CORPUS.fa	./JARVIS2.sh --decompress --threads 1 --fasta --input CORPUS.fa.tar
JARVIS2	139632640.00	33.90	1256.00	34.08	1256.12	./JARVIS2.sh --fasta --level 3 --block 600MB --threads 1 --fasta --input CORPUS.fa	./JARVIS2.sh --decompress --threads 1 --fasta --input CORPUS.fa.tar
JARVIS2	139632640.00	33.83	1256.00	34.10	1256.00	./JARVIS2.sh --fasta --level 7 --block 600MB --threads 1 --fasta --input CORPUS.fa	./JARVIS2.sh --decompress --threads 1 --fasta --input CORPUS.fa.tar
NAF	165348093.00	0.03	6.88	0.07	6.00	./ennaf --temp-dir tmp/ --dna --level 1 -o NAF-OUT.naf CORPUS.fa	./unnaf -o NAD-D-OUT.naf NAF-OUT.naf
NAF	164428531.00	0.05	8.87	0.07	6.50	./ennaf --temp-dir tmp/ --dna --level 2 -o NAF-OUT.naf CORPUS.fa	./unnaf -o NAD-D-OUT.naf NAF-OUT.naf
NAF	172714589.00	0.08	13.50	0.07	7.50	./ennaf --temp-dir tmp/ --dna --level 3 -o NAF-OUT.naf CORPUS.fa	./unnaf -o NAD-D-OUT.naf NAF-OUT.naf
NAF	179350631.00	0.08	19.50	0.05	7.50	./ennaf --temp-dir tmp/ --dna --level 4 -o NAF-OUT.naf CORPUS.fa	./unnaf -o NAD-D-OUT.naf NAF-OUT.naf
NAF	173517621.00	0.10	24.75	0.07	7.50	./ennaf --temp-dir tmp/ --dna --level 5 -o NAF-OUT.naf CORPUS.fa	./unnaf -o NAD-D-OUT.naf NAF-OUT.naf
NAF	170485232.00	0.30	132.25	0.07	9.50	./ennaf --temp-dir tmp/ --dna --level 11 -o NAF-OUT.naf CORPUS.fa	./unnaf -o NAD-D-OUT.naf NAF-OUT.naf
NAF	154874978.00	4.47	418.75	0.07	13.50	./ennaf --temp-dir tmp/ --dna --level 19 -o NAF-OUT.naf CORPUS.fa	./unnaf -o NAD-D-OUT.naf NAF-OUT.naf
NAF	153268866.00	7.38	3379.25	0.07	133.50	./ennaf --temp-dir tmp/ --dna --level 22 -o NAF-OUT.naf CORPUS.fa	./unnaf -o NAD-D-OUT.naf NAF-OUT.naf
LZMA	208588892.00	1.82	8.87	0.35	3.25	./xz -1 -f -k CORPUS.fa.orig	./xz -f -k -d CORPUS.fa.orig.xz
LZMA	204301840.00	2.40	14.38	0.33	4.25	./xz -2 -f -k CORPUS.fa.orig	./xz -f -k -d CORPUS.fa.orig.xz
LZMA	201853460.00	3.33	25.25	0.32	6.25	./xz -3 -f -k CORPUS.fa.orig	./xz -f -k -d CORPUS.fa.orig.xz
LZMA	174484864.00	13.80	41.38	0.23	6.25	./xz -4 -f -k CORPUS.fa.orig	./xz -f -k -d CORPUS.fa.orig.xz
LZMA	169117396.00	17.20	79.37	0.23	10.25	./xz -5 -f -k CORPUS.fa.orig	./xz -f -k -d CORPUS.fa.orig.xz
LZMA	168628400.00	17.68	79.50	0.23	10.25	./xz -6 -f -k CORPUS.fa.orig	./xz -f -k -d CORPUS.fa.orig.xz
LZMA	167243016.00	19.43	155.50	0.23	18.25	./xz -7 -f -k CORPUS.fa.orig	./xz -f -k -d CORPUS.fa.orig.xz
LZMA	166098560.00	21.28	307.50	0.25	34.25	./xz -8 -f -k CORPUS.fa.orig	./xz -f -k -d CORPUS.fa.orig.xz
LZMA	165322512.00	23.05	611.50	0.25	66.25	./xz -9 -f -k CORPUS.fa.orig	./xz -f -k -d CORPUS.fa.orig.xz
BZIP2	192892687.00	1.62	2.38	0.55	1.87	./bzip2 -1 -f -k CORPUS.fa.orig	./bzip2 -f -k -d CORPUS.fa.orig.bz2
BZIP2	192040571.00	1.63	3.13	0.57	2.25	./bzip2 -2 -f -k CORPUS.fa.orig	./bzip2 -f -k -d CORPUS.fa.orig.bz2
BZIP2	191549090.00	1.67	3.75	0.57	2.50	./bzip2 -3 -f -k CORPUS.fa.orig	./bzip2 -f -k -d CORPUS.fa.orig.bz2
BZIP2	191197976.00	1.68	4.37	0.57	3.00	./bzip2 -4 -f -k CORPUS.fa.orig	./bzip2 -f -k -d CORPUS.fa.orig.bz2
BZIP2	190917751.00	1.72	5.12	0.58	3.38	./bzip2 -5 -f -k CORPUS.fa.orig	./bzip2 -f -k -d CORPUS.fa.orig.bz2
BZIP2	190626383.00	1.73	5.75	0.58	3.63	./bzip2 -6 -f -k CORPUS.fa.orig	./bzip2 -f -k -d CORPUS.fa.orig.bz2
BZIP2	190422224.00	1.75	6.38	0.58	4.12	./bzip2 -7 -f -k CORPUS.fa.orig	./bzip2 -f -k -d CORPUS.fa.orig.bz2
BZIP2	190210464.00	1.77	7.13	0.58	4.50	./bzip2 -8 -f -k CORPUS.fa.orig	./bzip2 -f -k -d CORPUS.fa.orig.bz2
BZIP2	190057196.00	1.77	7.75	0.58	4.87	./bzip2 -9 -f -k CORPUS.fa.orig	./bzip2 -f -k -d CORPUS.fa.orig.bz2
BSC-m03	163704583.00	8.57	9030.25	7.82	8525.63	./bsc-m03 e CORPUS.fa.orig CORPUS.fa.bsc -b800000000	./bsc-m03 d CORPUS.fa.bsc CORPUS.fa.out
BSC-m03	163323179.00	8.55	5227.06	7.78	4935.87	./bsc-m03 e CORPUS.fa.orig CORPUS.fa.bsc -b400000000	./bsc-m03 d CORPUS.fa.bsc CORPUS.fa.out
BSC-m03	167731815.00	8.10	78.30	7.47	78.88	./bsc-m03 e CORPUS.fa.orig CORPUS.fa.bsc -b4096000	./bsc-m03 d CORPUS.fa.bsc CORPUS.fa.out
MFCompress	154333166.00	1.92	515.25	1.55	514.25	./MFCompressC -v -1 -p 1 -t 1 -o MFC-OUT.mfc CORPUS.fa	./MFCompressD -o MFC-OUT.d MFC-OUT.mfc

MFCompress	152974757.00	2.10	515.75	1.73	514.62	./MFCompressC -v -2 -p 1 -t 1 -o MFC-OUT.mfc CORPUS.fa	./MFCompressD -o MFC-OUT.d MFC-OUT.mfc
MFCompress	150993377.00	3.75	2323.37	2.35	2322.25	./MFCompressC -v -3 -p 1 -t 1 -o MFC-OUT.mfc CORPUS.fa	./MFCompressD -o MFC-OUT.d MFC-OUT.mfc
PAQ8L	164362931.00	116.63	39.50	116.62	39.37	./paq8l -1 CORPUS.fa	./paq8l -d CORPUS.fa.paq8l tmp_dir
JARVIS3	162375680.00	1.47	210.25	1.47	210.12	./JARVIS3.sh --level 1 --block 600MB --threads 1 --fasta --input CORPUS.fa	./JARVIS3.sh --decompress --threads 1 --fasta --input CORPUS.fa.tar
JARVIS3	162027520.00	1.47	210.25	1.47	209.87	./JARVIS3.sh --level 2 --block 600MB --threads 1 --fasta --input CORPUS.fa	./JARVIS3.sh --decompress --threads 1 --fasta --input CORPUS.fa.tar
JARVIS3	160675840.00	1.52	402.25	1.55	402.00	./JARVIS3.sh --level 3 --block 600MB --threads 1 --fasta --input CORPUS.fa	./JARVIS3.sh --decompress --threads 1 --fasta --input CORPUS.fa.tar
JARVIS3	159477760.00	1.63	1170.37	1.62	1170.00	./JARVIS3.sh --level 4 --block 600MB --threads 1 --fasta --input CORPUS.fa	./JARVIS3.sh --decompress --threads 1 --fasta --input CORPUS.fa.tar
JARVIS3	156190720.00	1.77	210.25	1.80	209.87	./JARVIS3.sh --level 5 --block 600MB --threads 1 --fasta --input CORPUS.fa	./JARVIS3.sh --decompress --threads 1 --fasta --input CORPUS.fa.tar
JARVIS3	152166400.00	3.37	402.25	3.38	401.87	./JARVIS3.sh --level 6 --block 600MB --threads 1 --fasta --input CORPUS.fa	./JARVIS3.sh --decompress --threads 1 --fasta --input CORPUS.fa.tar
JARVIS3	153077760.00	2.22	402.25	2.23	402.00	./JARVIS3.sh --level 7 --block 600MB --threads 1 --fasta --input CORPUS.fa	./JARVIS3.sh --decompress --threads 1 --fasta --input CORPUS.fa.tar
JARVIS3	152688640.00	2.35	1170.25	2.37	1170.00	./JARVIS3.sh --level 8 --block 600MB --threads 1 --fasta --input CORPUS.fa	./JARVIS3.sh --decompress --threads 1 --fasta --input CORPUS.fa.tar
JARVIS3	150681600.00	3.15	1170.25	3.18	1170.00	./JARVIS3.sh --level 9 --block 600MB --threads 1 --fasta --input CORPUS.fa	./JARVIS3.sh --decompress --threads 1 --fasta --input CORPUS.fa.tar
JARVIS3	150906880.00	3.12	210.25	3.10	209.87	./JARVIS3.sh --level 10 --block 600MB --threads 1 --fasta --input CORPUS.fa	./JARVIS3.sh --decompress --threads 1 --fasta --input CORPUS.fa.tar
JARVIS3	148899840.00	4.82	914.25	4.82	913.87	./JARVIS3.sh --level 11 --block 600MB --threads 1 --fasta --input CORPUS.fa	./JARVIS3.sh --decompress --threads 1 --fasta --input CORPUS.fa.tar
JARVIS3	148633600.00	5.32	402.12	5.32	402.00	./JARVIS3.sh --level 12 --block 600MB --threads 1 --fasta --input CORPUS.fa	./JARVIS3.sh --decompress --threads 1 --fasta --input CORPUS.fa.tar
JARVIS3	148244480.00	5.83	402.12	5.82	402.00	./JARVIS3.sh --level 13 --block 600MB --threads 1 --fasta --input CORPUS.fa	./JARVIS3.sh --decompress --threads 1 --fasta --input CORPUS.fa.tar
JARVIS3	149647360.00	3.25	338.25	3.33	338.12	./JARVIS3.sh --level 14 --block 600MB --threads 1 --fasta --input CORPUS.fa	./JARVIS3.sh --decompress --threads 1 --fasta --input CORPUS.fa.tar
JARVIS3	147261440.00	3.53	402.37	3.62	402.12	./JARVIS3.sh --level 15 --block 600MB --threads 1 --fasta --input CORPUS.fa	./JARVIS3.sh --decompress --threads 1 --fasta --input CORPUS.fa.tar
JARVIS3	146370560.00	3.92	338.50	4.00	338.25	./JARVIS3.sh --level 16 --block 600MB --threads 1 --fasta --input CORPUS.fa	./JARVIS3.sh --decompress --threads 1 --fasta --input CORPUS.fa.tar
JARVIS3	145418240.00	4.13	1170.37	4.27	1170.12	./JARVIS3.sh --level 17 --block 600MB --threads 1 --fasta --input CORPUS.fa	./JARVIS3.sh --decompress --threads 1 --fasta --input CORPUS.fa.tar
JARVIS3	145408000.00	4.13	1170.50	4.22	1170.25	./JARVIS3.sh --level 18 --block 600MB --threads 1 --fasta --input CORPUS.fa	./JARVIS3.sh --decompress --threads 1 --fasta --input CORPUS.fa.tar
JARVIS3	144097280.00	5.43	402.50	5.47	402.25	./JARVIS3.sh --level 19 --block 600MB --threads 1 --fasta --input CORPUS.fa	./JARVIS3.sh --decompress --threads 1 --fasta --input CORPUS.fa.tar
JARVIS3	142653440.00	8.68	466.50	8.73	466.12	./JARVIS3.sh --level 20 --block 600MB --threads 1 --fasta --input CORPUS.fa	./JARVIS3.sh --decompress --threads 1 --fasta --input CORPUS.fa.tar
JARVIS3	141967360.00	11.52	530.37	11.60	530.37	./JARVIS3.sh --level 21 --block 600MB --threads 1 --fasta --input CORPUS.fa	./JARVIS3.sh --decompress --threads 1 --fasta --input CORPUS.fa.tar
JARVIS3	141916160.00	12.37	530.37	12.48	530.25	./JARVIS3.sh --level 22 --block 600MB --threads 1 --fasta --input CORPUS.fa	./JARVIS3.sh --decompress --threads 1 --fasta --input CORPUS.fa.tar
JARVIS3	141957120.00	13.22	594.00	13.37	594.00	./JARVIS3.sh --level 23 --block 600MB --threads 1 --fasta --input CORPUS.fa	./JARVIS3.sh --decompress --threads 1 --fasta --input CORPUS.fa.tar
JARVIS3	144117760.00	4.43	4242.37	4.43	4242.12	./JARVIS3.sh --level 24 --block 600MB --threads 1 --fasta --input CORPUS.fa	./JARVIS3.sh --decompress --threads 1 --fasta --input CORPUS.fa.tar
JARVIS3	140738560.00	12.98	4242.37	13.18	4242.25	./JARVIS3.sh --level 25 --block 600MB --threads 1 --fasta --input CORPUS.fa	./JARVIS3.sh --decompress --threads 1 --fasta --input CORPUS.fa.tar
JARVIS3	139530240.00	17.25	4242.63	17.40	4242.00	./JARVIS3.sh --level 26 --block 600MB --threads 1 --fasta --input CORPUS.fa	./JARVIS3.sh --decompress --threads 1 --fasta --input CORPUS.fa.tar

Table S3: Results obtained when compressing the Human Y chromosome.

Compressor	Compressed data size (bytes)	Compression time (minutes)	Compression memory (MB)	Decompression time (minutes)	Decompression memory (MB)	Compression options	Decompression options
AGC	5368114.00	0.08	681.96	0.00	215.32	./agc create -t 1 CY.fa	./agc getcol -t 1 AGC-OUT.agc
JARVIS2	3164160.00	1.72	596.88	1.75	596.75	./JARVIS2.sh --fasta --level 1 --block 600MB --threads 1 --fasta --input CY.fa	./JARVIS2.sh --decompress --threads 1 --fasta --input CY.fa.tar
JARVIS2	3164160.00	1.68	596.75	1.70	596.88	./JARVIS2.sh --fasta --level 2 --block 600MB --threads 1 --fasta --input CY.fa	./JARVIS2.sh --decompress --threads 1 --fasta --input CY.fa.tar
JARVIS2	3164160.00	1.70	596.88	1.72	596.75	./JARVIS2.sh --fasta --level 3 --block 600MB --threads 1 --fasta --input CY.fa	./JARVIS2.sh --decompress --threads 1 --fasta --input CY.fa.tar
JARVIS2	3164160.00	1.72	596.75	1.70	596.75	./JARVIS2.sh --fasta --level 7 --block 600MB --threads 1 --fasta --input CY.fa	./JARVIS2.sh --decompress --threads 1 --fasta --input CY.fa.tar
NAF	5201495.00	0.00	6.50	0.00	3.63	./ennaf --temp-dir tmp/ --dna --level 1 -o NAF-OUT.naf CY.fa	./unnaf -o NAD-D-OUT.naf NAF-OUT.naf
NAF	5072435.00	0.00	8.00	0.00	4.12	./ennaf --temp-dir tmp/ --dna --level 2 -o NAF-OUT.naf CY.fa	./unnaf -o NAD-D-OUT.naf NAF-OUT.naf
NAF	5274393.00	0.00	11.38	0.00	5.12	./ennaf --temp-dir tmp/ --dna --level 3 -o NAF-OUT.naf CY.fa	./unnaf -o NAD-D-OUT.naf NAF-OUT.naf
NAF	5425443.00	0.00	17.75	0.00	5.12	./ennaf --temp-dir tmp/ --dna --level 4 -o NAF-OUT.naf CY.fa	./unnaf -o NAD-D-OUT.naf NAF-OUT.naf
NAF	5275134.00	0.00	22.88	0.00	5.12	./ennaf --temp-dir tmp/ --dna --level 5 -o NAF-OUT.naf CY.fa	./unnaf -o NAD-D-OUT.naf NAF-OUT.naf
NAF	5084159.00	0.00	130.00	0.00	7.13	./ennaf --temp-dir tmp/ --dna --level 11 -o NAF-OUT.naf CY.fa	./unnaf -o NAD-D-OUT.naf NAF-OUT.naf
NAF	4516880.00	0.12	416.50	0.00	11.13	./ennaf --temp-dir tmp/ --dna --level 19 -o NAF-OUT.naf CY.fa	./unnaf -o NAD-D-OUT.naf NAF-OUT.naf
NAF	4499861.00	0.15	3277.00	0.00	31.12	./ennaf --temp-dir tmp/ --dna --level 22 -o NAF-OUT.naf CY.fa	./unnaf -o NAD-D-OUT.naf NAF-OUT.naf
LZMA	6829712.00	0.07	9.25	0.00	3.25	./xz -1 -f -k CY.fa.orig	./xz -f -k -d CY.fa.orig.xz
LZMA	6586156.00	0.08	14.75	0.00	4.25	./xz -2 -f -k CY.fa.orig	./xz -f -k -d CY.fa.orig.xz
LZMA	6477388.00	0.12	25.62	0.00	6.25	./xz -3 -f -k CY.fa.orig	./xz -f -k -d CY.fa.orig.xz
LZMA	5123104.00	0.45	41.88	0.00	6.25	./xz -4 -f -k CY.fa.orig	./xz -f -k -d CY.fa.orig.xz
LZMA	4875584.00	0.60	79.87	0.00	10.25	./xz -5 -f -k CY.fa.orig	./xz -f -k -d CY.fa.orig.xz
LZMA	4854748.00	0.83	79.87	0.00	10.25	./xz -6 -f -k CY.fa.orig	./xz -f -k -d CY.fa.orig.xz
LZMA	4777436.00	0.87	155.88	0.00	18.25	./xz -7 -f -k CY.fa.orig	./xz -f -k -d CY.fa.orig.xz
LZMA	4757876.00	0.90	307.87	0.00	34.25	./xz -8 -f -k CY.fa.orig	./xz -f -k -d CY.fa.orig.xz
LZMA	4755752.00	0.88	515.87	0.00	59.13	./xz -9 -f -k CY.fa.orig	./xz -f -k -d CY.fa.orig.xz
BZIP2	6331350.00	0.08	2.50	0.02	1.87	./bzip2 -1 -f -k CY.fa.orig	./bzip2 -f -k -d CY.fa.orig.bz2
BZIP2	6288602.00	0.08	3.25	0.02	2.25	./bzip2 -2 -f -k CY.fa.orig	./bzip2 -f -k -d CY.fa.orig.bz2
BZIP2	6250770.00	0.08	4.00	0.02	2.63	./bzip2 -3 -f -k CY.fa.orig	./bzip2 -f -k -d CY.fa.orig.bz2
BZIP2	6239130.00	0.10	4.75	0.02	3.00	./bzip2 -4 -f -k CY.fa.orig	./bzip2 -f -k -d CY.fa.orig.bz2
BZIP2	6226969.00	0.10	5.50	0.02	3.38	./bzip2 -5 -f -k CY.fa.orig	./bzip2 -f -k -d CY.fa.orig.bz2
BZIP2	6203567.00	0.10	6.38	0.02	3.75	./bzip2 -6 -f -k CY.fa.orig	./bzip2 -f -k -d CY.fa.orig.bz2
BZIP2	6206171.00	0.10	7.13	0.02	4.12	./bzip2 -7 -f -k CY.fa.orig	./bzip2 -f -k -d CY.fa.orig.bz2
BZIP2	6186603.00	0.12	7.88	0.02	4.50	./bzip2 -8 -f -k CY.fa.orig	./bzip2 -f -k -d CY.fa.orig.bz2
BZIP2	6170382.00	0.12	8.62	0.02	4.87	./bzip2 -9 -f -k CY.fa.orig	./bzip2 -f -k -d CY.fa.orig.bz2
BSC-m03	5150684.00	0.40	684.81	0.35	632.63	./bsc-m03 e CY.fa.orig CY.fa.bsc -b800000000	./bsc-m03 d CY.fa.bsc CY.fa.out
BSC-m03	5150684.00	0.40	684.94	0.35	632.63	./bsc-m03 e CY.fa.orig CY.fa.bsc -b400000000	./bsc-m03 d CY.fa.bsc CY.fa.out
BSC-m03	5353337.00	0.38	75.88	0.33	76.32	./bsc-m03 e CY.fa.orig CY.fa.bsc -b4096000	./bsc-m03 d CY.fa.bsc CY.fa.out
MFCompress	4110220.00	0.10	514.00	0.10	513.13	./MFCompressC -v -1 -p 1 -t 1 -o MFC-OUT.mfc CY.fa	./MFCompressD -o MFC-OUT.d MFC-OUT.mfc
MFCompress	4046719.00	0.12	514.50	0.12	513.50	./MFCompressC -v -2 -p 1 -t 1 -o MFC-OUT.mfc CY.fa	./MFCompressD -o MFC-OUT.d MFC-OUT.mfc

MFCompress	3975085.00	0.22	2259.50	0.18	2258.50	./MFCompressC -v -3 -p 1 -t 1 -o MFC-OUT.mfc CY.fa	./MFCompressD -o MFC-OUT.d MFC-OUT.mfc
PAQ8L	5219521.00	9.58	39.50	9.62	39.50	./paq8l -1 CY.fa	./paq8l -d CY.fa.paq8l tmp_dir
JARVIS3	4423680.00	0.07	72.75	0.05	72.37	./JARVIS3.sh --level 1 --block 600MB --threads 1 --fasta --input CY.fa	./JARVIS3.sh --decompress --threads 1 --fasta --input CY.fa.tar
JARVIS3	3880960.00	0.07	72.75	0.05	72.37	./JARVIS3.sh --level 2 --block 600MB --threads 1 --fasta --input CY.fa	./JARVIS3.sh --decompress --threads 1 --fasta --input CY.fa.tar
JARVIS3	3788800.00	0.07	264.25	0.05	264.13	./JARVIS3.sh --level 3 --block 600MB --threads 1 --fasta --input CY.fa	./JARVIS3.sh --decompress --threads 1 --fasta --input CY.fa.tar
JARVIS3	3737600.00	0.07	1008.00	0.07	1007.88	./JARVIS3.sh --level 4 --block 600MB --threads 1 --fasta --input CY.fa	./JARVIS3.sh --decompress --threads 1 --fasta --input CY.fa.tar
JARVIS3	3676160.00	0.07	72.50	0.05	72.37	./JARVIS3.sh --level 5 --block 600MB --threads 1 --fasta --input CY.fa	./JARVIS3.sh --decompress --threads 1 --fasta --input CY.fa.tar
JARVIS3	3532800.00	0.10	264.38	0.10	264.13	./JARVIS3.sh --level 6 --block 600MB --threads 1 --fasta --input CY.fa	./JARVIS3.sh --decompress --threads 1 --fasta --input CY.fa.tar
JARVIS3	3553280.00	0.07	264.25	0.08	264.00	./JARVIS3.sh --level 7 --block 600MB --threads 1 --fasta --input CY.fa	./JARVIS3.sh --decompress --threads 1 --fasta --input CY.fa.tar
JARVIS3	3532800.00	0.08	1008.25	0.08	1007.75	./JARVIS3.sh --level 8 --block 600MB --threads 1 --fasta --input CY.fa	./JARVIS3.sh --decompress --threads 1 --fasta --input CY.fa.tar
JARVIS3	3481600.00	0.10	1008.12	0.10	1007.75	./JARVIS3.sh --level 9 --block 600MB --threads 1 --fasta --input CY.fa	./JARVIS3.sh --decompress --threads 1 --fasta --input CY.fa.tar
JARVIS3	3491840.00	0.10	72.63	0.10	72.37	./JARVIS3.sh --level 10 --block 600MB --threads 1 --fasta --input CY.fa	./JARVIS3.sh --decompress --threads 1 --fasta --input CY.fa.tar
JARVIS3	3420160.00	0.13	766.75	0.15	766.50	./JARVIS3.sh --level 11 --block 600MB --threads 1 --fasta --input CY.fa	./JARVIS3.sh --decompress --threads 1 --fasta --input CY.fa.tar
JARVIS3	3409920.00	0.15	264.50	0.15	264.00	./JARVIS3.sh --level 12 --block 600MB --threads 1 --fasta --input CY.fa	./JARVIS3.sh --decompress --threads 1 --fasta --input CY.fa.tar
JARVIS3	3399680.00	0.17	264.50	0.15	264.00	./JARVIS3.sh --level 13 --block 600MB --threads 1 --fasta --input CY.fa	./JARVIS3.sh --decompress --threads 1 --fasta --input CY.fa.tar
JARVIS3	3573760.00	0.12	200.75	0.10	200.63	./JARVIS3.sh --level 14 --block 600MB --threads 1 --fasta --input CY.fa	./JARVIS3.sh --decompress --threads 1 --fasta --input CY.fa.tar
JARVIS3	3450880.00	0.12	264.38	0.12	264.25	./JARVIS3.sh --level 15 --block 600MB --threads 1 --fasta --input CY.fa	./JARVIS3.sh --decompress --threads 1 --fasta --input CY.fa.tar
JARVIS3	3409920.00	0.13	200.88	0.13	200.63	./JARVIS3.sh --level 16 --block 600MB --threads 1 --fasta --input CY.fa	./JARVIS3.sh --decompress --threads 1 --fasta --input CY.fa.tar
JARVIS3	3358720.00	0.13	1008.25	0.13	1008.12	./JARVIS3.sh --level 17 --block 600MB --threads 1 --fasta --input CY.fa	./JARVIS3.sh --decompress --threads 1 --fasta --input CY.fa.tar
JARVIS3	3358720.00	0.15	1008.37	0.13	1008.00	./JARVIS3.sh --level 18 --block 600MB --threads 1 --fasta --input CY.fa	./JARVIS3.sh --decompress --threads 1 --fasta --input CY.fa.tar
JARVIS3	3307520.00	0.15	264.50	0.17	264.25	./JARVIS3.sh --level 19 --block 600MB --threads 1 --fasta --input CY.fa	./JARVIS3.sh --decompress --threads 1 --fasta --input CY.fa.tar
JARVIS3	3256320.00	0.22	328.00	0.23	327.87	./JARVIS3.sh --level 20 --block 600MB --threads 1 --fasta --input CY.fa	./JARVIS3.sh --decompress --threads 1 --fasta --input CY.fa.tar
JARVIS3	3235840.00	0.30	391.37	0.30	391.25	./JARVIS3.sh --level 21 --block 600MB --threads 1 --fasta --input CY.fa	./JARVIS3.sh --decompress --threads 1 --fasta --input CY.fa.tar
JARVIS3	3235840.00	0.38	391.62	0.38	391.37	./JARVIS3.sh --level 22 --block 600MB --threads 1 --fasta --input CY.fa	./JARVIS3.sh --decompress --threads 1 --fasta --input CY.fa.tar
JARVIS3	3235840.00	0.50	454.88	0.50	454.75	./JARVIS3.sh --level 23 --block 600MB --threads 1 --fasta --input CY.fa	./JARVIS3.sh --decompress --threads 1 --fasta --input CY.fa.tar
JARVIS3	3368960.00	0.17	3595.25	0.17	3595.12	./JARVIS3.sh --level 24 --block 600MB --threads 1 --fasta --input CY.fa	./JARVIS3.sh --decompress --threads 1 --fasta --input CY.fa.tar
JARVIS3	3256320.00	0.45	3595.38	0.47	3595.12	./JARVIS3.sh --level 25 --block 600MB --threads 1 --fasta --input CY.fa	./JARVIS3.sh --decompress --threads 1 --fasta --input CY.fa.tar
JARVIS3	3205120.00	0.55	3595.25	0.57	3595.25	./JARVIS3.sh --level 26 --block 600MB --threads 1 --fasta --input CY.fa	./JARVIS3.sh --decompress --threads 1 --fasta --input CY.fa.tar

Table S4: Results obtained when compressing the ERR3307082.

Compressor	Compressed data size (bytes)	Compression time (minutes)	Compression memory (MB)	Decompression time (minutes)	Decompression memory (MB)	Compression options	Decompression options
JARVIS2	1335009280.00	81.05	1250.88	82.18	1250.88	./JARVIS2.sh --fastq --level 1 --block 600MB --threads 1 --fastq --input ERR3307082.fastq	./JARVIS2.sh --decompress --threads 1 --fastq --input ERR3307082.fastq.tar
JARVIS2	1335009280.00	81.38	1251.00	81.48	1250.88	./JARVIS2.sh --fastq --level 2 --block 600MB --threads 1 --fastq --input ERR3307082.fastq	./JARVIS2.sh --decompress --threads 1 --fastq --input ERR3307082.fastq.tar
JARVIS2	1335009280.00	80.90	1250.88	81.53	1251.12	./JARVIS2.sh --fastq --level 3 --block 600MB --threads 1 --fastq --input ERR3307082.fastq	./JARVIS2.sh --decompress --threads 1 --fastq --input ERR3307082.fastq.tar
JARVIS2	1335009280.00	81.67	1251.00	81.67	1250.88	./JARVIS2.sh --fastq --level 7 --block 600MB --threads 1 --fastq --input ERR3307082.fastq	./JARVIS2.sh --decompress --threads 1 --fastq --input ERR3307082.fastq.tar
NAF	1510778554.00	0.18	18.13	0.53	472.99	./ennaf --temp-dir tmp/ --fastq --level 1 -o NAF-OUT.naf ERR3307082.fastq	./unnaf -o NAD-D-OUT.naf NAF-OUT.naf
NAF	1508702003.00	0.35	22.50	0.60	473.92	./ennaf --temp-dir tmp/ --fastq --level 2 -o NAF-OUT.naf ERR3307082.fastq	./unnaf -o NAD-D-OUT.naf NAF-OUT.naf
NAF	1536386845.00	0.62	30.88	0.53	492.45	./ennaf --temp-dir tmp/ --fastq --level 3 -o NAF-OUT.naf ERR3307082.fastq	./unnaf -o NAD-D-OUT.naf NAF-OUT.naf
NAF	1570781084.00	0.67	38.62	0.55	512.03	./ennaf --temp-dir tmp/ --fastq --level 4 -o NAF-OUT.naf ERR3307082.fastq	./unnaf -o NAD-D-OUT.naf NAF-OUT.naf
NAF	1533151274.00	0.73	44.87	0.55	497.08	./ennaf --temp-dir tmp/ --fastq --level 5 -o NAF-OUT.naf ERR3307082.fastq	./unnaf -o NAD-D-OUT.naf NAF-OUT.naf
NAF	1531836509.00	2.18	180.63	0.62	494.77	./ennaf --temp-dir tmp/ --fastq --level 11 -o NAF-OUT.naf ERR3307082.fastq	./unnaf -o NAD-D-OUT.naf NAF-OUT.naf
NAF	1472167335.00	32.32	538.50	0.65	471.64	./ennaf --temp-dir tmp/ --fastq --level 19 -o NAF-OUT.naf ERR3307082.fastq	./unnaf -o NAD-D-OUT.naf NAF-OUT.naf
NAF	1426989456.00	57.22	4208.75	0.63	671.05	./ennaf --temp-dir tmp/ --fastq --level 22 -o NAF-OUT.naf ERR3307082.fastq	./unnaf -o NAD-D-OUT.naf NAF-OUT.naf
LZMA	1732489188.00	12.05	10.75	2.65	3.38	./xz -1 -f -k ERR3307082.fastq.orig	./xz -f -k -d ERR3307082.fastq.orig.xz
LZMA	1719161352.00	16.72	18.37	2.52	4.37	./xz -2 -f -k ERR3307082.fastq.orig	./xz -f -k -d ERR3307082.fastq.orig.xz
LZMA	1711978116.00	28.15	33.38	2.45	6.38	./xz -3 -f -k ERR3307082.fastq.orig	./xz -f -k -d ERR3307082.fastq.orig.xz
LZMA	1557731164.00	62.15	49.50	2.47	6.38	./xz -4 -f -k ERR3307082.fastq.orig	./xz -f -k -d ERR3307082.fastq.orig.xz
LZMA	1550055232.00	74.28	95.37	2.45	10.37	./xz -5 -f -k ERR3307082.fastq.orig	./xz -f -k -d ERR3307082.fastq.orig.xz
LZMA	1546598340.00	74.98	95.50	2.43	10.37	./xz -6 -f -k ERR3307082.fastq.orig	./xz -f -k -d ERR3307082.fastq.orig.xz
LZMA	1538463616.00	84.88	185.25	2.47	18.37	./xz -7 -f -k ERR3307082.fastq.orig	./xz -f -k -d ERR3307082.fastq.orig.xz
LZMA	1527528028.00	94.57	357.00	2.43	34.37	./xz -8 -f -k ERR3307082.fastq.orig	./xz -f -k -d ERR3307082.fastq.orig.xz
LZMA	1515828508.00	105.67	661.00	2.42	66.37	./xz -9 -f -k ERR3307082.fastq.orig	./xz -f -k -d ERR3307082.fastq.orig.xz
BZIP2	1560387887.00	8.28	2.38	3.35	1.75	./bzip2 -1 -f -k ERR3307082.fastq.orig	./bzip2 -f -k -d ERR3307082.fastq.orig.bz2
BZIP2	1551772578.00	8.27	3.25	3.45	2.13	./bzip2 -2 -f -k ERR3307082.fastq.orig	./bzip2 -f -k -d ERR3307082.fastq.orig.bz2
BZIP2	1547322673.00	8.32	4.00	3.50	2.63	./bzip2 -3 -f -k ERR3307082.fastq.orig	./bzip2 -f -k -d ERR3307082.fastq.orig.bz2
BZIP2	1544304408.00	8.40	4.37	3.53	2.88	./bzip2 -4 -f -k ERR3307082.fastq.orig	./bzip2 -f -k -d ERR3307082.fastq.orig.bz2
BZIP2	1541869575.00	8.52	5.12	3.55	3.25	./bzip2 -5 -f -k ERR3307082.fastq.orig	./bzip2 -f -k -d ERR3307082.fastq.orig.bz2
BZIP2	1539947704.00	8.70	5.75	3.55	3.75	./bzip2 -6 -f -k ERR3307082.fastq.orig	./bzip2 -f -k -d ERR3307082.fastq.orig.bz2
BZIP2	1538379988.00	8.77	6.38	3.57	4.12	./bzip2 -7 -f -k ERR3307082.fastq.orig	./bzip2 -f -k -d ERR3307082.fastq.orig.bz2
BZIP2	1537017680.00	8.78	7.13	3.57	4.50	./bzip2 -8 -f -k ERR3307082.fastq.orig	./bzip2 -f -k -d ERR3307082.fastq.orig.bz2
BZIP2	1535794701.00	8.93	7.75	3.58	4.87	./bzip2 -9 -f -k ERR3307082.fastq.orig	./bzip2 -f -k -d ERR3307082.fastq.orig.bz2
BSC-m03	1339422395.00	50.92	9594.46	47.63	9594.70	./bsc-m03 e ERR3307082.fastq.orig ERR3307082.fastq.bsc -b800000000	./bsc-m03 d ERR3307082.fastq.bsc ERR3307082.fastq.out
BSC-m03	1352405633.00	50.68	4852.52	47.45	4852.73	./bsc-m03 e ERR3307082.fastq.orig ERR3307082.fastq.bsc -b400000000	./bsc-m03 d ERR3307082.fastq.bsc ERR3307082.fastq.out
BSC-m03	1404647406.00	48.35	68.40	46.40	68.73	./bsc-m03 e ERR3307082.fastq.orig ERR3307082.fastq.bsc -b4096000	./bsc-m03 d ERR3307082.fastq.bsc ERR3307082.fastq.out
PAQ8L	1372458978.00	841.88	40.00	846.25	40.00	./paq8l -1 ERR3307082.fastq	./paq8l -d ERR3307082.fastq.paq8l tmp_dir
JARVIS3	1403648000.00	7.87	210.37	8.07	210.00	./JARVIS3.sh --level 1 --block 600MB --threads 1 --fastq --input ERR3307082.fastq	./JARVIS3.sh --decompress --threads 1 --fastq --input ERR3307082.fastq.tar

JARVIS3	1403688960.00	8.13	210.37	8.17	210.00	./JARVIS3.sh --level 2 --block 600MB --threads 1 --fastq --input ERR3307082.fastq	./JARVIS3.sh --decompress --threads 1 --fastq --input ERR3307082.fastq.tar
JARVIS3	1393438720.00	7.78	402.25	8.13	402.00	./JARVIS3.sh --level 3 --block 600MB --threads 1 --fastq --input ERR3307082.fastq	./JARVIS3.sh --decompress --threads 1 --fastq --input ERR3307082.fastq.tar
JARVIS3	1384693760.00	7.83	1169.75	8.12	1169.50	./JARVIS3.sh --level 4 --block 600MB --threads 1 --fastq --input ERR3307082.fastq	./JARVIS3.sh --decompress --threads 1 --fastq --input ERR3307082.fastq.tar
JARVIS3	1387499520.00	7.97	210.37	8.20	210.00	./JARVIS3.sh --level 5 --block 600MB --threads 1 --fastq --input ERR3307082.fastq	./JARVIS3.sh --decompress --threads 1 --fastq --input ERR3307082.fastq.tar
JARVIS3	1369139200.00	10.40	402.25	10.37	402.00	./JARVIS3.sh --level 6 --block 600MB --threads 1 --fastq --input ERR3307082.fastq	./JARVIS3.sh --decompress --threads 1 --fastq --input ERR3307082.fastq.tar
JARVIS3	1365514240.00	7.92	402.25	8.22	402.12	./JARVIS3.sh --level 7 --block 600MB --threads 1 --fastq --input ERR3307082.fastq	./JARVIS3.sh --decompress --threads 1 --fastq --input ERR3307082.fastq.tar
JARVIS3	1357301760.00	7.78	1169.88	8.12	1169.50	./JARVIS3.sh --level 8 --block 600MB --threads 1 --fastq --input ERR3307082.fastq	./JARVIS3.sh --decompress --threads 1 --fastq --input ERR3307082.fastq.tar
JARVIS3	1344368640.00	9.82	1170.00	9.63	1169.38	./JARVIS3.sh --level 9 --block 600MB --threads 1 --fastq --input ERR3307082.fastq	./JARVIS3.sh --decompress --threads 1 --fastq --input ERR3307082.fastq.tar
JARVIS3	1368504320.00	9.87	210.37	9.72	210.00	./JARVIS3.sh --level 10 --block 600MB --threads 1 --fastq --input ERR3307082.fastq	./JARVIS3.sh --decompress --threads 1 --fastq --input ERR3307082.fastq.tar
JARVIS3	1340272640.00	14.77	913.87	14.30	913.87	./JARVIS3.sh --level 11 --block 600MB --threads 1 --fastq --input ERR3307082.fastq	./JARVIS3.sh --decompress --threads 1 --fastq --input ERR3307082.fastq.tar
JARVIS3	1352714240.00	15.63	402.37	15.62	401.87	./JARVIS3.sh --level 12 --block 600MB --threads 1 --fastq --input ERR3307082.fastq	./JARVIS3.sh --decompress --threads 1 --fastq --input ERR3307082.fastq.tar
JARVIS3	1350922240.00	17.15	402.25	17.23	402.12	./JARVIS3.sh --level 13 --block 600MB --threads 1 --fastq --input ERR3307082.fastq	./JARVIS3.sh --decompress --threads 1 --fastq --input ERR3307082.fastq.tar
JARVIS3	1380280320.00	10.38	338.25	10.03	338.25	./JARVIS3.sh --level 14 --block 600MB --threads 1 --fastq --input ERR3307082.fastq	./JARVIS3.sh --decompress --threads 1 --fastq --input ERR3307082.fastq.tar
JARVIS3	1365944320.00	11.20	402.25	10.87	402.12	./JARVIS3.sh --level 15 --block 600MB --threads 1 --fastq --input ERR3307082.fastq	./JARVIS3.sh --decompress --threads 1 --fastq --input ERR3307082.fastq.tar
JARVIS3	1363056640.00	12.08	338.50	12.00	338.12	./JARVIS3.sh --level 16 --block 600MB --threads 1 --fastq --input ERR3307082.fastq	./JARVIS3.sh --decompress --threads 1 --fastq --input ERR3307082.fastq.tar
JARVIS3	1346068480.00	12.03	1170.00	12.45	1169.88	./JARVIS3.sh --level 17 --block 600MB --threads 1 --fastq --input ERR3307082.fastq	./JARVIS3.sh --decompress --threads 1 --fastq --input ERR3307082.fastq.tar
JARVIS3	1345218560.00	12.35	1170.00	12.47	1169.62	./JARVIS3.sh --level 18 --block 600MB --threads 1 --fastq --input ERR3307082.fastq	./JARVIS3.sh --decompress --threads 1 --fastq --input ERR3307082.fastq.tar
JARVIS3	1345495040.00	15.60	402.37	16.07	402.25	./JARVIS3.sh --level 19 --block 600MB --threads 1 --fastq --input ERR3307082.fastq	./JARVIS3.sh --decompress --threads 1 --fastq --input ERR3307082.fastq.tar
JARVIS3	1333975040.00	25.55	466.37	24.93	466.12	./JARVIS3.sh --level 20 --block 600MB --threads 1 --fastq --input ERR3307082.fastq	./JARVIS3.sh --decompress --threads 1 --fastq --input ERR3307082.fastq.tar
JARVIS3	1325895680.00	31.30	530.12	31.37	530.12	./JARVIS3.sh --level 21 --block 600MB --threads 1 --fastq --input ERR3307082.fastq	./JARVIS3.sh --decompress --threads 1 --fastq --input ERR3307082.fastq.tar
JARVIS3	1325813760.00	31.63	530.37	31.48	530.12	./JARVIS3.sh --level 22 --block 600MB --threads 1 --fastq --input ERR3307082.fastq	./JARVIS3.sh --decompress --threads 1 --fastq --input ERR3307082.fastq.tar
JARVIS3	1324800000.00	31.47	594.25	31.80	594.12	./JARVIS3.sh --level 23 --block 600MB --threads 1 --fastq --input ERR3307082.fastq	./JARVIS3.sh --decompress --threads 1 --fastq --input ERR3307082.fastq.tar
JARVIS3	1333094400.00	13.38	4234.75	12.90	4234.63	./JARVIS3.sh --level 24 --block 600MB --threads 1 --fastq --input ERR3307082.fastq	./JARVIS3.sh --decompress --threads 1 --fastq --input ERR3307082.fastq.tar
JARVIS3	1320919040.00	35.52	4234.87	35.53	4234.63	./JARVIS3.sh --level 25 --block 600MB --threads 1 --fastq --input ERR3307082.fastq	./JARVIS3.sh --decompress --threads 1 --fastq --input ERR3307082.fastq.tar
JARVIS3	1307084800.00	46.63	4235.00	46.52	4234.75	./JARVIS3.sh --level 26 --block 600MB --threads 1 --fastq --input ERR3307082.fastq	./JARVIS3.sh --decompress --threads 1 --fastq --input ERR3307082.fastq.tar
SPRING	1395456000.00	8.98	640.04	5.30	632.97	./spring -c -t 1 --long -i ERR3307082.fastq -o ERR3307082.fastq.spring	./spring -d -t 1 --long -i ERR3307082.fastq.spring -o ERR3307082.fastq.out
LEON	1600058824.00	16.58	3676.08	8.33	1778.11	./leon -nb-cores 1 -lossless -c -file ERR3307082.fastq	./leon -nb-cores 1 -d -file ERR3307082.fastq.leon

Table S5: Results obtained when compressing the UCSC hg38 7way knownCanonical-exonNuc [10].

**Compressor**	**Compressed data size (bytes)**	**Compression time (minutes)**	**Compression memory (MB)**	**Decompression time (minutes)**	**Decompression memory (MB)**	**Compression options**	**Decompression options**
AGC	271279862.00	168.55	4779.16	0.25	2765.99	./agc create -t 1 EXON.fa	./agc getcol -t 1 AGC-OUT.agc
JARVIS2	41717760.00	20.73	1090.00	20.77	1089.63	./JARVIS2.sh --fasta --level 1 --block 600MB --threads 1 --fasta --input EXON.fa	./JARVIS2.sh --decompress --threads 1 --fasta --input EXON.fa.tar
JARVIS2	41717760.00	20.77	1089.75	20.75	1089.75	./JARVIS2.sh --fasta --level 2 --block 600MB --threads 1 --fasta --input EXON.fa	./JARVIS2.sh --decompress --threads 1 --fasta --input EXON.fa.tar
JARVIS2	41717760.00	20.75	1090.13	20.80	1090.00	./JARVIS2.sh --fasta --level 3 --block 600MB --threads 1 --fasta --input EXON.fa	./JARVIS2.sh --decompress --threads 1 --fasta --input EXON.fa.tar
JARVIS2	41717760.00	20.78	1090.13	20.78	1089.63	./JARVIS2.sh --fasta --level 7 --block 600MB --threads 1 --fasta --input EXON.fa	./JARVIS2.sh --decompress --threads 1 --fasta --input EXON.fa.tar
NAF	53460638.00	0.02	15.37	0.03	108.09	./ennaf --temp-dir tmp/ --dna --level 1 -o NAF-OUT.naf EXON.fa	./unnaf -o NAD-D-OUT.naf NAF-OUT.naf
NAF	54938582.00	0.02	18.87	0.03	108.66	./ennaf --temp-dir tmp/ --dna --level 2 -o NAF-OUT.naf EXON.fa	./unnaf -o NAD-D-OUT.naf NAF-OUT.naf
NAF	58442264.00	0.02	25.25	0.03	109.57	./ennaf --temp-dir tmp/ --dna --level 3 -o NAF-OUT.naf EXON.fa	./unnaf -o NAD-D-OUT.naf NAF-OUT.naf
NAF	61355644.00	0.03	31.63	0.03	109.57	./ennaf --temp-dir tmp/ --dna --level 4 -o NAF-OUT.naf EXON.fa	./unnaf -o NAD-D-OUT.naf NAF-OUT.naf
NAF	60405621.00	0.03	36.75	0.03	109.53	./ennaf --temp-dir tmp/ --dna --level 5 -o NAF-OUT.naf EXON.fa	./unnaf -o NAD-D-OUT.naf NAF-OUT.naf
NAF	57795188.00	0.12	149.37	0.03	111.64	./ennaf --temp-dir tmp/ --dna --level 11 -o NAF-OUT.naf EXON.fa	./unnaf -o NAD-D-OUT.naf NAF-OUT.naf
NAF	44720149.00	1.88	444.87	0.03	115.62	./ennaf --temp-dir tmp/ --dna --level 19 -o NAF-OUT.naf EXON.fa	./unnaf -o NAD-D-OUT.naf NAF-OUT.naf
NAF	44470346.00	2.88	3460.37	0.03	230.41	./ennaf --temp-dir tmp/ --dna --level 22 -o NAF-OUT.naf EXON.fa	./unnaf -o NAD-D-OUT.naf NAF-OUT.naf
LZMA	57665800.00	0.52	10.63	0.10	3.25	./xz -1 -f -k EXON.fa.orig	./xz -f -k -d EXON.fa.orig.xz
LZMA	57447176.00	0.68	18.00	0.10	4.25	./xz -2 -f -k EXON.fa.orig	./xz -f -k -d EXON.fa.orig.xz
LZMA	57272824.00	0.95	32.00	0.08	6.25	./xz -3 -f -k EXON.fa.orig	./xz -f -k -d EXON.fa.orig.xz
LZMA	45976460.00	3.47	48.00	0.10	6.25	./xz -4 -f -k EXON.fa.orig	./xz -f -k -d EXON.fa.orig.xz
LZMA	43436424.00	5.03	88.88	0.08	10.25	./xz -5 -f -k EXON.fa.orig	./xz -f -k -d EXON.fa.orig.xz
LZMA	43089828.00	5.70	89.00	0.08	10.25	./xz -6 -f -k EXON.fa.orig	./xz -f -k -d EXON.fa.orig.xz
LZMA	42382468.00	6.23	167.62	0.08	18.25	./xz -7 -f -k EXON.fa.orig	./xz -f -k -d EXON.fa.orig.xz
LZMA	42166632.00	6.73	321.25	0.08	34.25	./xz -8 -f -k EXON.fa.orig	./xz -f -k -d EXON.fa.orig.xz
LZMA	42049312.00	7.35	625.13	0.08	66.13	./xz -9 -f -k EXON.fa.orig	./xz -f -k -d EXON.fa.orig.xz
BZIP2	69504800.00	0.77	2.38	0.20	1.87	./bzip2 -1 -f -k EXON.fa.orig	./bzip2 -f -k -d EXON.fa.orig.bz2
BZIP2	69623156.00	0.77	3.00	0.22	2.25	./bzip2 -2 -f -k EXON.fa.orig	./bzip2 -f -k -d EXON.fa.orig.bz2
BZIP2	69821076.00	0.77	3.75	0.22	2.63	./bzip2 -3 -f -k EXON.fa.orig	./bzip2 -f -k -d EXON.fa.orig.bz2
BZIP2	70030507.00	0.78	4.37	0.22	3.00	./bzip2 -4 -f -k EXON.fa.orig	./bzip2 -f -k -d EXON.fa.orig.bz2
BZIP2	70185950.00	0.78	5.12	0.22	3.38	./bzip2 -5 -f -k EXON.fa.orig	./bzip2 -f -k -d EXON.fa.orig.bz2
BZIP2	70276294.00	0.80	5.75	0.22	3.75	./bzip2 -6 -f -k EXON.fa.orig	./bzip2 -f -k -d EXON.fa.orig.bz2
BZIP2	70467268.00	0.80	6.38	0.22	4.12	./bzip2 -7 -f -k EXON.fa.orig	./bzip2 -f -k -d EXON.fa.orig.bz2
BZIP2	70586737.00	0.82	7.13	0.22	4.50	./bzip2 -8 -f -k EXON.fa.orig	./bzip2 -f -k -d EXON.fa.orig.bz2
BZIP2	70674752.00	0.82	7.75	0.22	4.87	./bzip2 -9 -f -k EXON.fa.orig	./bzip2 -f -k -d EXON.fa.orig.bz2
BSC-m03	61217312.00	3.65	4039.41	3.27	3772.88	./bsc-m03 e EXON.fa.orig EXON.fa.bsc -b800000000	./bsc-m03 d EXON.fa.bsc EXON.fa.out
BSC-m03	61217312.00	3.63	4039.51	3.28	3772.75	./bsc-m03 e EXON.fa.orig EXON.fa.bsc -b400000000	./bsc-m03 d EXON.fa.bsc EXON.fa.out
BSC-m03	59218675.00	3.35	62.58	3.03	62.86	./bsc-m03 e EXON.fa.orig EXON.fa.bsc -b4096000	./bsc-m03 d EXON.fa.bsc EXON.fa.out
MFCompress	68041267.00	0.83	567.37	0.82	521.25	./MFCompressC -v -1 -p 1 -t 1 -o MFC-OUT.mfc EXON.fa	./MFCompressD -o MFC-OUT.d MFC-OUT.mfc

MFCompress	67601187.00	0.90	568.00	0.88	521.75	./MFCompressC -v -2 -p 1 -t 1 -o MFC-OUT.mfc EXON.fa	./MFCompressD -o MFC-OUT.d MFC-OUT.mfc
MFCompress	61947286.00	1.33	2375.50	1.12	2329.50	./MFCompressC -v -3 -p 1 -t 1 -o MFC-OUT.mfc EXON.fa	./MFCompressD -o MFC-OUT.d MFC-OUT.mfc
PAQ8L	43300393.00	57.85	39.63	57.90	39.50	./paq8l -1 EXON.fa	./paq8l -d EXON.fa.paq8l tmp_dir
JARVIS3	44881920.00	0.45	124.62	0.43	124.38	./JARVIS3.sh --level 1 --block 600MB --threads 1 --fasta --input EXON.fa	./JARVIS3.sh --decompress --threads 1 --fasta --input EXON.fa.tar
JARVIS3	44912640.00	0.50	124.75	0.43	124.38	./JARVIS3.sh --level 2 --block 600MB --threads 1 --fasta --input EXON.fa	./JARVIS3.sh --decompress --threads 1 --fasta --input EXON.fa.tar
JARVIS3	44984320.00	0.47	316.50	0.47	316.38	./JARVIS3.sh --level 3 --block 600MB --threads 1 --fasta --input EXON.fa	./JARVIS3.sh --decompress --threads 1 --fasta --input EXON.fa.tar
JARVIS3	44963840.00	0.50	1084.62	0.48	1084.25	./JARVIS3.sh --level 4 --block 600MB --threads 1 --fasta --input EXON.fa	./JARVIS3.sh --decompress --threads 1 --fasta --input EXON.fa.tar
JARVIS3	43898880.00	0.52	124.50	0.50	124.25	./JARVIS3.sh --level 5 --block 600MB --threads 1 --fasta --input EXON.fa	./JARVIS3.sh --decompress --threads 1 --fasta --input EXON.fa.tar
JARVIS3	44103680.00	0.87	316.62	0.87	316.25	./JARVIS3.sh --level 6 --block 600MB --threads 1 --fasta --input EXON.fa	./JARVIS3.sh --decompress --threads 1 --fasta --input EXON.fa.tar
JARVIS3	43704320.00	0.58	316.38	0.60	316.25	./JARVIS3.sh --level 7 --block 600MB --threads 1 --fasta --input EXON.fa	./JARVIS3.sh --decompress --threads 1 --fasta --input EXON.fa.tar
JARVIS3	43765760.00	0.63	1084.50	0.63	1084.38	./JARVIS3.sh --level 8 --block 600MB --threads 1 --fasta --input EXON.fa	./JARVIS3.sh --decompress --threads 1 --fasta --input EXON.fa.tar
JARVIS3	43550720.00	0.77	1084.50	0.75	1084.25	./JARVIS3.sh --level 9 --block 600MB --threads 1 --fasta --input EXON.fa	./JARVIS3.sh --decompress --threads 1 --fasta --input EXON.fa.tar
JARVIS3	43325440.00	0.77	124.50	0.75	124.00	./JARVIS3.sh --level 10 --block 600MB --threads 1 --fasta --input EXON.fa	./JARVIS3.sh --decompress --threads 1 --fasta --input EXON.fa.tar
JARVIS3	43130880.00	1.15	828.50	1.13	828.25	./JARVIS3.sh --level 11 --block 600MB --threads 1 --fasta --input EXON.fa	./JARVIS3.sh --decompress --threads 1 --fasta --input EXON.fa.tar
JARVIS3	42874880.00	1.27	316.25	1.28	316.25	./JARVIS3.sh --level 12 --block 600MB --threads 1 --fasta --input EXON.fa	./JARVIS3.sh --decompress --threads 1 --fasta --input EXON.fa.tar
JARVIS3	42762240.00	1.40	316.38	1.43	316.25	./JARVIS3.sh --level 13 --block 600MB --threads 1 --fasta --input EXON.fa	./JARVIS3.sh --decompress --threads 1 --fasta --input EXON.fa.tar
JARVIS3	44523520.00	0.93	252.75	0.93	252.38	./JARVIS3.sh --level 14 --block 600MB --threads 1 --fasta --input EXON.fa	./JARVIS3.sh --decompress --threads 1 --fasta --input EXON.fa.tar
JARVIS3	43653120.00	1.00	316.75	1.00	316.38	./JARVIS3.sh --level 15 --block 600MB --threads 1 --fasta --input EXON.fa	./JARVIS3.sh --decompress --threads 1 --fasta --input EXON.fa.tar
JARVIS3	43100160.00	1.07	252.75	1.10	252.25	./JARVIS3.sh --level 16 --block 600MB --threads 1 --fasta --input EXON.fa	./JARVIS3.sh --decompress --threads 1 --fasta --input EXON.fa.tar
JARVIS3	43079680.00	1.12	1084.38	1.13	1084.25	./JARVIS3.sh --level 17 --block 600MB --threads 1 --fasta --input EXON.fa	./JARVIS3.sh --decompress --threads 1 --fasta --input EXON.fa.tar
JARVIS3	43018240.00	1.13	1084.75	1.15	1084.62	./JARVIS3.sh --level 18 --block 600MB --threads 1 --fasta --input EXON.fa	./JARVIS3.sh --decompress --threads 1 --fasta --input EXON.fa.tar
JARVIS3	42373120.00	1.38	316.50	1.38	316.50	./JARVIS3.sh --level 19 --block 600MB --threads 1 --fasta --input EXON.fa	./JARVIS3.sh --decompress --threads 1 --fasta --input EXON.fa.tar
JARVIS3	41666560.00	2.15	380.62	2.18	380.38	./JARVIS3.sh --level 20 --block 600MB --threads 1 --fasta --input EXON.fa	./JARVIS3.sh --decompress --threads 1 --fasta --input EXON.fa.tar
JARVIS3	41594880.00	3.60	444.50	3.63	444.50	./JARVIS3.sh --level 21 --block 600MB --threads 1 --fasta --input EXON.fa	./JARVIS3.sh --decompress --threads 1 --fasta --input EXON.fa.tar
JARVIS3	41728000.00	4.88	444.75	4.93	444.38	./JARVIS3.sh --level 22 --block 600MB --threads 1 --fasta --input EXON.fa	./JARVIS3.sh --decompress --threads 1 --fasta --input EXON.fa.tar
JARVIS3	41881600.00	5.78	508.50	5.85	508.50	./JARVIS3.sh --level 23 --block 600MB --threads 1 --fasta --input EXON.fa	./JARVIS3.sh --decompress --threads 1 --fasta --input EXON.fa.tar
JARVIS3	42823680.00	1.13	4155.87	1.13	4155.87	./JARVIS3.sh --level 24 --block 600MB --threads 1 --fasta --input EXON.fa	./JARVIS3.sh --decompress --threads 1 --fasta --input EXON.fa.tar
JARVIS3	41328640.00	4.10	4156.00	4.20	4155.63	./JARVIS3.sh --level 25 --block 600MB --threads 1 --fasta --input EXON.fa	./JARVIS3.sh --decompress --threads 1 --fasta --input EXON.fa.tar
JARVIS3	40919040.00	5.25	4156.13	5.23	4155.87	./JARVIS3.sh --level 26 --block 600MB --threads 1 --fasta --input EXON.fa	./JARVIS3.sh --decompress --threads 1 --fasta --input EXON.fa.tar

Table S6: Results obtained when compressing the Human T2T genome.

**Compressor**	**Compressed data size (bytes)**	**Compression time (minutes)**	**Compression memory (MB)**	**Decompression time (minutes)**	**Decompression memory (MB)**	**Compression options**	**Decompression options**
AGC	707323971.00	6.07	27170.10	0.18	1900.10	./agc create -t 1 HUMAN.fa	./agc getcol -t 1 AGC-OUT.agc
JARVIS2	602275840.00	177.68	1213.75	179.13	1213.50	./JARVIS2.sh --fasta --level 1 --block 600MB --threads 1 --fasta --input HUMAN.fa	./JARVIS2.sh --decompress --threads 1 --fasta --input HUMAN.fa.tar
JARVIS2	602275840.00	177.27	1213.75	178.17	1213.87	./JARVIS2.sh --fasta --level 2 --block 600MB --threads 1 --fasta --input HUMAN.fa	./JARVIS2.sh --decompress --threads 1 --fasta --input HUMAN.fa.tar
JARVIS2	602275840.00	177.58	1214.00	179.65	1213.75	./JARVIS2.sh --fasta --level 3 --block 600MB --threads 1 --fasta --input HUMAN.fa	./JARVIS2.sh --decompress --threads 1 --fasta --input HUMAN.fa.tar
JARVIS2	602275840.00	177.35	1213.62	178.07	1213.62	./JARVIS2.sh --fasta --level 7 --block 600MB --threads 1 --fasta --input HUMAN.fa	./JARVIS2.sh --decompress --threads 1 --fasta --input HUMAN.fa.tar
NAF	717725697.00	0.18	9.12	0.37	46.00	./ennaf --temp-dir tmp/ --dna --level 1 -o NAF-OUT.naf HUMAN.fa	./unnaf -o NAD-D-OUT.naf NAF-OUT.naf
NAF	714648912.00	0.27	11.13	0.37	46.00	./ennaf --temp-dir tmp/ --dna --level 2 -o NAF-OUT.naf HUMAN.fa	./unnaf -o NAD-D-OUT.naf NAF-OUT.naf
NAF	749159327.00	0.35	15.63	0.37	46.12	./ennaf --temp-dir tmp/ --dna --level 3 -o NAF-OUT.naf HUMAN.fa	./unnaf -o NAD-D-OUT.naf NAF-OUT.naf
NAF	777588732.00	0.40	22.00	0.38	46.12	./ennaf --temp-dir tmp/ --dna --level 4 -o NAF-OUT.naf HUMAN.fa	./unnaf -o NAD-D-OUT.naf NAF-OUT.naf
NAF	752707527.00	0.43	26.87	0.38	45.87	./ennaf --temp-dir tmp/ --dna --level 5 -o NAF-OUT.naf HUMAN.fa	./unnaf -o NAD-D-OUT.naf NAF-OUT.naf
NAF	738529888.00	1.32	136.00	0.38	46.12	./ennaf --temp-dir tmp/ --dna --level 11 -o NAF-OUT.naf HUMAN.fa	./unnaf -o NAD-D-OUT.naf NAF-OUT.naf
NAF	672255109.00	19.28	426.25	0.35	45.75	./ennaf --temp-dir tmp/ --dna --level 19 -o NAF-OUT.naf HUMAN.fa	./unnaf -o NAD-D-OUT.naf NAF-OUT.naf
NAF	659114290.00	32.78	3404.25	0.40	155.96	./ennaf --temp-dir tmp/ --dna --level 22 -o NAF-OUT.naf HUMAN.fa	./unnaf -o NAD-D-OUT.naf NAF-OUT.naf
LZMA	950611700.00	8.12	10.25	1.62	3.25	./xz -1 -f -k HUMAN.fa.orig	./xz -f -k -d HUMAN.fa.orig.xz
LZMA	928232860.00	10.70	16.37	1.48	4.25	./xz -2 -f -k HUMAN.fa.orig	./xz -f -k -d HUMAN.fa.orig.xz
LZMA	915059364.00	15.05	27.62	1.45	6.25	./xz -3 -f -k HUMAN.fa.orig	./xz -f -k -d HUMAN.fa.orig.xz
LZMA	789971336.00	56.68	43.75	1.17	6.25	./xz -4 -f -k HUMAN.fa.orig	./xz -f -k -d HUMAN.fa.orig.xz
LZMA	763881052.00	70.42	82.13	1.13	10.25	./xz -5 -f -k HUMAN.fa.orig	./xz -f -k -d HUMAN.fa.orig.xz
LZMA	760250800.00	73.93	81.88	1.13	10.25	./xz -6 -f -k HUMAN.fa.orig	./xz -f -k -d HUMAN.fa.orig.xz
LZMA	754822576.00	82.23	158.00	1.15	18.25	./xz -7 -f -k HUMAN.fa.orig	./xz -f -k -d HUMAN.fa.orig.xz
LZMA	748803848.00	89.28	310.12	1.18	34.25	./xz -8 -f -k HUMAN.fa.orig	./xz -f -k -d HUMAN.fa.orig.xz
LZMA	741982924.00	97.87	613.87	1.20	66.25	./xz -9 -f -k HUMAN.fa.orig	./xz -f -k -d HUMAN.fa.orig.xz
BZIP2	865659495.00	7.37	2.38	2.47	1.87	./bzip2 -1 -f -k HUMAN.fa.orig	./bzip2 -f -k -d HUMAN.fa.orig.bz2
BZIP2	856453342.00	7.45	3.00	2.55	2.25	./bzip2 -2 -f -k HUMAN.fa.orig	./bzip2 -f -k -d HUMAN.fa.orig.bz2
BZIP2	851720650.00	7.62	3.75	2.58	2.63	./bzip2 -3 -f -k HUMAN.fa.orig	./bzip2 -f -k -d HUMAN.fa.orig.bz2
BZIP2	848544681.00	7.77	4.37	2.60	3.00	./bzip2 -4 -f -k HUMAN.fa.orig	./bzip2 -f -k -d HUMAN.fa.orig.bz2
BZIP2	846134861.00	7.90	5.12	2.63	3.25	./bzip2 -5 -f -k HUMAN.fa.orig	./bzip2 -f -k -d HUMAN.fa.orig.bz2
BZIP2	844197013.00	8.03	5.75	2.62	3.75	./bzip2 -6 -f -k HUMAN.fa.orig	./bzip2 -f -k -d HUMAN.fa.orig.bz2
BZIP2	842496570.00	8.08	6.38	2.62	4.12	./bzip2 -7 -f -k HUMAN.fa.orig	./bzip2 -f -k -d HUMAN.fa.orig.bz2
BZIP2	841139353.00	8.17	7.13	2.63	4.50	./bzip2 -8 -f -k HUMAN.fa.orig	./bzip2 -f -k -d HUMAN.fa.orig.bz2
BZIP2	839894118.00	8.25	7.75	2.65	4.87	./bzip2 -9 -f -k HUMAN.fa.orig	./bzip2 -f -k -d HUMAN.fa.orig.bz2
BSC-m03	683454523.00	38.40	10121.46	34.92	10121.28	./bsc-m03 e HUMAN.fa.orig HUMAN.fa.bsc -b800000000	./bsc-m03 d HUMAN.fa.bsc HUMAN.fa.out
BSC-m03	688715181.00	38.40	5067.20	34.95	5067.11	./bsc-m03 e HUMAN.fa.orig HUMAN.fa.bsc -b400000000	./bsc-m03 d HUMAN.fa.bsc HUMAN.fa.out
BSC-m03	724496261.00	36.67	71.57	33.70	72.12	./bsc-m03 e HUMAN.fa.orig HUMAN.fa.bsc -b4096000	./bsc-m03 d HUMAN.fa.bsc HUMAN.fa.out
MFCompress	646565512.00	9.30	516.00	8.30	514.75	./MFCompressC -v -1 -p 1 -t 1 -o MFC-OUT.mfc HUMAN.fa	./MFCompressD -o MFC-OUT.d MFC-OUT.mfc

MFCompress	640649639.00	9.88	516.25	9.47	515.38	./MFCompressC -v -2 -p 1 -t 1 -o MFC-OUT.mfc HUMAN.fa	./MFCompressD -o MFC-OUT.d MFC-OUT.mfc
MFCompress	628561658.00	17.02	2324.00	11.98	2323.13	./MFCompressC -v -3 -p 1 -t 1 -o MFC-OUT.mfc HUMAN.fa	./MFCompressD -o MFC-OUT.d MFC-OUT.mfc
PAQ8L	708194277.00	670.25	40.13	671.27	39.87	./paq8l -1 HUMAN.fa	./paq8l -d HUMAN.fa.paq8l tmp_dir
JARVIS3	713717760.00	6.23	210.25	6.55	210.12	./JARVIS3.sh --level 1 --block 600MB --threads 1 --fasta --input HUMAN.fa	./JARVIS3.sh --decompress --threads 1 --fasta --input HUMAN.fa.tar
JARVIS3	710778880.00	6.60	210.37	6.62	210.00	./JARVIS3.sh --level 2 --block 600MB --threads 1 --fasta --input HUMAN.fa	./JARVIS3.sh --decompress --threads 1 --fasta --input HUMAN.fa.tar
JARVIS3	703375360.00	6.78	402.50	6.82	402.12	./JARVIS3.sh --level 3 --block 600MB --threads 1 --fasta --input HUMAN.fa	./JARVIS3.sh --decompress --threads 1 --fasta --input HUMAN.fa.tar
JARVIS3	696688640.00	7.22	1170.50	7.17	1170.00	./JARVIS3.sh --level 4 --block 600MB --threads 1 --fasta --input HUMAN.fa	./JARVIS3.sh --decompress --threads 1 --fasta --input HUMAN.fa.tar
JARVIS3	681850880.00	7.80	210.37	7.92	210.12	./JARVIS3.sh --level 5 --block 600MB --threads 1 --fasta --input HUMAN.fa	./JARVIS3.sh --decompress --threads 1 --fasta --input HUMAN.fa.tar
JARVIS3	661053440.00	14.70	402.37	14.70	402.12	./JARVIS3.sh --level 6 --block 600MB --threads 1 --fasta --input HUMAN.fa	./JARVIS3.sh --decompress --threads 1 --fasta --input HUMAN.fa.tar
JARVIS3	665425920.00	9.65	402.37	9.77	402.00	./JARVIS3.sh --level 7 --block 600MB --threads 1 --fasta --input HUMAN.fa	./JARVIS3.sh --decompress --threads 1 --fasta --input HUMAN.fa.tar
JARVIS3	661575680.00	10.23	1170.37	10.30	1170.12	./JARVIS3.sh --level 8 --block 600MB --threads 1 --fasta --input HUMAN.fa	./JARVIS3.sh --decompress --threads 1 --fasta --input HUMAN.fa.tar
JARVIS3	651089920.00	13.82	1170.37	13.85	1170.00	./JARVIS3.sh --level 9 --block 600MB --threads 1 --fasta --input HUMAN.fa	./JARVIS3.sh --decompress --threads 1 --fasta --input HUMAN.fa.tar
JARVIS3	657459200.00	13.73	210.37	13.63	210.00	./JARVIS3.sh --level 10 --block 600MB --threads 1 --fasta --input HUMAN.fa	./JARVIS3.sh --decompress --threads 1 --fasta --input HUMAN.fa.tar
JARVIS3	643614720.00	20.93	914.37	21.05	914.00	./JARVIS3.sh --level 11 --block 600MB --threads 1 --fasta --input HUMAN.fa	./JARVIS3.sh --decompress --threads 1 --fasta --input HUMAN.fa.tar
JARVIS3	645181440.00	22.90	402.37	23.03	402.00	./JARVIS3.sh --level 12 --block 600MB --threads 1 --fasta --input HUMAN.fa	./JARVIS3.sh --decompress --threads 1 --fasta --input HUMAN.fa.tar
JARVIS3	643840000.00	25.27	402.37	25.38	402.00	./JARVIS3.sh --level 13 --block 600MB --threads 1 --fasta --input HUMAN.fa	./JARVIS3.sh --decompress --threads 1 --fasta --input HUMAN.fa.tar
JARVIS3	656056320.00	14.20	338.50	14.75	338.25	./JARVIS3.sh --level 14 --block 600MB --threads 1 --fasta --input HUMAN.fa	./JARVIS3.sh --decompress --threads 1 --fasta --input HUMAN.fa.tar
JARVIS3	642836480.00	15.62	402.50	15.95	402.25	./JARVIS3.sh --level 15 --block 600MB --threads 1 --fasta --input HUMAN.fa	./JARVIS3.sh --decompress --threads 1 --fasta --input HUMAN.fa.tar
JARVIS3	638894080.00	17.20	338.50	17.57	338.12	./JARVIS3.sh --level 16 --block 600MB --threads 1 --fasta --input HUMAN.fa	./JARVIS3.sh --decompress --threads 1 --fasta --input HUMAN.fa.tar
JARVIS3	631459840.00	17.98	1170.63	18.33	1170.25	./JARVIS3.sh --level 17 --block 600MB --threads 1 --fasta --input HUMAN.fa	./JARVIS3.sh --decompress --threads 1 --fasta --input HUMAN.fa.tar
JARVIS3	631756800.00	18.15	1170.63	18.40	1170.37	./JARVIS3.sh --level 18 --block 600MB --threads 1 --fasta --input HUMAN.fa	./JARVIS3.sh --decompress --threads 1 --fasta --input HUMAN.fa.tar
JARVIS3	626800640.00	23.58	402.50	23.87	402.25	./JARVIS3.sh --level 19 --block 600MB --threads 1 --fasta --input HUMAN.fa	./JARVIS3.sh --decompress --threads 1 --fasta --input HUMAN.fa.tar
JARVIS3	619161600.00	38.07	466.50	38.38	466.25	./JARVIS3.sh --level 20 --block 600MB --threads 1 --fasta --input HUMAN.fa	./JARVIS3.sh --decompress --threads 1 --fasta --input HUMAN.fa.tar
JARVIS3	614461440.00	53.35	530.50	55.00	530.37	./JARVIS3.sh --level 21 --block 600MB --threads 1 --fasta --input HUMAN.fa	./JARVIS3.sh --decompress --threads 1 --fasta --input HUMAN.fa.tar
JARVIS3	613826560.00	61.27	530.50	61.68	530.25	./JARVIS3.sh --level 22 --block 600MB --threads 1 --fasta --input HUMAN.fa	./JARVIS3.sh --decompress --threads 1 --fasta --input HUMAN.fa.tar
JARVIS3	613406720.00	70.23	594.62	70.68	594.12	./JARVIS3.sh --level 23 --block 600MB --threads 1 --fasta --input HUMAN.fa	./JARVIS3.sh --decompress --threads 1 --fasta --input HUMAN.fa.tar
JARVIS3	623738880.00	19.33	4242.12	19.43	4242.00	./JARVIS3.sh --level 24 --block 600MB --threads 1 --fasta --input HUMAN.fa	./JARVIS3.sh --decompress --threads 1 --fasta --input HUMAN.fa.tar
JARVIS3	604661760.00	58.40	4242.12	59.28	4241.75	./JARVIS3.sh --level 25 --block 600MB --threads 1 --fasta --input HUMAN.fa	./JARVIS3.sh --decompress --threads 1 --fasta --input HUMAN.fa.tar
JARVIS3	598241280.00	78.33	4241.88	79.15	4241.88	./JARVIS3.sh --level 26 --block 600MB --threads 1 --fasta --input HUMAN.fa	./JARVIS3.sh --decompress --threads 1 --fasta --input HUMAN.fa.tar

Table S7: Results obtained when compressing the SRR1284073.

Compressor	Compressed data size (bytes)	Compression time (minutes)	Compression memory (MB)	Decompression time (minutes)	Decompression memory (MB)	Compression options	Decompression options
JARVIS2	318187520.00	23.53	1247.13	23.38	1247.13	./JARVIS2.sh --fastq --level 1 --block 600MB --threads 1 --fastq --input SRR1284073.fastq	./JARVIS2.sh --decompress --threads 1 --fastq --input SRR1284073.fastq.tar
JARVIS2	318187520.00	23.08	1247.25	23.42	1246.87	./JARVIS2.sh --fastq --level 2 --block 600MB --threads 1 --fastq --input SRR1284073.fastq	./JARVIS2.sh --decompress --threads 1 --fastq --input SRR1284073.fastq.tar
JARVIS2	318187520.00	23.30	1246.87	23.58	1247.25	./JARVIS2.sh --fastq --level 3 --block 600MB --threads 1 --fastq --input SRR1284073.fastq	./JARVIS2.sh --decompress --threads 1 --fastq --input SRR1284073.fastq.tar
JARVIS2	318187520.00	23.42	1246.87	23.57	1247.25	./JARVIS2.sh --fastq --level 7 --block 600MB --threads 1 --fastq --input SRR1284073.fastq	./JARVIS2.sh --decompress --threads 1 --fastq --input SRR1284073.fastq.tar
NAF	358315608.00	0.07	15.75	0.02	133.26	./ennaf --temp-dir tmp/ --fastq --level 1 -o NAF-OUT.naf SRR1284073.fastq	./unnaf -o NAD-D-OUT.naf NAF-OUT.naf
NAF	363458960.00	0.12	19.38	0.15	134.51	./ennaf --temp-dir tmp/ --fastq --level 2 -o NAF-OUT.naf SRR1284073.fastq	./unnaf -o NAD-D-OUT.naf NAF-OUT.naf
NAF	382379409.00	0.17	26.87	0.13	141.50	./ennaf --temp-dir tmp/ --fastq --level 3 -o NAF-OUT.naf SRR1284073.fastq	./unnaf -o NAD-D-OUT.naf NAF-OUT.naf
NAF	393874515.00	0.17	34.50	0.15	148.37	./ennaf --temp-dir tmp/ --fastq --level 4 -o NAF-OUT.naf SRR1284073.fastq	./unnaf -o NAD-D-OUT.naf NAF-OUT.naf
NAF	381469559.00	0.20	40.50	0.15	144.75	./ennaf --temp-dir tmp/ --fastq --level 5 -o NAF-OUT.naf SRR1284073.fastq	./unnaf -o NAD-D-OUT.naf NAF-OUT.naf
NAF	378430963.00	0.70	170.87	0.17	149.30	./ennaf --temp-dir tmp/ --fastq --level 11 -o NAF-OUT.naf SRR1284073.fastq	./unnaf -o NAD-D-OUT.naf NAF-OUT.naf
NAF	350653660.00	10.13	517.75	0.17	146.82	./ennaf --temp-dir tmp/ --fastq --level 19 -o NAF-OUT.naf SRR1284073.fastq	./unnaf -o NAD-D-OUT.naf NAF-OUT.naf
NAF	345076688.00	16.80	4166.87	0.17	383.19	./ennaf --temp-dir tmp/ --fastq --level 22 -o NAF-OUT.naf SRR1284073.fastq	./unnaf -o NAD-D-OUT.naf NAF-OUT.naf
LZMA	406215952.00	3.27	10.87	0.65	3.38	./xz -1 -f -k SRR1284073.fastq.orig	./xz -f -k -d SRR1284073.fastq.orig.xz
LZMA	403233028.00	4.43	18.25	0.60	4.25	./xz -2 -f -k SRR1284073.fastq.orig	./xz -f -k -d SRR1284073.fastq.orig.xz
LZMA	402008852.00	7.38	31.75	0.60	6.38	./xz -3 -f -k SRR1284073.fastq.orig	./xz -f -k -d SRR1284073.fastq.orig.xz
LZMA	372696012.00	18.95	47.87	0.57	6.38	./xz -4 -f -k SRR1284073.fastq.orig	./xz -f -k -d SRR1284073.fastq.orig.xz
LZMA	370183896.00	22.87	88.37	0.58	10.25	./xz -5 -f -k SRR1284073.fastq.orig	./xz -f -k -d SRR1284073.fastq.orig.xz
LZMA	370703196.00	22.85	88.25	0.60	10.25	./xz -6 -f -k SRR1284073.fastq.orig	./xz -f -k -d SRR1284073.fastq.orig.xz
LZMA	368639028.00	25.68	164.63	0.60	18.37	./xz -7 -f -k SRR1284073.fastq.orig	./xz -f -k -d SRR1284073.fastq.orig.xz
LZMA	366861204.00	28.65	316.87	0.60	34.25	./xz -8 -f -k SRR1284073.fastq.orig	./xz -f -k -d SRR1284073.fastq.orig.xz
LZMA	364649432.00	31.75	620.87	0.60	66.37	./xz -9 -f -k SRR1284073.fastq.orig	./xz -f -k -d SRR1284073.fastq.orig.xz
BZIP2	369216224.00	2.27	2.38	0.85	1.75	./bzip2 -1 -f -k SRR1284073.fastq.orig	./bzip2 -f -k -d SRR1284073.fastq.orig.bz2
BZIP2	367182186.00	2.27	3.13	0.88	2.00	./bzip2 -2 -f -k SRR1284073.fastq.orig	./bzip2 -f -k -d SRR1284073.fastq.orig.bz2
BZIP2	366276206.00	2.28	3.75	0.88	2.63	./bzip2 -3 -f -k SRR1284073.fastq.orig	./bzip2 -f -k -d SRR1284073.fastq.orig.bz2
BZIP2	365736357.00	2.32	4.37	0.90	3.00	./bzip2 -4 -f -k SRR1284073.fastq.orig	./bzip2 -f -k -d SRR1284073.fastq.orig.bz2
BZIP2	365351440.00	2.35	5.12	0.92	3.25	./bzip2 -5 -f -k SRR1284073.fastq.orig	./bzip2 -f -k -d SRR1284073.fastq.orig.bz2
BZIP2	365074789.00	2.37	5.75	0.92	3.75	./bzip2 -6 -f -k SRR1284073.fastq.orig	./bzip2 -f -k -d SRR1284073.fastq.orig.bz2
BZIP2	364857415.00	2.40	6.38	0.92	4.12	./bzip2 -7 -f -k SRR1284073.fastq.orig	./bzip2 -f -k -d SRR1284073.fastq.orig.bz2
BZIP2	364678329.00	2.42	7.13	0.93	4.50	./bzip2 -8 -f -k SRR1284073.fastq.orig	./bzip2 -f -k -d SRR1284073.fastq.orig.bz2
BZIP2	364521458.00	2.40	7.75	0.93	4.87	./bzip2 -9 -f -k SRR1284073.fastq.orig	./bzip2 -f -k -d SRR1284073.fastq.orig.bz2
BSC-m03	322972442.00	13.62	9710.40	12.65	9184.12	./bsc-m03 e SRR1284073.fastq.orig SRR1284073.fastq.bsc -b800000000	./bsc-m03 d SRR1284073.fastq.bsc SRR1284073.fastq.out
BSC-m03	324314849.00	13.72	4865.59	12.67	4862.59	./bsc-m03 e SRR1284073.fastq.orig SRR1284073.fastq.bsc -b400000000	./bsc-m03 d SRR1284073.fastq.bsc SRR1284073.fastq.out
BSC-m03	329116196.00	12.85	68.48	12.17	68.70	./bsc-m03 e SRR1284073.fastq.orig SRR1284073.fastq.bsc -b4096000	./bsc-m03 d SRR1284073.fastq.bsc SRR1284073.fastq.out
PAQ8L	319372332.00	176.12	39.63	176.27	39.50	./paq8l -1 SRR1284073.fastq	./paq8l -d SRR1284073.fastq.paq8l tmp_dir
JARVIS3	333506560.00	1.53	190.13	1.62	189.88	./JARVIS3.sh --level 1 --block 600MB --threads 1 --fastq --input SRR1284073.fastq	./JARVIS3.sh --decompress --threads 1 --fastq --input SRR1284073.fastq.tar

JARVIS3	333537280.00	1.60	190.13	1.60	190.00	./JARVIS3.sh --level 2 --block 600MB --threads 1 --fastq --input SRR1284073.fastq	./JARVIS3.sh --decompress --threads 1 --fastq --input SRR1284073.fastq.tar
JARVIS3	332165120.00	1.48	382.00	1.60	382.00	./JARVIS3.sh --level 3 --block 600MB --threads 1 --fastq --input SRR1284073.fastq	./JARVIS3.sh --decompress --threads 1 --fastq --input SRR1284073.fastq.tar
JARVIS3	330496000.00	1.48	1150.25	1.63	1149.87	./JARVIS3.sh --level 4 --block 600MB --threads 1 --fastq --input SRR1284073.fastq	./JARVIS3.sh --decompress --threads 1 --fastq --input SRR1284073.fastq.tar
JARVIS3	329687040.00	1.55	190.25	1.72	190.00	./JARVIS3.sh --level 5 --block 600MB --threads 1 --fastq --input SRR1284073.fastq	./JARVIS3.sh --decompress --threads 1 --fastq --input SRR1284073.fastq.tar
JARVIS3	326758400.00	2.72	382.13	2.87	381.88	./JARVIS3.sh --level 6 --block 600MB --threads 1 --fastq --input SRR1284073.fastq	./JARVIS3.sh --decompress --threads 1 --fastq --input SRR1284073.fastq.tar
JARVIS3	326471680.00	1.95	382.25	2.00	381.75	./JARVIS3.sh --level 7 --block 600MB --threads 1 --fastq --input SRR1284073.fastq	./JARVIS3.sh --decompress --threads 1 --fastq --input SRR1284073.fastq.tar
JARVIS3	324771840.00	1.98	1150.25	2.13	1149.75	./JARVIS3.sh --level 8 --block 600MB --threads 1 --fastq --input SRR1284073.fastq	./JARVIS3.sh --decompress --threads 1 --fastq --input SRR1284073.fastq.tar
JARVIS3	322539520.00	2.67	1150.00	2.83	1149.87	./JARVIS3.sh --level 9 --block 600MB --threads 1 --fastq --input SRR1284073.fastq	./JARVIS3.sh --decompress --threads 1 --fastq --input SRR1284073.fastq.tar
JARVIS3	326164480.00	2.63	190.00	2.77	190.00	./JARVIS3.sh --level 10 --block 600MB --threads 1 --fastq --input SRR1284073.fastq	./JARVIS3.sh --decompress --threads 1 --fastq --input SRR1284073.fastq.tar
JARVIS3	321904640.00	3.95	893.88	4.08	894.00	./JARVIS3.sh --level 11 --block 600MB --threads 1 --fastq --input SRR1284073.fastq	./JARVIS3.sh --decompress --threads 1 --fastq --input SRR1284073.fastq.tar
JARVIS3	323901440.00	4.27	382.13	4.45	382.00	./JARVIS3.sh --level 12 --block 600MB --threads 1 --fastq --input SRR1284073.fastq	./JARVIS3.sh --decompress --threads 1 --fastq --input SRR1284073.fastq.tar
JARVIS3	323409920.00	4.67	382.25	4.88	381.75	./JARVIS3.sh --level 13 --block 600MB --threads 1 --fastq --input SRR1284073.fastq	./JARVIS3.sh --decompress --threads 1 --fastq --input SRR1284073.fastq.tar
JARVIS3	326051840.00	2.65	318.37	2.87	318.25	./JARVIS3.sh --level 14 --block 600MB --threads 1 --fastq --input SRR1284073.fastq	./JARVIS3.sh --decompress --threads 1 --fastq --input SRR1284073.fastq.tar
JARVIS3	324689920.00	2.80	382.25	3.10	382.13	./JARVIS3.sh --level 15 --block 600MB --threads 1 --fastq --input SRR1284073.fastq	./JARVIS3.sh --decompress --threads 1 --fastq --input SRR1284073.fastq.tar
JARVIS3	324259840.00	3.13	318.13	3.37	318.13	./JARVIS3.sh --level 16 --block 600MB --threads 1 --fastq --input SRR1284073.fastq	./JARVIS3.sh --decompress --threads 1 --fastq --input SRR1284073.fastq.tar
JARVIS3	322119680.00	3.30	1150.25	3.55	1150.00	./JARVIS3.sh --level 17 --block 600MB --threads 1 --fastq --input SRR1284073.fastq	./JARVIS3.sh --decompress --threads 1 --fastq --input SRR1284073.fastq.tar
JARVIS3	321822720.00	3.33	1150.50	3.53	1150.13	./JARVIS3.sh --level 18 --block 600MB --threads 1 --fastq --input SRR1284073.fastq	./JARVIS3.sh --decompress --threads 1 --fastq --input SRR1284073.fastq.tar
JARVIS3	322252800.00	4.38	382.25	4.60	382.00	./JARVIS3.sh --level 19 --block 600MB --threads 1 --fastq --input SRR1284073.fastq	./JARVIS3.sh --decompress --threads 1 --fastq --input SRR1284073.fastq.tar
JARVIS3	320614400.00	6.97	446.50	7.17	446.25	./JARVIS3.sh --level 20 --block 600MB --threads 1 --fastq --input SRR1284073.fastq	./JARVIS3.sh --decompress --threads 1 --fastq --input SRR1284073.fastq.tar
JARVIS3	319600640.00	8.63	510.13	8.90	510.00	./JARVIS3.sh --level 21 --block 600MB --threads 1 --fastq --input SRR1284073.fastq	./JARVIS3.sh --decompress --threads 1 --fastq --input SRR1284073.fastq.tar
JARVIS3	319610880.00	8.70	510.37	8.97	510.00	./JARVIS3.sh --level 22 --block 600MB --threads 1 --fastq --input SRR1284073.fastq	./JARVIS3.sh --decompress --threads 1 --fastq --input SRR1284073.fastq.tar
JARVIS3	319467520.00	8.88	574.50	9.08	573.63	./JARVIS3.sh --level 23 --block 600MB --threads 1 --fastq --input SRR1284073.fastq	./JARVIS3.sh --decompress --threads 1 --fastq --input SRR1284073.fastq.tar
JARVIS3	319139840.00	3.70	4222.37	3.75	4222.00	./JARVIS3.sh --level 24 --block 600MB --threads 1 --fastq --input SRR1284073.fastq	./JARVIS3.sh --decompress --threads 1 --fastq --input SRR1284073.fastq.tar
JARVIS3	315996160.00	9.90	4222.00	10.35	4222.13	./JARVIS3.sh --level 25 --block 600MB --threads 1 --fastq --input SRR1284073.fastq	./JARVIS3.sh --decompress --threads 1 --fastq --input SRR1284073.fastq.tar
JARVIS3	313098240.00	12.90	4222.25	13.28	4222.25	./JARVIS3.sh --level 26 --block 600MB --threads 1 --fastq --input SRR1284073.fastq	./JARVIS3.sh --decompress --threads 1 --fastq --input SRR1284073.fastq.tar
SPRING	330301440.00	2.32	600.07	1.37	580.77	./spring -c -t 1 --long -i SRR1284073.fastq -o SRR1284073.fastq.spring	./spring -d -t 1 --long -i SRR1284073.fastq.spring -o SRR1284073.fastq.out
LEON	381218800.00	4.23	2400.96	1.38	1218.25	./leon -nb-cores 1 -lossless -c -file SRR1284073.fastq	./leon -nb-cores 1 -d -file SRR1284073.fastq.leon

Table S8: Results obtained when compressing the SRR8858470.

**Compressor**	**Compressed data size (bytes)**	**Compression time (minutes)**	**Compression memory (MB)**	**Decompression time (minutes)**	**Decompression memory (MB)**	**Compression options**	**Decompression options**
JARVIS2	1517670400.00	111.00	7002.87	110.00	7003.00	./JARVIS2.sh --fastq --level 1 --block 600MB --threads 1 --fastq --input SRR8858470.fastq	./JARVIS2.sh --decompress --threads 1 --fastq --input SRR8858470.fastq.tar
JARVIS2	1517670400.00	109.15	7003.62	109.55	7003.00	./JARVIS2.sh --fastq --level 2 --block 600MB --threads 1 --fastq --input SRR8858470.fastq	./JARVIS2.sh --decompress --threads 1 --fastq --input SRR8858470.fastq.tar
JARVIS2	1517670400.00	109.42	7003.25	109.88	7003.13	./JARVIS2.sh --fastq --level 3 --block 600MB --threads 1 --fastq --input SRR8858470.fastq	./JARVIS2.sh --decompress --threads 1 --fastq --input SRR8858470.fastq.tar
JARVIS2	1517670400.00	109.53	7002.99	109.90	7002.88	./JARVIS2.sh --fastq --level 7 --block 600MB --threads 1 --fastq --input SRR8858470.fastq	./JARVIS2.sh --decompress --threads 1 --fastq --input SRR8858470.fastq.tar
NAF	1660136593.00	0.28	16.25	0.63	493.37	./ennaf --temp-dir tmp/ --fastq --level 1 -o NAF-OUT.naf SRR8858470.fastq	./unnaf -o NAD-D-OUT.naf NAF-OUT.naf
NAF	1668071178.00	0.40	20.12	0.52	492.62	./ennaf --temp-dir tmp/ --fastq --level 2 -o NAF-OUT.naf SRR8858470.fastq	./unnaf -o NAD-D-OUT.naf NAF-OUT.naf
NAF	1697992077.00	0.55	28.12	0.62	518.13	./ennaf --temp-dir tmp/ --fastq --level 3 -o NAF-OUT.naf SRR8858470.fastq	./unnaf -o NAD-D-OUT.naf NAF-OUT.naf
NAF	1726257013.00	0.65	35.62	0.67	538.38	./ennaf --temp-dir tmp/ --fastq --level 4 -o NAF-OUT.naf SRR8858470.fastq	./unnaf -o NAD-D-OUT.naf NAF-OUT.naf
NAF	1706952247.00	0.77	41.75	0.58	521.63	./ennaf --temp-dir tmp/ --fastq --level 5 -o NAF-OUT.naf SRR8858470.fastq	./unnaf -o NAD-D-OUT.naf NAF-OUT.naf
NAF	1679037400.00	2.08	175.88	0.70	514.89	./ennaf --temp-dir tmp/ --fastq --level 11 -o NAF-OUT.naf SRR8858470.fastq	./unnaf -o NAD-D-OUT.naf NAF-OUT.naf
NAF	1573217463.00	27.55	529.75	0.62	481.77	./ennaf --temp-dir tmp/ --fastq --level 19 -o NAF-OUT.naf SRR8858470.fastq	./unnaf -o NAD-D-OUT.naf NAF-OUT.naf
NAF	1557349732.00	45.62	4178.37	0.72	700.64	./ennaf --temp-dir tmp/ --fastq --level 22 -o NAF-OUT.naf SRR8858470.fastq	./unnaf -o NAD-D-OUT.naf NAF-OUT.naf
LZMA	1791855800.00	12.72	10.75	3.00	3.38	./xz -1 -f -k SRR8858470.fastq.orig	./xz -f -k -d SRR8858470.fastq.orig.xz
LZMA	1783661392.00	15.75	18.25	2.90	4.25	./xz -2 -f -k SRR8858470.fastq.orig	./xz -f -k -d SRR8858470.fastq.orig.xz
LZMA	1779962508.00	20.65	33.38	2.83	6.38	./xz -3 -f -k SRR8858470.fastq.orig	./xz -f -k -d SRR8858470.fastq.orig.xz
LZMA	1584613000.00	61.13	49.50	2.63	6.38	./xz -4 -f -k SRR8858470.fastq.orig	./xz -f -k -d SRR8858470.fastq.orig.xz
LZMA	1556966388.00	78.00	95.37	2.63	10.25	./xz -5 -f -k SRR8858470.fastq.orig	./xz -f -k -d SRR8858470.fastq.orig.xz
LZMA	1551084768.00	84.62	95.37	2.62	10.37	./xz -6 -f -k SRR8858470.fastq.orig	./xz -f -k -d SRR8858470.fastq.orig.xz
LZMA	1547547464.00	92.40	187.37	2.65	18.25	./xz -7 -f -k SRR8858470.fastq.orig	./xz -f -k -d SRR8858470.fastq.orig.xz
LZMA	1543233448.00	100.72	371.38	2.65	34.37	./xz -8 -f -k SRR8858470.fastq.orig	./xz -f -k -d SRR8858470.fastq.orig.xz
LZMA	1539084444.00	110.10	675.38	2.63	66.37	./xz -9 -f -k SRR8858470.fastq.orig	./xz -f -k -d SRR8858470.fastq.orig.xz
BZIP2	1741130924.00	8.93	2.50	3.83	1.87	./bzip2 -1 -f -k SRR8858470.fastq.orig	./bzip2 -f -k -d SRR8858470.fastq.orig.bz2
BZIP2	1736736215.00	8.87	3.13	3.98	2.13	./bzip2 -2 -f -k SRR8858470.fastq.orig	./bzip2 -f -k -d SRR8858470.fastq.orig.bz2
BZIP2	1732200190.00	8.92	3.75	3.98	2.50	./bzip2 -3 -f -k SRR8858470.fastq.orig	./bzip2 -f -k -d SRR8858470.fastq.orig.bz2
BZIP2	1728450675.00	8.93	4.37	4.02	2.88	./bzip2 -4 -f -k SRR8858470.fastq.orig	./bzip2 -f -k -d SRR8858470.fastq.orig.bz2
BZIP2	1725364972.00	9.10	5.12	4.00	3.25	./bzip2 -5 -f -k SRR8858470.fastq.orig	./bzip2 -f -k -d SRR8858470.fastq.orig.bz2
BZIP2	1722896213.00	9.10	5.75	4.05	3.75	./bzip2 -6 -f -k SRR8858470.fastq.orig	./bzip2 -f -k -d SRR8858470.fastq.orig.bz2
BZIP2	1720735974.00	9.27	6.38	4.07	4.00	./bzip2 -7 -f -k SRR8858470.fastq.orig	./bzip2 -f -k -d SRR8858470.fastq.orig.bz2
BZIP2	1718925161.00	9.23	7.13	4.07	4.50	./bzip2 -8 -f -k SRR8858470.fastq.orig	./bzip2 -f -k -d SRR8858470.fastq.orig.bz2
BZIP2	1717304716.00	9.37	7.75	4.07	4.87	./bzip2 -9 -f -k SRR8858470.fastq.orig	./bzip2 -f -k -d SRR8858470.fastq.orig.bz2
BSC-m03	1457512875.00	61.62	9404.94	58.00	9404.95	./bsc-m03 e SRR8858470.fastq.orig SRR8858470.fastq.bsc -b800000000	./bsc-m03 d SRR8858470.fastq.bsc SRR8858470.fastq.out
BSC-m03	1466710767.00	61.22	4748.17	57.82	4748.18	./bsc-m03 e SRR8858470.fastq.orig SRR8858470.fastq.bsc -b400000000	./bsc-m03 d SRR8858470.fastq.bsc SRR8858470.fastq.out
BSC-m03	1527038209.00	59.63	63.89	56.90	64.46	./bsc-m03 e SRR8858470.fastq.orig SRR8858470.fastq.bsc -b4096000	./bsc-m03 d SRR8858470.fastq.bsc SRR8858470.fastq.out
PAQ8L	1446797270.00	1024.27	40.00	1023.70	40.00	./paq8l -1 SRR8858470.fastq	./paq8l -d SRR8858470.fastq.paq8l tmp_dir
JARVIS3	1519749120.00	16.13	210.37	17.58	210.12	./JARVIS3.sh --level 1 --block 600MB --threads 1 --fastq --input SRR8858470.fastq	./JARVIS3.sh --decompress --threads 1 --fastq --input SRR8858470.fastq.tar

JARVIS3	1517209600.00	16.18	210.37	17.68	210.00	./JARVIS3.sh --level 2 --block 600MB --threads 1 --fastq --input SRR8858470.fastq	./JARVIS3.sh --decompress --threads 1 --fastq --input SRR8858470.fastq.tar
JARVIS3	1501665280.00	16.22	402.37	17.77	402.12	./JARVIS3.sh --level 3 --block 600MB --threads 1 --fastq --input SRR8858470.fastq	./JARVIS3.sh --decompress --threads 1 --fastq --input SRR8858470.fastq.tar
JARVIS3	1487902720.00	16.22	1170.25	17.70	1170.12	./JARVIS3.sh --level 4 --block 600MB --threads 1 --fastq --input SRR8858470.fastq	./JARVIS3.sh --decompress --threads 1 --fastq --input SRR8858470.fastq.tar
JARVIS3	1495951360.00	16.47	210.12	17.68	210.12	./JARVIS3.sh --level 5 --block 600MB --threads 1 --fastq --input SRR8858470.fastq	./JARVIS3.sh --decompress --threads 1 --fastq --input SRR8858470.fastq.tar
JARVIS3	1473853440.00	16.25	402.37	17.85	402.12	./JARVIS3.sh --level 6 --block 600MB --threads 1 --fastq --input SRR8858470.fastq	./JARVIS3.sh --decompress --threads 1 --fastq --input SRR8858470.fastq.tar
JARVIS3	1471037440.00	16.30	402.37	17.70	402.00	./JARVIS3.sh --level 7 --block 600MB --threads 1 --fastq --input SRR8858470.fastq	./JARVIS3.sh --decompress --threads 1 --fastq --input SRR8858470.fastq.tar
JARVIS3	1461442560.00	16.22	1170.37	17.87	1170.12	./JARVIS3.sh --level 8 --block 600MB --threads 1 --fastq --input SRR8858470.fastq	./JARVIS3.sh --decompress --threads 1 --fastq --input SRR8858470.fastq.tar
JARVIS3	1452963840.00	16.35	1170.25	17.82	1170.12	./JARVIS3.sh --level 9 --block 600MB --threads 1 --fastq --input SRR8858470.fastq	./JARVIS3.sh --decompress --threads 1 --fastq --input SRR8858470.fastq.tar
JARVIS3	1474048000.00	16.27	210.37	17.73	210.12	./JARVIS3.sh --level 10 --block 600MB --threads 1 --fastq --input SRR8858470.fastq	./JARVIS3.sh --decompress --threads 1 --fastq --input SRR8858470.fastq.tar
JARVIS3	1450301440.00	16.50	914.25	18.02	914.00	./JARVIS3.sh --level 11 --block 600MB --threads 1 --fastq --input SRR8858470.fastq	./JARVIS3.sh --decompress --threads 1 --fastq --input SRR8858470.fastq.tar
JARVIS3	1459169280.00	17.87	402.37	18.10	402.12	./JARVIS3.sh --level 12 --block 600MB --threads 1 --fastq --input SRR8858470.fastq	./JARVIS3.sh --decompress --threads 1 --fastq --input SRR8858470.fastq.tar
JARVIS3	1457868800.00	19.68	402.37	19.45	402.12	./JARVIS3.sh --level 13 --block 600MB --threads 1 --fastq --input SRR8858470.fastq	./JARVIS3.sh --decompress --threads 1 --fastq --input SRR8858470.fastq.tar
JARVIS3	1477345280.00	16.22	338.50	17.93	338.25	./JARVIS3.sh --level 14 --block 600MB --threads 1 --fastq --input SRR8858470.fastq	./JARVIS3.sh --decompress --threads 1 --fastq --input SRR8858470.fastq.tar
JARVIS3	1463951360.00	16.57	402.25	18.12	402.25	./JARVIS3.sh --level 15 --block 600MB --threads 1 --fastq --input SRR8858470.fastq	./JARVIS3.sh --decompress --threads 1 --fastq --input SRR8858470.fastq.tar
JARVIS3	1460776960.00	16.53	338.50	17.82	338.12	./JARVIS3.sh --level 16 --block 600MB --threads 1 --fastq --input SRR8858470.fastq	./JARVIS3.sh --decompress --threads 1 --fastq --input SRR8858470.fastq.tar
JARVIS3	1443850240.00	16.33	1170.37	17.93	1170.25	./JARVIS3.sh --level 17 --block 600MB --threads 1 --fastq --input SRR8858470.fastq	./JARVIS3.sh --decompress --threads 1 --fastq --input SRR8858470.fastq.tar
JARVIS3	1443594240.00	16.68	1170.50	17.98	1170.37	./JARVIS3.sh --level 18 --block 600MB --threads 1 --fastq --input SRR8858470.fastq	./JARVIS3.sh --decompress --threads 1 --fastq --input SRR8858470.fastq.tar
JARVIS3	1446645760.00	19.55	402.37	18.27	402.37	./JARVIS3.sh --level 19 --block 600MB --threads 1 --fastq --input SRR8858470.fastq	./JARVIS3.sh --decompress --threads 1 --fastq --input SRR8858470.fastq.tar
JARVIS3	1438423040.00	28.35	466.37	28.52	466.12	./JARVIS3.sh --level 20 --block 600MB --threads 1 --fastq --input SRR8858470.fastq	./JARVIS3.sh --decompress --threads 1 --fastq --input SRR8858470.fastq.tar
JARVIS3	1433139200.00	39.02	530.37	39.28	530.25	./JARVIS3.sh --level 21 --block 600MB --threads 1 --fastq --input SRR8858470.fastq	./JARVIS3.sh --decompress --threads 1 --fastq --input SRR8858470.fastq.tar
JARVIS3	1432514560.00	43.55	530.50	43.97	530.12	./JARVIS3.sh --level 22 --block 600MB --threads 1 --fastq --input SRR8858470.fastq	./JARVIS3.sh --decompress --threads 1 --fastq --input SRR8858470.fastq.tar
JARVIS3	1431695360.00	47.52	594.75	47.90	594.37	./JARVIS3.sh --level 23 --block 600MB --threads 1 --fastq --input SRR8858470.fastq	./JARVIS3.sh --decompress --threads 1 --fastq --input SRR8858470.fastq.tar
JARVIS3	1432135680.00	16.50	4241.50	17.83	4241.38	./JARVIS3.sh --level 24 --block 600MB --threads 1 --fastq --input SRR8858470.fastq	./JARVIS3.sh --decompress --threads 1 --fastq --input SRR8858470.fastq.tar
JARVIS3	1419735040.00	41.82	4241.62	42.35	4241.75	./JARVIS3.sh --level 25 --block 600MB --threads 1 --fastq --input SRR8858470.fastq	./JARVIS3.sh --decompress --threads 1 --fastq --input SRR8858470.fastq.tar
JARVIS3	1415751680.00	55.02	4241.88	55.30	4241.62	./JARVIS3.sh --level 26 --block 600MB --threads 1 --fastq --input SRR8858470.fastq	./JARVIS3.sh --decompress --threads 1 --fastq --input SRR8858470.fastq.tar
SPRING	1504153600.00	9.35	831.78	6.07	907.33	./spring -c -t 1 --long -i SRR8858470.fastq -o SRR8858470.fastq.spring	./spring -d -t 1 --long -i SRR8858470.fastq.spring -o SRR8858470.fastq.out
LEON	1702159776.00	28.60	4232.36	10.20	4685.93	./leon -nb-cores 1 -lossless -c -file SRR8858470.fastq	./leon -nb-cores 1 -d -file SRR8858470.fastq.leon

Table S9: Results obtained when compressing the SRR9046049.

**Compressor**	**Compressed data size (bytes)**	**Compression time (minutes)**	**Compression memory (MB)**	**Decompression time (minutes)**	**Decompression memory (MB)**	**Compression options**	**Decompression options**
JARVIS2	840120320.00	58.20	1247.38	58.63	1247.13	./JARVIS2.sh --fastq --level 1 --block 600MB --threads 1 --fastq --input SRR9046049.fastq	./JARVIS2.sh --decompress --threads 1 --fastq --input SRR9046049.fastq.tar
JARVIS2	840120320.00	57.97	1247.25	58.42	1247.38	./JARVIS2.sh --fastq --level 2 --block 600MB --threads 1 --fastq --input SRR9046049.fastq	./JARVIS2.sh --decompress --threads 1 --fastq --input SRR9046049.fastq.tar
JARVIS2	840120320.00	58.22	1247.25	58.55	1247.13	./JARVIS2.sh --fastq --level 3 --block 600MB --threads 1 --fastq --input SRR9046049.fastq	./JARVIS2.sh --decompress --threads 1 --fastq --input SRR9046049.fastq.tar
JARVIS2	840120320.00	57.95	1247.38	58.23	1247.25	./JARVIS2.sh --fastq --level 7 --block 600MB --threads 1 --fastq --input SRR9046049.fastq	./JARVIS2.sh --decompress --threads 1 --fastq --input SRR9046049.fastq.tar
NAF	898415640.00	0.18	17.75	0.38	325.00	./ennaf --temp-dir tmp/ --fastq --level 1 -o NAF-OUT.naf SRR9046049.fastq	./unnaf -o NAD-D-OUT.naf NAF-OUT.naf
NAF	922177520.00	0.30	22.38	0.37	324.25	./ennaf --temp-dir tmp/ --fastq --level 2 -o NAF-OUT.naf SRR9046049.fastq	./unnaf -o NAD-D-OUT.naf NAF-OUT.naf
NAF	986487275.00	0.42	30.88	0.42	345.99	./ennaf --temp-dir tmp/ --fastq --level 3 -o NAF-OUT.naf SRR9046049.fastq	./unnaf -o NAD-D-OUT.naf NAF-OUT.naf
NAF	1015385949.00	0.47	38.38	0.45	360.11	./ennaf --temp-dir tmp/ --fastq --level 4 -o NAF-OUT.naf SRR9046049.fastq	./unnaf -o NAD-D-OUT.naf NAF-OUT.naf
NAF	980448344.00	0.53	44.25	0.40	349.61	./ennaf --temp-dir tmp/ --fastq --level 5 -o NAF-OUT.naf SRR9046049.fastq	./unnaf -o NAD-D-OUT.naf NAF-OUT.naf
NAF	974598571.00	1.87	180.25	0.43	352.61	./ennaf --temp-dir tmp/ --fastq --level 11 -o NAF-OUT.naf SRR9046049.fastq	./unnaf -o NAD-D-OUT.naf NAF-OUT.naf
NAF	907595962.00	26.88	534.38	0.42	330.14	./ennaf --temp-dir tmp/ --fastq --level 19 -o NAF-OUT.naf SRR9046049.fastq	./unnaf -o NAD-D-OUT.naf NAF-OUT.naf
NAF	892129726.00	46.08	4183.26	0.48	561.39	./ennaf --temp-dir tmp/ --fastq --level 22 -o NAF-OUT.naf SRR9046049.fastq	./unnaf -o NAD-D-OUT.naf NAF-OUT.naf
LZMA	1082012952.00	8.18	10.75	1.68	3.38	./xz -1 -f -k SRR9046049.fastq.orig	./xz -f -k -d SRR9046049.fastq.orig.xz
LZMA	1062955136.00	11.60	18.25	1.60	4.25	./xz -2 -f -k SRR9046049.fastq.orig	./xz -f -k -d SRR9046049.fastq.orig.xz
LZMA	1053197732.00	20.17	32.13	1.53	6.38	./xz -3 -f -k SRR9046049.fastq.orig	./xz -f -k -d SRR9046049.fastq.orig.xz
LZMA	945127092.00	48.45	48.25	1.78	6.38	./xz -4 -f -k SRR9046049.fastq.orig	./xz -f -k -d SRR9046049.fastq.orig.xz
LZMA	945691632.00	58.67	89.12	1.72	10.37	./xz -5 -f -k SRR9046049.fastq.orig	./xz -f -k -d SRR9046049.fastq.orig.xz
LZMA	936944424.00	59.20	89.12	1.78	10.37	./xz -6 -f -k SRR9046049.fastq.orig	./xz -f -k -d SRR9046049.fastq.orig.xz
LZMA	937963464.00	67.02	165.87	1.75	18.37	./xz -7 -f -k SRR9046049.fastq.orig	./xz -f -k -d SRR9046049.fastq.orig.xz
LZMA	937580756.00	74.15	318.50	1.73	34.25	./xz -8 -f -k SRR9046049.fastq.orig	./xz -f -k -d SRR9046049.fastq.orig.xz
LZMA	935953508.00	82.62	622.62	1.68	66.37	./xz -9 -f -k SRR9046049.fastq.orig	./xz -f -k -d SRR9046049.fastq.orig.xz
BZIP2	963297739.00	6.00	2.38	2.18	1.87	./bzip2 -1 -f -k SRR9046049.fastq.orig	./bzip2 -f -k -d SRR9046049.fastq.orig.bz2
BZIP2	959987081.00	6.02	3.13	2.30	2.13	./bzip2 -2 -f -k SRR9046049.fastq.orig	./bzip2 -f -k -d SRR9046049.fastq.orig.bz2
BZIP2	958995623.00	6.02	3.75	2.33	2.50	./bzip2 -3 -f -k SRR9046049.fastq.orig	./bzip2 -f -k -d SRR9046049.fastq.orig.bz2
BZIP2	958570190.00	6.12	4.37	2.33	2.88	./bzip2 -4 -f -k SRR9046049.fastq.orig	./bzip2 -f -k -d SRR9046049.fastq.orig.bz2
BZIP2	958312340.00	6.22	5.12	2.37	3.25	./bzip2 -5 -f -k SRR9046049.fastq.orig	./bzip2 -f -k -d SRR9046049.fastq.orig.bz2
BZIP2	958125969.00	6.33	5.75	2.37	3.63	./bzip2 -6 -f -k SRR9046049.fastq.orig	./bzip2 -f -k -d SRR9046049.fastq.orig.bz2
BZIP2	957997828.00	6.42	6.38	2.37	4.12	./bzip2 -7 -f -k SRR9046049.fastq.orig	./bzip2 -f -k -d SRR9046049.fastq.orig.bz2
BZIP2	957916228.00	6.45	7.13	2.37	4.50	./bzip2 -8 -f -k SRR9046049.fastq.orig	./bzip2 -f -k -d SRR9046049.fastq.orig.bz2
BZIP2	957884429.00	6.50	7.75	2.38	4.87	./bzip2 -9 -f -k SRR9046049.fastq.orig	./bzip2 -f -k -d SRR9046049.fastq.orig.bz2
BSC-m03	858320508.00	35.53	9595.48	33.00	9595.11	./bsc-m03 e SRR9046049.fastq.orig SRR9046049.fastq.bsc -b800000000	./bsc-m03 d SRR9046049.fastq.bsc SRR9046049.fastq.out
BSC-m03	862444715.00	35.57	4807.49	32.83	4807.17	./bsc-m03 e SRR9046049.fastq.orig SRR9046049.fastq.bsc -b400000000	./bsc-m03 d SRR9046049.fastq.bsc SRR9046049.fastq.out
BSC-m03	871556822.00	33.52	63.82	31.90	63.97	./bsc-m03 e SRR9046049.fastq.orig SRR9046049.fastq.bsc -b4096000	./bsc-m03 d SRR9046049.fastq.bsc SRR9046049.fastq.out
PAQ8L	845822676.00	515.82	40.00	515.88	40.00	./paq8l -1 SRR9046049.fastq	./paq8l -d SRR9046049.fastq.paq8l tmp_dir
JARVIS3	890767360.00	3.47	210.12	4.25	210.12	./JARVIS3.sh --level 1 --block 600MB --threads 1 --fastq --input SRR9046049.fastq	./JARVIS3.sh --decompress --threads 1 --fastq --input SRR9046049.fastq.tar

JARVIS3	890777600.00	3.92	210.12	4.02	210.00	./JARVIS3.sh --level 2 --block 600MB --threads 1 --fastq --input SRR9046049.fastq	./JARVIS3.sh --decompress --threads 1 --fastq --input SRR9046049.fastq.tar
JARVIS3	889088000.00	3.85	402.25	4.23	402.00	./JARVIS3.sh --level 3 --block 600MB --threads 1 --fastq --input SRR9046049.fastq	./JARVIS3.sh --decompress --threads 1 --fastq --input SRR9046049.fastq.tar
JARVIS3	886046720.00	3.88	1170.37	3.98	1170.12	./JARVIS3.sh --level 4 --block 600MB --threads 1 --fastq --input SRR9046049.fastq	./JARVIS3.sh --decompress --threads 1 --fastq --input SRR9046049.fastq.tar
JARVIS3	880035840.00	4.00	210.25	3.97	210.00	./JARVIS3.sh --level 5 --block 600MB --threads 1 --fastq --input SRR9046049.fastq	./JARVIS3.sh --decompress --threads 1 --fastq --input SRR9046049.fastq.tar
JARVIS3	874229760.00	7.42	402.37	7.22	402.00	./JARVIS3.sh --level 6 --block 600MB --threads 1 --fastq --input SRR9046049.fastq	./JARVIS3.sh --decompress --threads 1 --fastq --input SRR9046049.fastq.tar
JARVIS3	873113600.00	4.82	402.37	4.95	401.87	./JARVIS3.sh --level 7 --block 600MB --threads 1 --fastq --input SRR9046049.fastq	./JARVIS3.sh --decompress --threads 1 --fastq --input SRR9046049.fastq.tar
JARVIS3	869857280.00	5.10	1170.25	5.22	1169.88	./JARVIS3.sh --level 8 --block 600MB --threads 1 --fastq --input SRR9046049.fastq	./JARVIS3.sh --decompress --threads 1 --fastq --input SRR9046049.fastq.tar
JARVIS3	862791680.00	6.75	1170.25	6.83	1170.00	./JARVIS3.sh --level 9 --block 600MB --threads 1 --fastq --input SRR9046049.fastq	./JARVIS3.sh --decompress --threads 1 --fastq --input SRR9046049.fastq.tar
JARVIS3	868556800.00	6.53	210.25	6.68	210.00	./JARVIS3.sh --level 10 --block 600MB --threads 1 --fastq --input SRR9046049.fastq	./JARVIS3.sh --decompress --threads 1 --fastq --input SRR9046049.fastq.tar
JARVIS3	862474240.00	9.87	914.37	9.95	914.00	./JARVIS3.sh --level 11 --block 600MB --threads 1 --fastq --input SRR9046049.fastq	./JARVIS3.sh --decompress --threads 1 --fastq --input SRR9046049.fastq.tar
JARVIS3	865587200.00	10.73	402.50	10.93	402.00	./JARVIS3.sh --level 12 --block 600MB --threads 1 --fastq --input SRR9046049.fastq	./JARVIS3.sh --decompress --threads 1 --fastq --input SRR9046049.fastq.tar
JARVIS3	864348160.00	11.52	402.50	11.78	402.00	./JARVIS3.sh --level 13 --block 600MB --threads 1 --fastq --input SRR9046049.fastq	./JARVIS3.sh --decompress --threads 1 --fastq --input SRR9046049.fastq.tar
JARVIS3	864276480.00	6.70	338.25	7.02	338.12	./JARVIS3.sh --level 14 --block 600MB --threads 1 --fastq --input SRR9046049.fastq	./JARVIS3.sh --decompress --threads 1 --fastq --input SRR9046049.fastq.tar
JARVIS3	860579840.00	7.17	402.37	7.62	402.25	./JARVIS3.sh --level 15 --block 600MB --threads 1 --fastq --input SRR9046049.fastq	./JARVIS3.sh --decompress --threads 1 --fastq --input SRR9046049.fastq.tar
JARVIS3	859064320.00	7.92	338.37	8.20	338.25	./JARVIS3.sh --level 16 --block 600MB --threads 1 --fastq --input SRR9046049.fastq	./JARVIS3.sh --decompress --threads 1 --fastq --input SRR9046049.fastq.tar
JARVIS3	855470080.00	8.70	1170.50	8.85	1170.37	./JARVIS3.sh --level 17 --block 600MB --threads 1 --fastq --input SRR9046049.fastq	./JARVIS3.sh --decompress --threads 1 --fastq --input SRR9046049.fastq.tar
JARVIS3	854773760.00	8.53	1170.37	8.68	1170.25	./JARVIS3.sh --level 18 --block 600MB --threads 1 --fastq --input SRR9046049.fastq	./JARVIS3.sh --decompress --threads 1 --fastq --input SRR9046049.fastq.tar
JARVIS3	853401600.00	11.35	402.37	11.08	402.12	./JARVIS3.sh --level 19 --block 600MB --threads 1 --fastq --input SRR9046049.fastq	./JARVIS3.sh --decompress --threads 1 --fastq --input SRR9046049.fastq.tar
JARVIS3	849797120.00	17.50	466.50	17.65	466.00	./JARVIS3.sh --level 20 --block 600MB --threads 1 --fastq --input SRR9046049.fastq	./JARVIS3.sh --decompress --threads 1 --fastq --input SRR9046049.fastq.tar
JARVIS3	847134720.00	22.15	530.37	22.42	530.12	./JARVIS3.sh --level 21 --block 600MB --threads 1 --fastq --input SRR9046049.fastq	./JARVIS3.sh --decompress --threads 1 --fastq --input SRR9046049.fastq.tar
JARVIS3	847134720.00	22.08	530.37	22.35	529.87	./JARVIS3.sh --level 22 --block 600MB --threads 1 --fastq --input SRR9046049.fastq	./JARVIS3.sh --decompress --threads 1 --fastq --input SRR9046049.fastq.tar
JARVIS3	847257600.00	22.43	594.37	22.92	594.12	./JARVIS3.sh --level 23 --block 600MB --threads 1 --fastq --input SRR9046049.fastq	./JARVIS3.sh --decompress --threads 1 --fastq --input SRR9046049.fastq.tar
JARVIS3	846643200.00	9.00	4242.63	9.15	4242.12	./JARVIS3.sh --level 24 --block 600MB --threads 1 --fastq --input SRR9046049.fastq	./JARVIS3.sh --decompress --threads 1 --fastq --input SRR9046049.fastq.tar
JARVIS3	839608320.00	24.92	4242.25	25.72	4242.25	./JARVIS3.sh --level 25 --block 600MB --threads 1 --fastq --input SRR9046049.fastq	./JARVIS3.sh --decompress --threads 1 --fastq --input SRR9046049.fastq.tar
JARVIS3	831150080.00	33.42	4242.50	34.17	4242.25	./JARVIS3.sh --level 26 --block 600MB --threads 1 --fastq --input SRR9046049.fastq	./JARVIS3.sh --decompress --threads 1 --fastq --input SRR9046049.fastq.tar
SPRING	879421440.00	6.20	555.69	3.55	595.43	./spring -c -t 1 --long -i SRR9046049.fastq -o SRR9046049.fastq.spring	./spring -d -t 1 --long -i SRR9046049.fastq.spring -o SRR9046049.fastq.out
LEON	996477576.00	11.22	2807.18	4.58	1139.29	./leon -nb-cores 1 -lossless -c -file SRR9046049.fastq	./leon -nb-cores 1 -d -file SRR9046049.fastq.leon

Table S10: Results obtained when compressing the Viral genomes database.

**Compressor**	**Compressed data size (bytes)**	**Compression time (minutes)**	**Compression memory (MB)**	**Decompression time (minutes)**	**Decompression memory (MB)**	**Compression options**	**Decompression options**
AGC	47473515.00	0.88	1763.67	0.02	286.46	./agc create -t 1 VDB.fa	./agc getcol -t 1 AGC-OUT.agc
JARVIS2	3880960.00	31.30	642.62	31.48	642.62	./JARVIS2.sh --fasta --level 1 --block 600MB --threads 1 --fasta --input VDB.fa	./JARVIS2.sh --decompress --threads 1 --fasta --input VDB.fa.tar
JARVIS2	3880960.00	31.37	642.75	31.40	642.37	./JARVIS2.sh --fasta --level 2 --block 600MB --threads 1 --fasta --input VDB.fa	./JARVIS2.sh --decompress --threads 1 --fasta --input VDB.fa.tar
JARVIS2	3880960.00	31.35	642.62	31.40	642.75	./JARVIS2.sh --fasta --level 3 --block 600MB --threads 1 --fasta --input VDB.fa	./JARVIS2.sh --decompress --threads 1 --fasta --input VDB.fa.tar
JARVIS2	3880960.00	31.37	642.50	31.40	642.62	./JARVIS2.sh --fasta --level 7 --block 600MB --threads 1 --fasta --input VDB.fa	./JARVIS2.sh --decompress --threads 1 --fasta --input VDB.fa.tar
NAF	13204349.00	0.00	9.50	0.02	5.75	./ennaf --temp-dir tmp/ --dna --level 1 -o NAF-OUT.naf VDB.fa	./unnaf -o NAD-D-OUT.naf NAF-OUT.naf
NAF	9354065.00	0.00	11.50	0.02	6.25	./ennaf --temp-dir tmp/ --dna --level 2 -o NAF-OUT.naf VDB.fa	./unnaf -o NAD-D-OUT.naf NAF-OUT.naf
NAF	8962481.00	0.00	15.25	0.02	7.13	./ennaf --temp-dir tmp/ --dna --level 3 -o NAF-OUT.naf VDB.fa	./unnaf -o NAD-D-OUT.naf NAF-OUT.naf
NAF	9103186.00	0.00	21.37	0.02	7.13	./ennaf --temp-dir tmp/ --dna --level 4 -o NAF-OUT.naf VDB.fa	./unnaf -o NAD-D-OUT.naf NAF-OUT.naf
NAF	8784652.00	0.00	26.50	0.02	7.13	./ennaf --temp-dir tmp/ --dna --level 5 -o NAF-OUT.naf VDB.fa	./unnaf -o NAD-D-OUT.naf NAF-OUT.naf
NAF	7656035.00	0.03	133.37	0.02	9.12	./ennaf --temp-dir tmp/ --dna --level 11 -o NAF-OUT.naf VDB.fa	./unnaf -o NAD-D-OUT.naf NAF-OUT.naf
NAF	5989280.00	0.43	420.37	0.02	13.25	./ennaf --temp-dir tmp/ --dna --level 19 -o NAF-OUT.naf VDB.fa	./unnaf -o NAD-D-OUT.naf NAF-OUT.naf
NAF	5912553.00	0.85	3339.12	0.02	91.25	./ennaf --temp-dir tmp/ --dna --level 22 -o NAF-OUT.naf VDB.fa	./unnaf -o NAD-D-OUT.naf NAF-OUT.naf
LZMA	35600496.00	0.32	10.63	0.05	3.25	./xz -1 -f -k VDB.fa.orig	./xz -f -k -d VDB.fa.orig.xz
LZMA	31412272.00	0.38	18.13	0.05	4.25	./xz -2 -f -k VDB.fa.orig	./xz -f -k -d VDB.fa.orig.xz
LZMA	29372608.00	0.52	33.00	0.05	6.13	./xz -3 -f -k VDB.fa.orig	./xz -f -k -d VDB.fa.orig.xz
LZMA	8486796.00	1.33	49.38	0.02	6.25	./xz -4 -f -k VDB.fa.orig	./xz -f -k -d VDB.fa.orig.xz
LZMA	7300764.00	1.78	94.62	0.02	10.25	./xz -5 -f -k VDB.fa.orig	./xz -f -k -d VDB.fa.orig.xz
LZMA	6948296.00	2.37	94.62	0.02	10.25	./xz -6 -f -k VDB.fa.orig	./xz -f -k -d VDB.fa.orig.xz
LZMA	6715708.00	2.47	180.50	0.02	18.25	./xz -7 -f -k VDB.fa.orig	./xz -f -k -d VDB.fa.orig.xz
LZMA	6637968.00	2.55	343.00	0.02	34.25	./xz -8 -f -k VDB.fa.orig	./xz -f -k -d VDB.fa.orig.xz
LZMA	6614020.00	2.62	647.00	0.02	66.25	./xz -9 -f -k VDB.fa.orig	./xz -f -k -d VDB.fa.orig.xz
BZIP2	43194145.00	0.47	2.38	0.13	1.87	./bzip2 -1 -f -k VDB.fa.orig	./bzip2 -f -k -d VDB.fa.orig.bz2
BZIP2	41012131.00	0.48	3.13	0.13	2.25	./bzip2 -2 -f -k VDB.fa.orig	./bzip2 -f -k -d VDB.fa.orig.bz2
BZIP2	38643269.00	0.50	3.75	0.12	2.63	./bzip2 -3 -f -k VDB.fa.orig	./bzip2 -f -k -d VDB.fa.orig.bz2
BZIP2	35946164.00	0.52	4.75	0.12	3.00	./bzip2 -4 -f -k VDB.fa.orig	./bzip2 -f -k -d VDB.fa.orig.bz2
BZIP2	34533780.00	0.55	5.50	0.12	3.38	./bzip2 -5 -f -k VDB.fa.orig	./bzip2 -f -k -d VDB.fa.orig.bz2
BZIP2	32433806.00	0.55	6.25	0.12	3.75	./bzip2 -6 -f -k VDB.fa.orig	./bzip2 -f -k -d VDB.fa.orig.bz2
BZIP2	31503201.00	0.57	7.00	0.12	4.12	./bzip2 -7 -f -k VDB.fa.orig	./bzip2 -f -k -d VDB.fa.orig.bz2
BZIP2	29847415.00	0.58	7.88	0.12	4.50	./bzip2 -8 -f -k VDB.fa.orig	./bzip2 -f -k -d VDB.fa.orig.bz2
BZIP2	29249226.00	0.60	8.62	0.12	4.87	./bzip2 -9 -f -k VDB.fa.orig	./bzip2 -f -k -d VDB.fa.orig.bz2
BSC-m03	12160066.00	1.75	2026.16	1.52	1861.25	./bsc-m03 e VDB.fa.orig VDB.fa.bsc -b800000000	./bsc-m03 d VDB.fa.bsc VDB.fa.out
BSC-m03	12160066.00	1.75	2026.28	1.52	1861.38	./bsc-m03 e VDB.fa.orig VDB.fa.bsc -b400000000	./bsc-m03 d VDB.fa.bsc VDB.fa.out
BSC-m03	15337570.00	1.58	62.43	1.37	62.88	./bsc-m03 e VDB.fa.orig VDB.fa.bsc -b4096000	./bsc-m03 d VDB.fa.bsc VDB.fa.out
MFCompress	7127604.00	0.42	540.00	0.43	538.38	./MFCompressC -v -1 -p 1 -t 1 -o MFC-OUT.mfc VDB.fa	./MFCompressD -o MFC-OUT.d MFC-OUT.mfc

MFCompress	7077956.00	0.45	540.50	0.58	538.63	./MFCompressC -v -2 -p 1 -t 1 -o MFC-OUT.mfc VDB.fa	./MFCompressD -o MFC-OUT.d MFC-OUT.mfc
MFCompress	6547856.00	0.87	2348.00	0.75	2346.37	./MFCompressC -v -3 -p 1 -t 1 -o MFC-OUT.mfc VDB.fa	./MFCompressD -o MFC-OUT.d MFC-OUT.mfc
PAQ8L	22959073.00	31.35	39.37	31.18	39.25	./paq8l -1 VDB.fa	./paq8l -d VDB.fa.paq8l tmp_dir
JARVIS3	5529600.00	0.27	110.37	0.25	110.00	./JARVIS3.sh --level 1 --block 600MB --threads 1 --fasta --input VDB.fa	./JARVIS3.sh --decompress --threads 1 --fasta --input VDB.fa.tar
JARVIS3	5406720.00	0.27	110.25	0.27	110.00	./JARVIS3.sh --level 2 --block 600MB --threads 1 --fasta --input VDB.fa	./JARVIS3.sh --decompress --threads 1 --fasta --input VDB.fa.tar
JARVIS3	5365760.00	0.27	302.37	0.27	302.13	./JARVIS3.sh --level 3 --block 600MB --threads 1 --fasta --input VDB.fa	./JARVIS3.sh --decompress --threads 1 --fasta --input VDB.fa.tar
JARVIS3	5314560.00	0.30	1070.25	0.28	1070.00	./JARVIS3.sh --level 4 --block 600MB --threads 1 --fasta --input VDB.fa	./JARVIS3.sh --decompress --threads 1 --fasta --input VDB.fa.tar
JARVIS3	5181440.00	0.30	110.37	0.30	110.13	./JARVIS3.sh --level 5 --block 600MB --threads 1 --fasta --input VDB.fa	./JARVIS3.sh --decompress --threads 1 --fasta --input VDB.fa.tar
JARVIS3	4567040.00	0.53	302.37	0.53	302.13	./JARVIS3.sh --level 6 --block 600MB --threads 1 --fasta --input VDB.fa	./JARVIS3.sh --decompress --threads 1 --fasta --input VDB.fa.tar
JARVIS3	5038080.00	0.35	302.37	0.35	302.13	./JARVIS3.sh --level 7 --block 600MB --threads 1 --fasta --input VDB.fa	./JARVIS3.sh --decompress --threads 1 --fasta --input VDB.fa.tar
JARVIS3	5048320.00	0.38	1070.38	0.37	1069.88	./JARVIS3.sh --level 8 --block 600MB --threads 1 --fasta --input VDB.fa	./JARVIS3.sh --decompress --threads 1 --fasta --input VDB.fa.tar
JARVIS3	4894720.00	0.43	1070.25	0.43	1070.00	./JARVIS3.sh --level 9 --block 600MB --threads 1 --fasta --input VDB.fa	./JARVIS3.sh --decompress --threads 1 --fasta --input VDB.fa.tar
JARVIS3	4945920.00	0.45	110.13	0.43	110.13	./JARVIS3.sh --level 10 --block 600MB --threads 1 --fasta --input VDB.fa	./JARVIS3.sh --decompress --threads 1 --fasta --input VDB.fa.tar
JARVIS3	4382720.00	0.70	814.50	0.68	814.00	./JARVIS3.sh --level 11 --block 600MB --threads 1 --fasta --input VDB.fa	./JARVIS3.sh --decompress --threads 1 --fasta --input VDB.fa.tar
JARVIS3	4249600.00	0.73	302.37	0.75	302.00	./JARVIS3.sh --level 12 --block 600MB --threads 1 --fasta --input VDB.fa	./JARVIS3.sh --decompress --threads 1 --fasta --input VDB.fa.tar
JARVIS3	4239360.00	0.82	302.25	0.82	302.13	./JARVIS3.sh --level 13 --block 600MB --threads 1 --fasta --input VDB.fa	./JARVIS3.sh --decompress --threads 1 --fasta --input VDB.fa.tar
JARVIS3	5079040.00	0.62	238.37	0.65	238.13	./JARVIS3.sh --level 14 --block 600MB --threads 1 --fasta --input VDB.fa	./JARVIS3.sh --decompress --threads 1 --fasta --input VDB.fa.tar
JARVIS3	4648960.00	0.65	302.25	0.68	302.25	./JARVIS3.sh --level 15 --block 600MB --threads 1 --fasta --input VDB.fa	./JARVIS3.sh --decompress --threads 1 --fasta --input VDB.fa.tar
JARVIS3	4567040.00	0.73	238.50	0.70	238.25	./JARVIS3.sh --level 16 --block 600MB --threads 1 --fasta --input VDB.fa	./JARVIS3.sh --decompress --threads 1 --fasta --input VDB.fa.tar
JARVIS3	4464640.00	0.73	1070.38	0.73	1070.25	./JARVIS3.sh --level 17 --block 600MB --threads 1 --fasta --input VDB.fa	./JARVIS3.sh --decompress --threads 1 --fasta --input VDB.fa.tar
JARVIS3	4454400.00	0.73	1070.38	0.75	1070.25	./JARVIS3.sh --level 18 --block 600MB --threads 1 --fasta --input VDB.fa	./JARVIS3.sh --decompress --threads 1 --fasta --input VDB.fa.tar
JARVIS3	4218880.00	0.87	302.62	0.85	302.25	./JARVIS3.sh --level 19 --block 600MB --threads 1 --fasta --input VDB.fa	./JARVIS3.sh --decompress --threads 1 --fasta --input VDB.fa.tar
JARVIS3	4014080.00	1.28	366.50	1.30	366.13	./JARVIS3.sh --level 20 --block 600MB --threads 1 --fasta --input VDB.fa	./JARVIS3.sh --decompress --threads 1 --fasta --input VDB.fa.tar
JARVIS3	3921920.00	2.28	430.50	2.32	430.13	./JARVIS3.sh --level 21 --block 600MB --threads 1 --fasta --input VDB.fa	./JARVIS3.sh --decompress --threads 1 --fasta --input VDB.fa.tar
JARVIS3	3911680.00	3.90	430.62	3.90	430.25	./JARVIS3.sh --level 22 --block 600MB --threads 1 --fasta --input VDB.fa	./JARVIS3.sh --decompress --threads 1 --fasta --input VDB.fa.tar
JARVIS3	3891200.00	6.92	494.50	6.90	494.25	./JARVIS3.sh --level 23 --block 600MB --threads 1 --fasta --input VDB.fa	./JARVIS3.sh --decompress --threads 1 --fasta --input VDB.fa.tar
JARVIS3	4802560.00	0.72	4083.37	0.73	4083.00	./JARVIS3.sh --level 24 --block 600MB --threads 1 --fasta --input VDB.fa	./JARVIS3.sh --decompress --threads 1 --fasta --input VDB.fa.tar
JARVIS3	4485120.00	3.00	4083.50	3.07	4083.25	./JARVIS3.sh --level 25 --block 600MB --threads 1 --fasta --input VDB.fa	./JARVIS3.sh --decompress --threads 1 --fasta --input VDB.fa.tar
JARVIS3	4311040.00	3.87	4083.25	3.92	4083.37	./JARVIS3.sh --level 26 --block 600MB --threads 1 --fasta --input VDB.fa	./JARVIS3.sh --decompress --threads 1 --fasta --input VDB.fa.tar

1.4 Results of the Benchmark - plots

1.4.1 Compression size / execution time

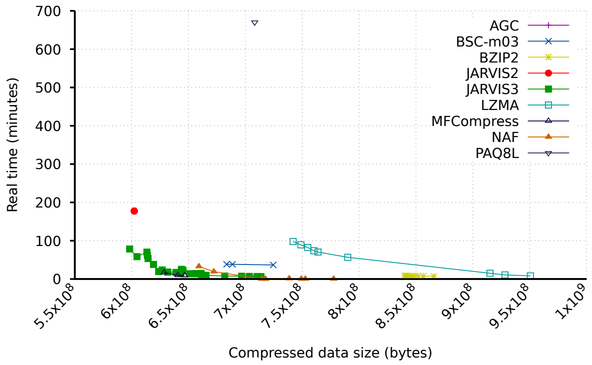

(a) Human T2T genome

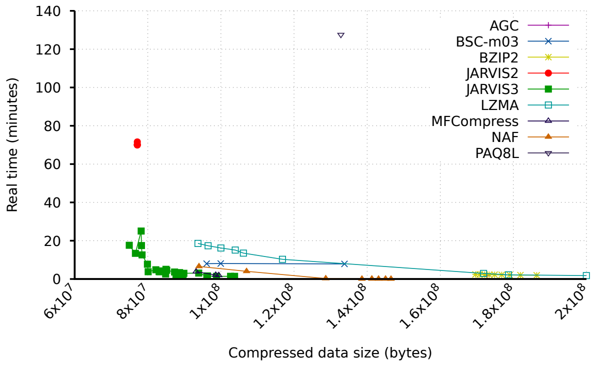

(b) Cassava genome [8]

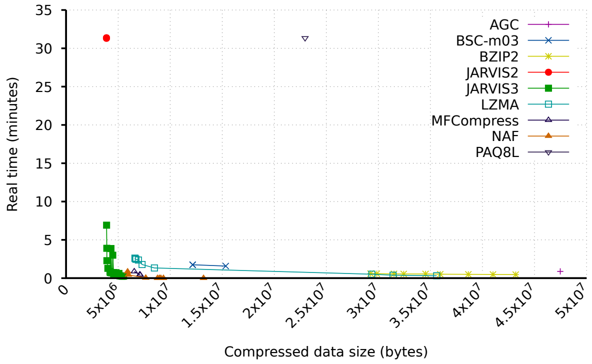

(c) Viral genomes database

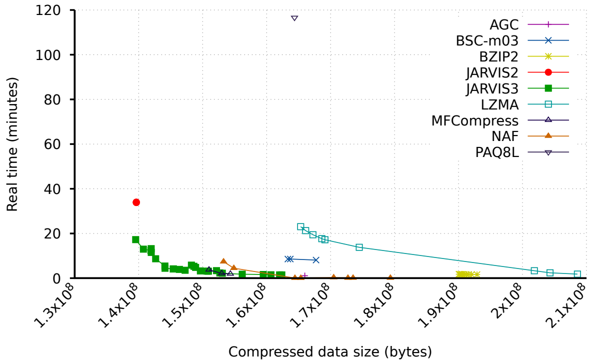

(d) DNA Corpus [9]

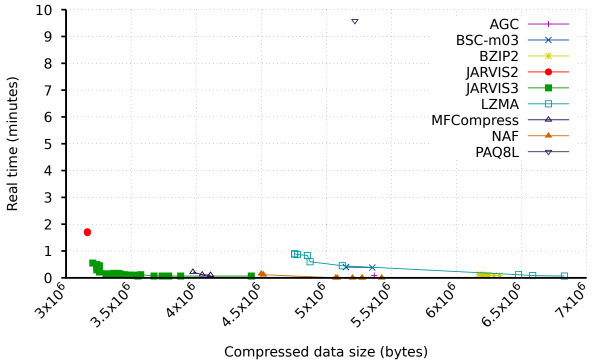

(e) Human Y chromosome (highly-repetitive)

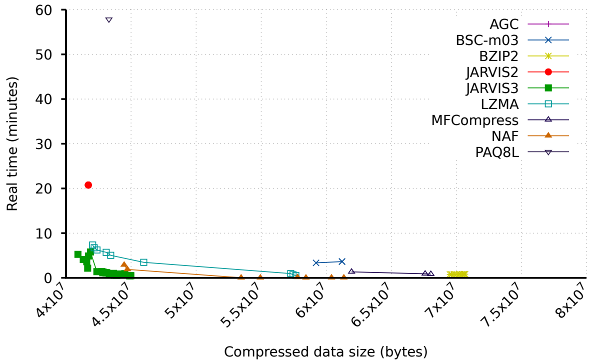

(f) UCSC hg38 7way knownCanonical-exonNuc [10]

Figure S1: Compression Benchmark depicting the compression time and size for nine compression tools over six datasets represented in FASTA format. The result for the ACG was not represented in Figure 1f as it represented an outlier.

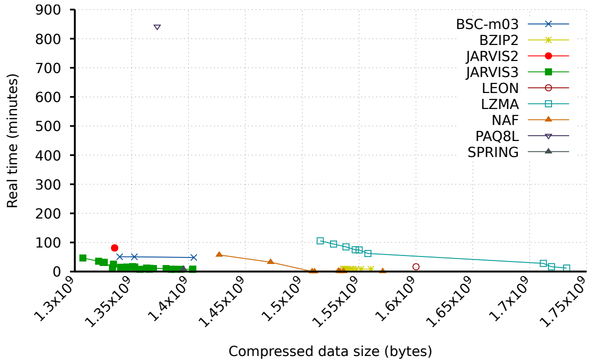

(a) Sample ERR3307082

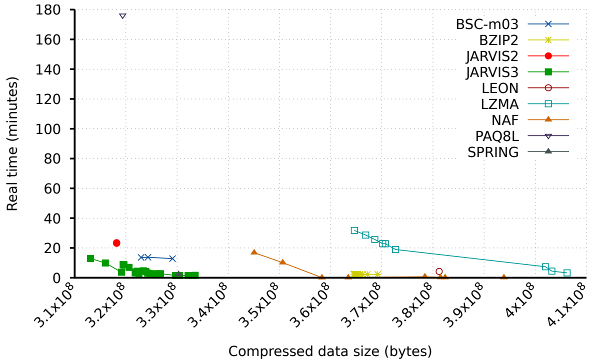

(b) Sample SRR1284073

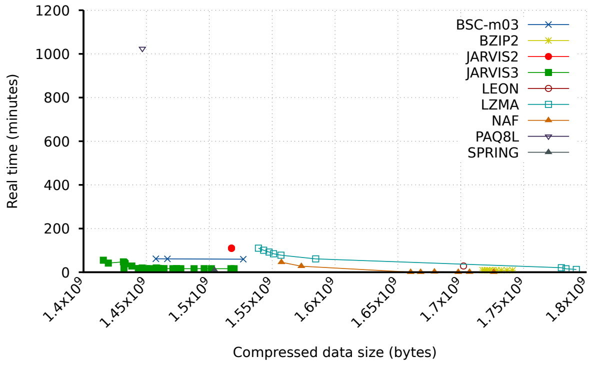

(c) Sample SRR8858470

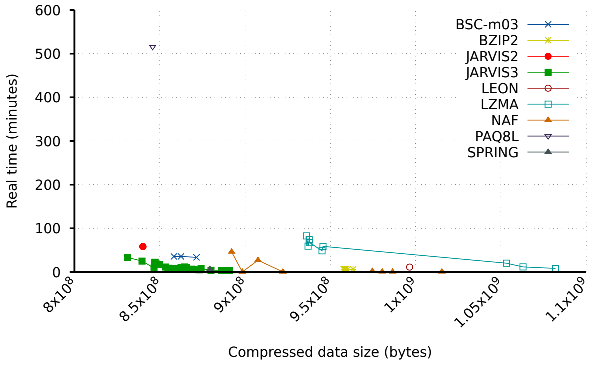

(d) Sample SRR9046049

Figure S2: Compression Benchmark depicting the compression time and size for nine compression tools over four datasets represented in FASTQ format.

1.4.2 Compression size / execution time (logarithmic scale)

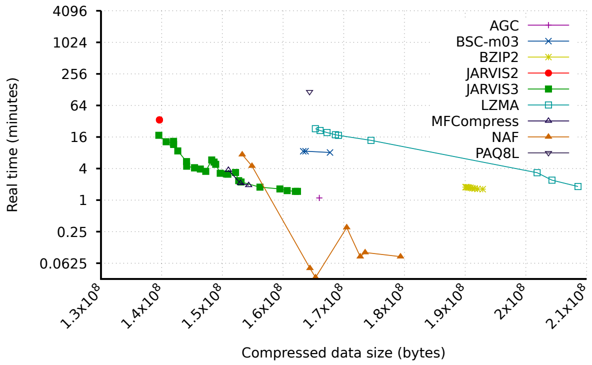

(a) DNA Corpus [9]

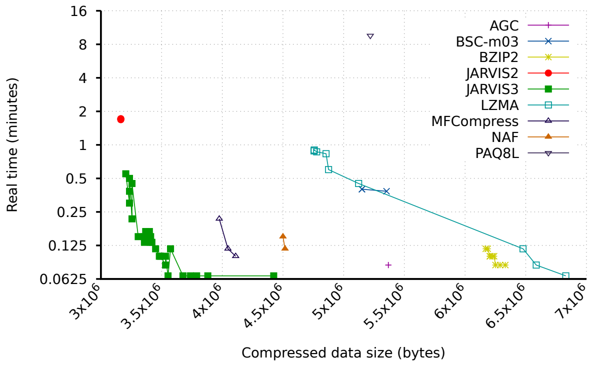

(b) Human Y chromosome (highly-repetitive)

Figure S3: Compression Benchmark depicting the compression time and size for nine compression tools over two datasets represented in FASTA format. The y-axis is represented as a logarithmic scale of base 2.

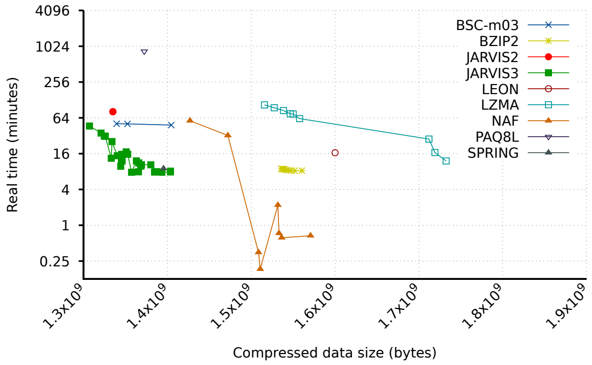

(a) Sample ERR3307082

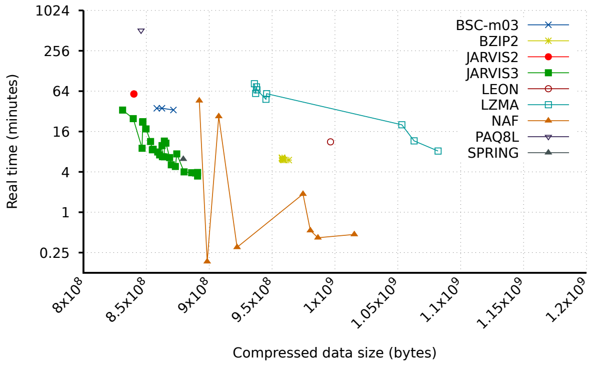

(b) Sample SRR9046049

Figure S4: Compression Benchmark depicting the compression time and size for nine compression tools over two datasets represented in FASTQ format. The y-axis is represented as a logarithmic scale of base 2.

1.4.3 Decompression size / execution time

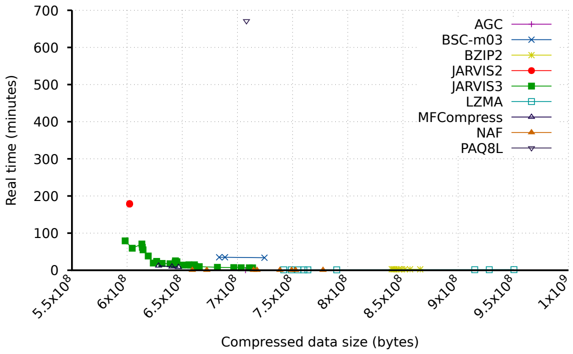

(a) Human T2T genome

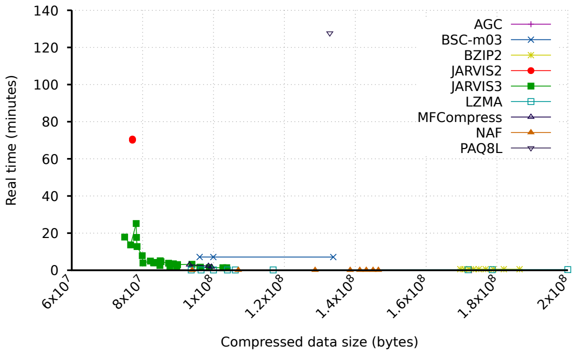

(b) Cassava genome [8]

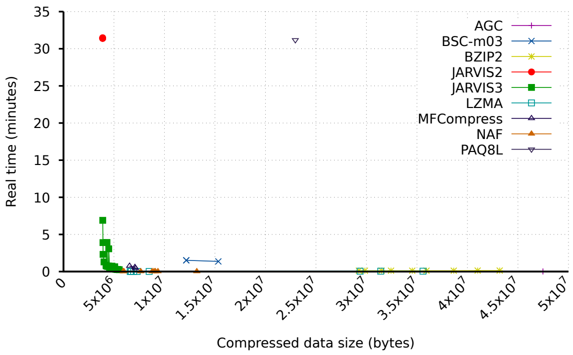

(c) Viral genomes database

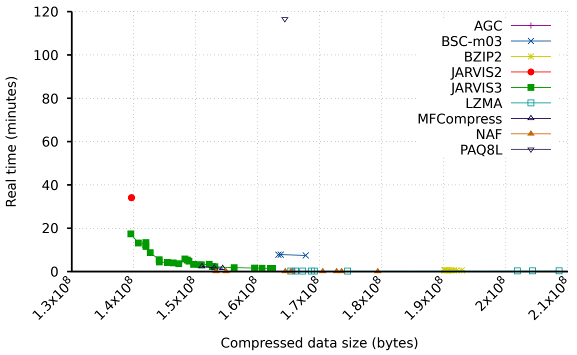

(d) DNA Corpus [9]

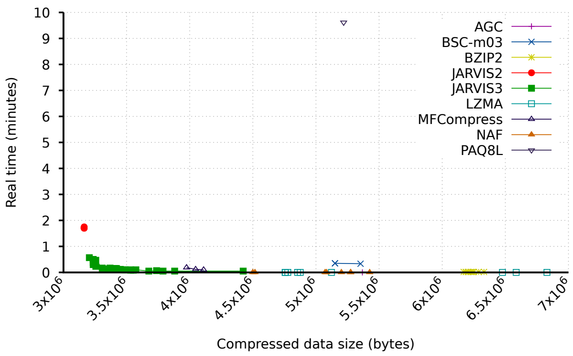

(e) Human Y chromosome (highly-repetitive)

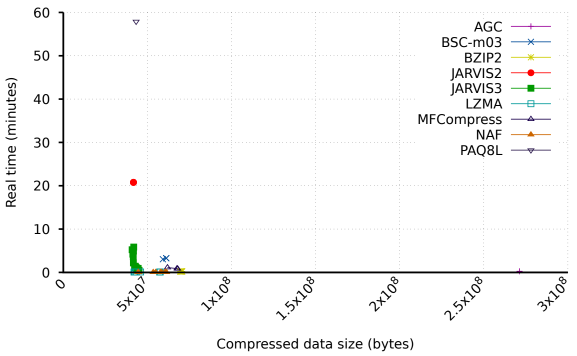

(f) UCSC hg38 7way knownCanonical-exonNuc [10]

Figure S5: Compression Benchmark depicting the decompression time and size for nine compression tools over six datasets represented in FASTA format. The result for the ACG was not represented in Figure 5f as it represented an outlier.

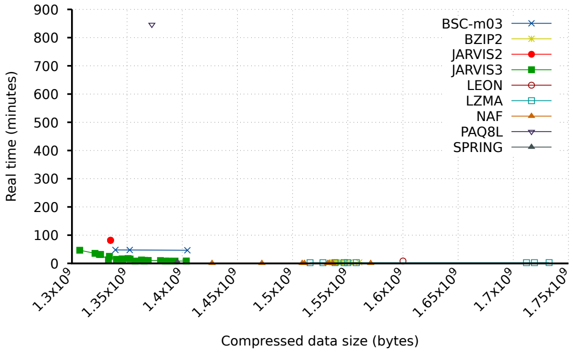

(a) Sample ERR3307082

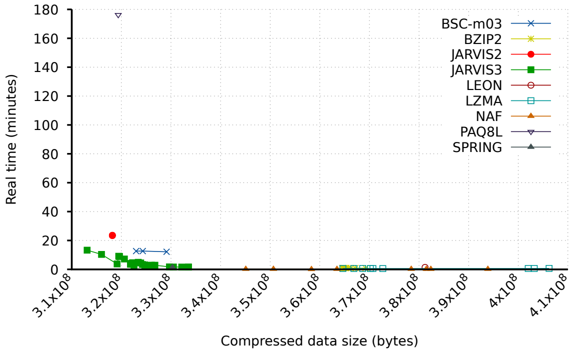

(b) Sample SRR1284073

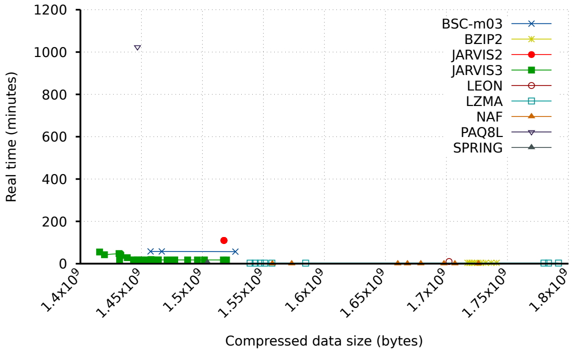

(c) Sample SRR8858470

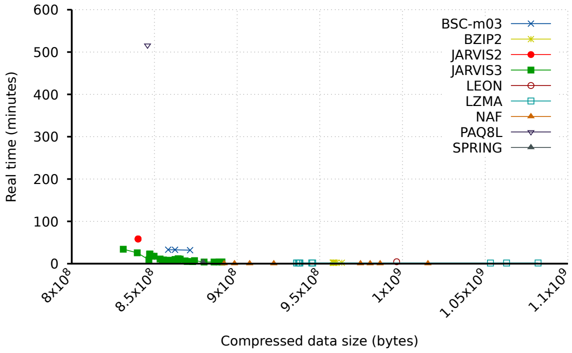

(d) Sample SRR9046049

Figure S6: Compression Benchmark depicting the decompression time and size for nine compression tools over four datasets represented in FASTQ format.

1.4.4 Decompression size / execution time (logarithmic scale)

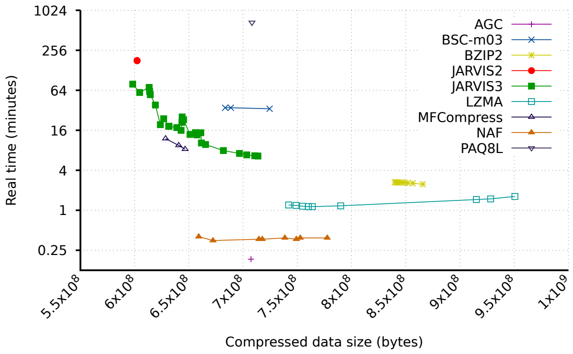

(a) Human T2T genome

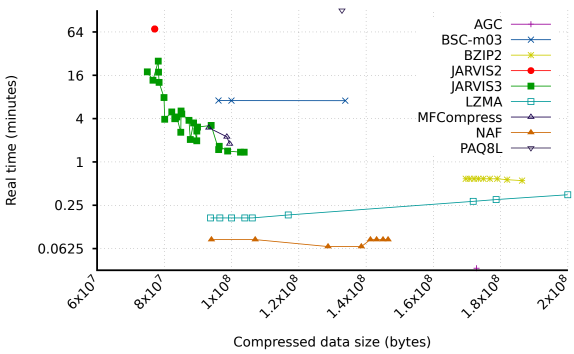

(b) Cassava genome [8]

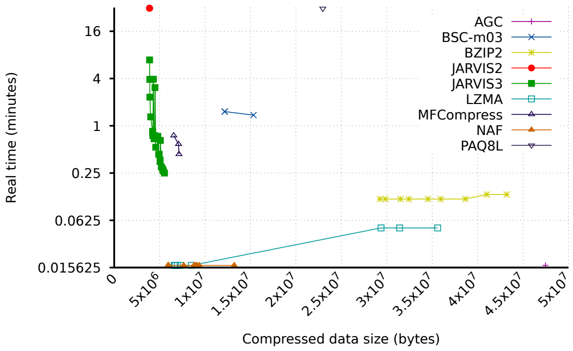

(c) Viral genomes database

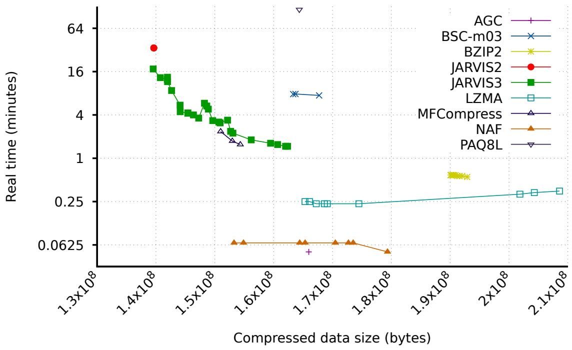

(d) DNA Corpus [9]

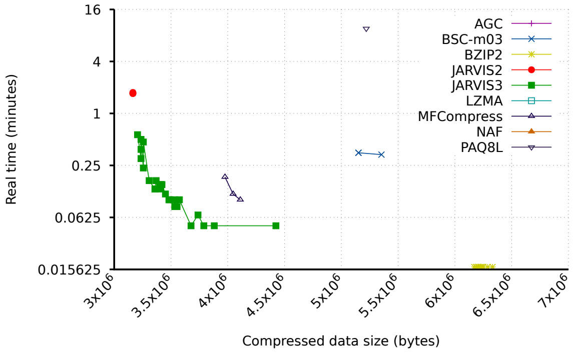

(e) Human Y chromosome (highly-repetitive)

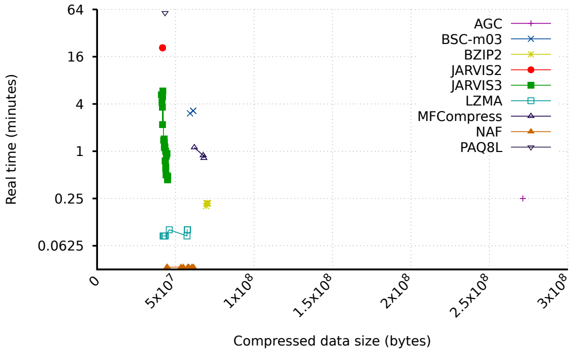

(f) UCSC hg38 7way knownCanonical-exonNuc [10]

Figure S7: Compression Benchmark depicting the decompression time and size for nine compression tools over six datasets represented in FASTA format. The result for the ACG was not represented in Figure 7f as it represented an outlier. The y-axis is represented as a logarithmic scale of base 2.

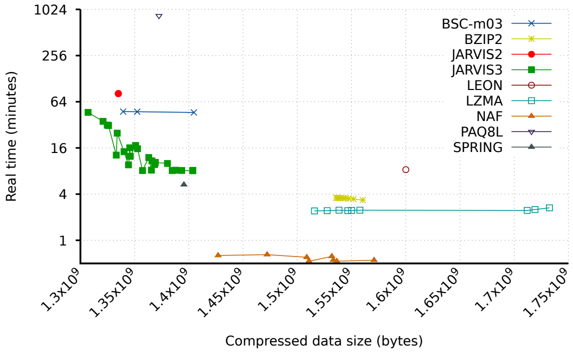

(a) Sample ERR3307082

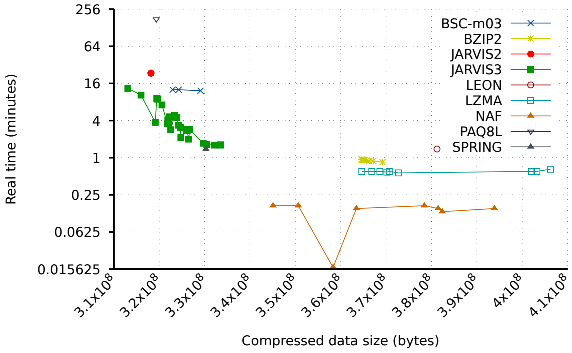

(b) Sample SRR1284073

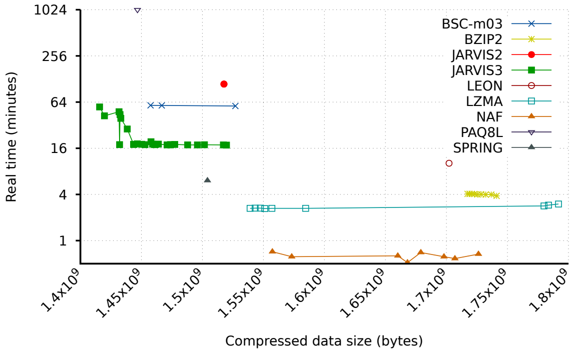

(c) Sample SRR8858470

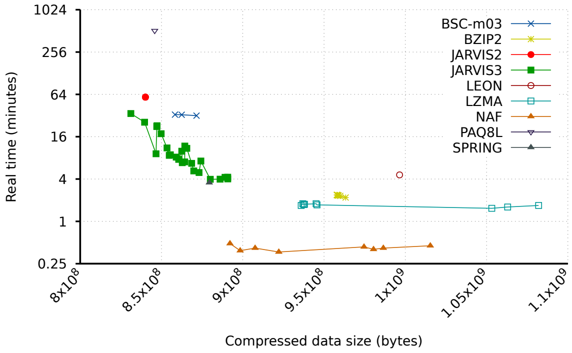

(d) Sample SRR9046049

Figure S8: Compression Benchmark depicting the decompression time and size for nine compression tools over four datasets represented in FASTQ format. The y-axis is represented as a logarithmic scale of base 2.

1.4.5 Compression size / memory usage (logarithmic scale)

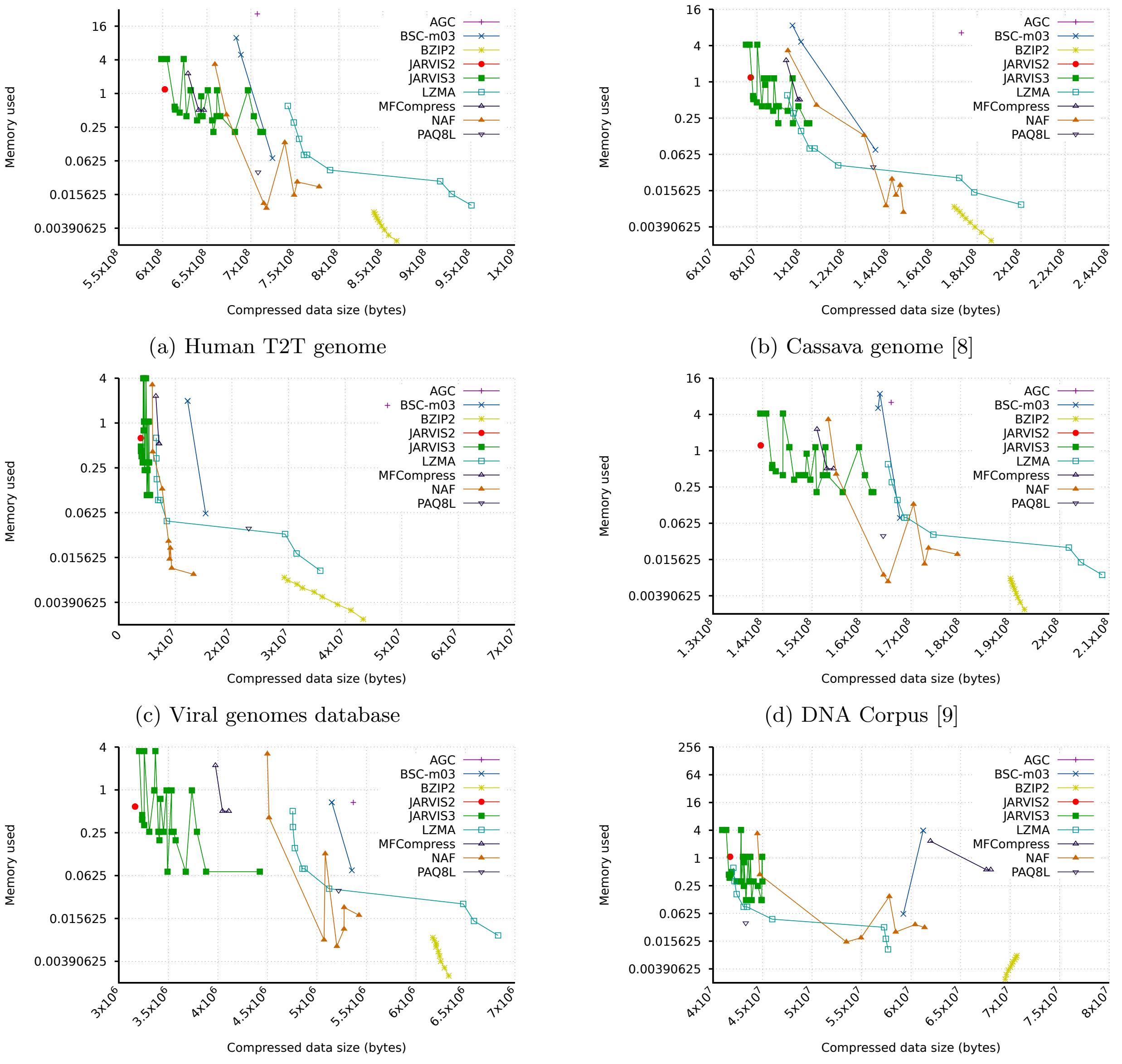

(a) Human T2T genome

(b) Cassava genome [8]

(c) Viral genomes database

(d) DNA Corpus [9]

(e) Human Y chromosome (highly-repetitive)

(f) UCSC hg38 7way knownCanonical-exonNuc [10]

Figure S9: Compression Benchmark depicting the compression memory and size for nine compression tools over six datasets represented in FASTA format. The result for the ACG was not represented in Figure 9f as it represented an outlier. The y-axis is represented as a logarithmic scale of base 2.

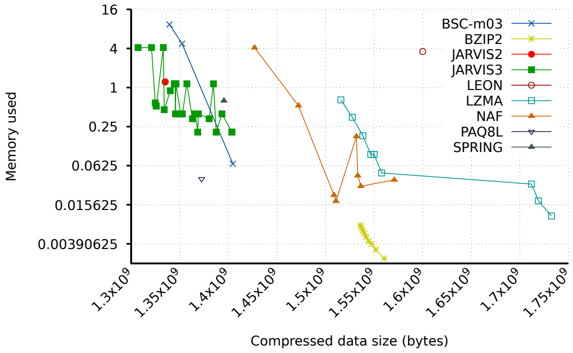

(a) Sample ERR3307082

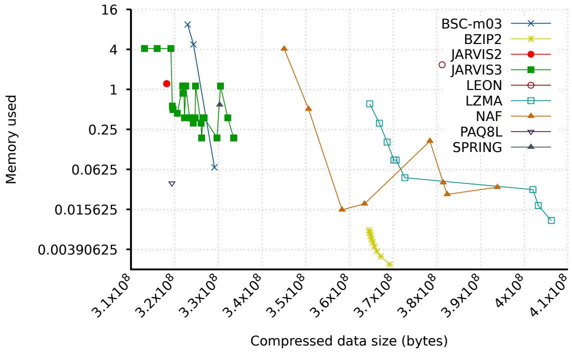

(b) Sample SRR1284073

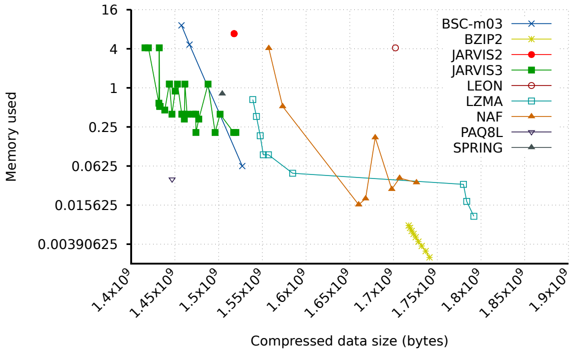

(c) Sample SRR8858470

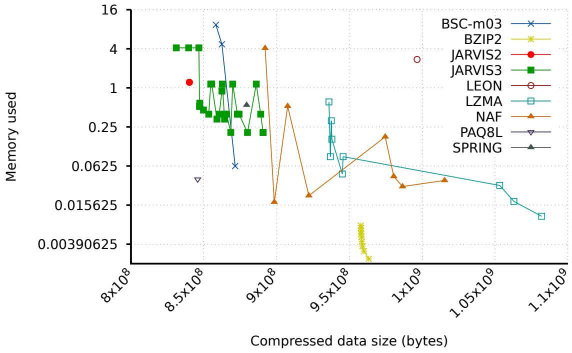

(d) Sample SRR9046049

Figure S10: Compression Benchmark depicting the compression memory and size for nine compression tools over four datasets represented in FASTQ format. The y-axis is represented as a logarithmic scale of base 2.

1.4.6 Decompression size / memory usage (logarithmic scale)

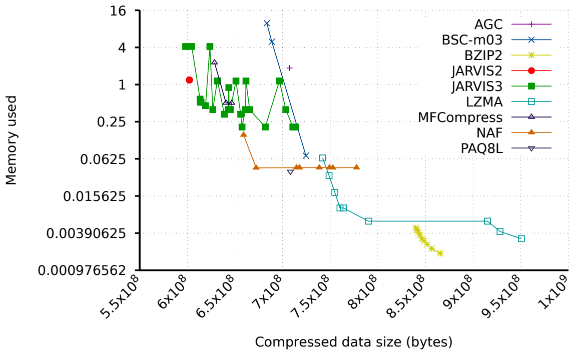

(a) Human T2T genome

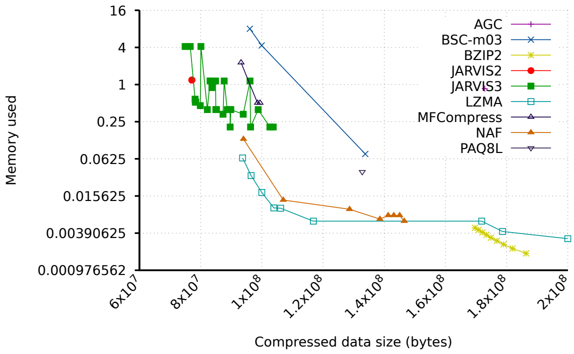

(b) Cassava genome [8]

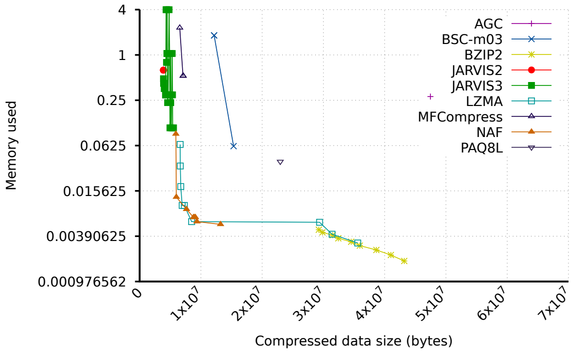

(c) Viral genomes database

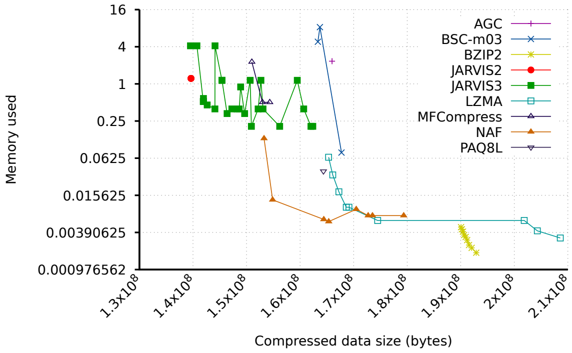

(d) DNA Corpus [9]

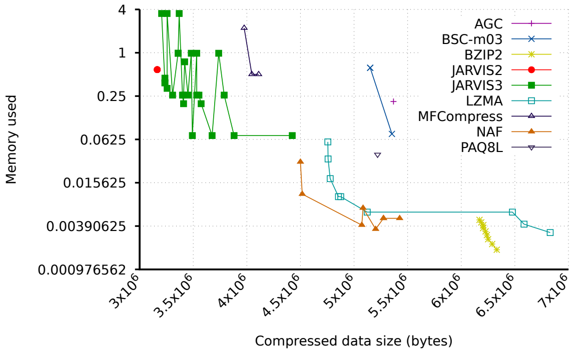

(e) Human Y chromosome (highly-repetitive)

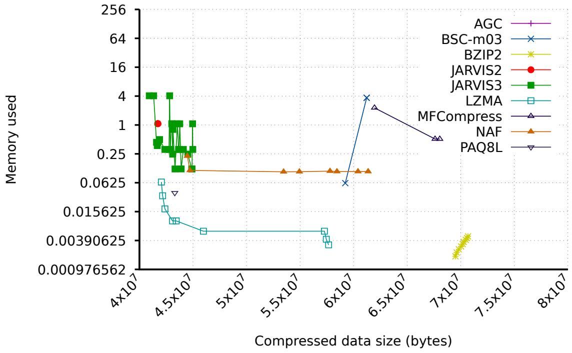

(f) UCSC hg38 7way knownCanonical-exonNuc [10]

Figure S11: Compression Benchmark depicting the decompression memory and size for nine compression tools over six datasets represented in FASTA format. The result for the ACG was not represented in Figure 11f as it represented an outlier. The y-axis is represented as a logarithmic scale of base 2.

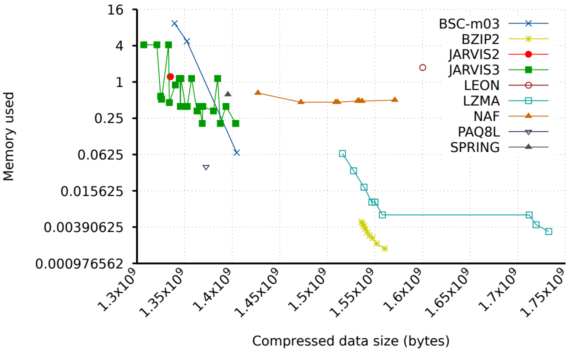

(a) Sample ERR3307082

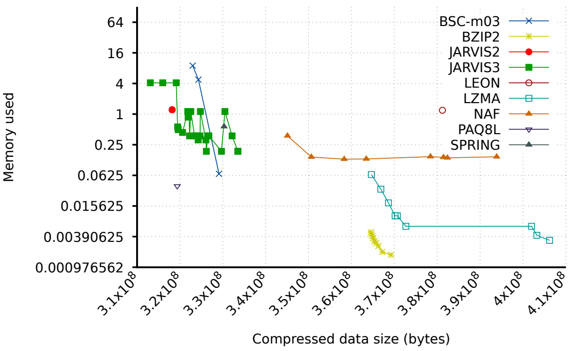

(b) Sample SRR1284073

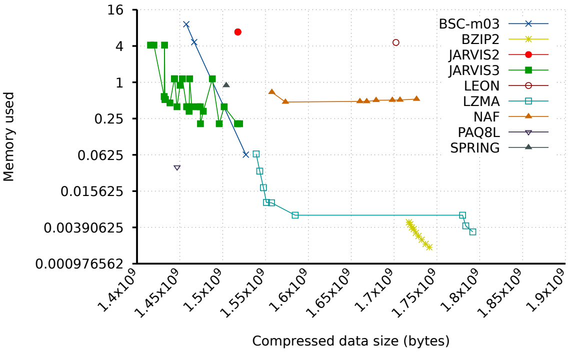

(c) Sample SRR8858470

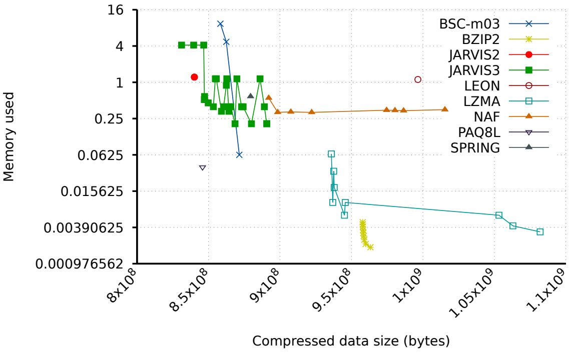

(d) Sample SRR9046049

Figure S12: Compression Benchmark depicting the decompression memory and size for nine compression tools over four datasets represented in FASTQ format. The y-axis is represented as a logarithmic scale of base 2.

2 The JARVIS3 tool

2.1 Installation

JARVIS3 binary can be installed using

```
git clone https://github.com/cobilab/jarvis3
cd jarvis3/src/ && make
```

If the purpose is to compress FASTA or FASTQ data, the following installation is required

```
./JARVIS3.sh --install
```

2.2 Usage

To use JARVIS3 on genomic sequences, the binary (JARVIS3) can be directly used. For using JARVIS3 on FASTA and FASTQ format the JARVIS3.sh (bash) must be used. Below we describe some compression and decompression examples for these three cases.

2.2.1 Genomic sequences

For compressing DNA sequences with JARVIS3, use

```
./JARVIS3 -v -p -l 7 sequence.seq
```

For decompression:

```
./JARVIS3 -d -p -o decompressed.jd sequence.seq.jc
```

This computation will create the decompressed.jd file.

2.2.2 FASTA data

For compressing FASTA data with JARVIS3, use

```
./JARVIS3.sh --fasta --level 7 --block 250MB --threads 4 --input data.fa
```

For decompression:

```
./JARVIS3.sh --decompress --threads 4 --input data.fa.tar
```

2.2.3 FASTQ data

For compressing FASTQ data with JARVIS3, use

```
./JARVIS3.sh --fastq --level 7 --block 250MB --threads 4 --input data.fq
```

For decompression:

```
./JARVIS3.sh --decompress --threads 4 --input data.fq.tar
```

3 Parameters and options of JARVIS3

3.1 Genomic sequences

The command to access the main menu with the options/parameters of JARVIS3 is

```
./JARVIS3 -h
```

or

```
./JARVIS3 --help
```

This command will output the following content

```
NAME
      JARVIS3 v3.6,
      Efficient lossless encoding of genomic sequences

SYNOPSIS
      ./JARVIS3 [OPTION]... [FILE]

SAMPLE
      Run Compression   -> ./JARVIS3 -v -l 13 sequence.txt
```

```
      Run Decompression -> ./JARVIS3 -v -d sequence.txt.jc

DESCRIPTION
      Lossless compression and decompression of genomic
      sequences for minimal storage and analysis purposes.
      Measure an upper bound of the sequence complexity.

      -h,  --help
           Usage guide (help menu).

      -a,  --version
           Display program and version information.

      -x,  --explanation
           Explanation of the context and repeat models.

      -f,  --force
           Force mode. Overwrites old files.

      -v,  --verbose
           Verbose mode (more information).

      -p,  --progress
           Show progress bar.

      -P,  --progress-extended
           Show compression progress for each 5%.

      -d,  --decompress
           Decompression mode.

      -e,  --estimate
           It creates a file with the extension ".iae" with the
           respective information content. If the file is FASTA or
           FASTQ it will only use the "ACGT" (genomic) sequence.

      -s,  --show-levels
           Show pre-computed compression levels (configured).

      -l [NUMBER],  --level [NUMBER]
           Compression level (integer).
           Default level: 7.
           It defines compressibility in balance with computational
           resources (RAM & time). Use -s for levels perception.

      -sd [NUMBER],  --seed [NUMBER]
           Pseudo-random seed.
           Default value: 0.

      -hs [NUMBER],  --hidden-size [NUMBER]
           Hidden size of the neural network (integer).
           Default value: 40.

      -lr [DOUBLE],  --learning-rate [DOUBLE]
           Neural Network leaning rate (double).
           The 0 value turns the Neural Network off.
           Default value: 0.03.

      -o [FILENAME], --output [FILENAME]
           Compressed/decompressed output filename.

      [FILENAME]
           Input sequence filename (to compress) -- MANDATORY.
           File to compress is the last argument.

COPYRIGHT
      Copyright (C) 2014-2024.
      This is a Free software, under GPLv3. You may redistribute
      copies of it under the terms of the GNU - General Public
      License v3 <http://www.gnu.org/licenses/gpl.html>.
```

This menu has a option to access more information about the compression models, namely through the usage of the following command

```
./JARVIS3 -x
```

This option will output the following model help information

```

      -cm [NB_C]:[NB_D]:[NB_I]:[NB_G]/[NB_S]:[NB_E]:[NB_R]:[NB_A]
      Template of a context model.
      Parameters:
      [NB_C]: (integer [1;14]) order size of the regular context
              model. Higher values use more RAM but, usually, are
              related to a better compression score.
      [NB_D]: (integer [1;5000]) denominator to build alpha, which
              is a parameter estimator. Alpha is given by 1/[NB_D].
              Higher values are usually used with higher [NB_C],
              and related to confident bets. When [NB_D] is one,
              the probabilities assume a Laplacian distribution.
      [NB_I]: (integer {0,1,2}) number to define if a sub-program
              which addresses the specific properties of DNA
              sequences (Inverted repeats) is used or not. The
              number 1 turns ON the sub-program using at the same
              time the regular context model. The number 2 does
              only contemple the inversions only (NO regular). The
              number 0 does not contemple its use (Inverted repeats
              OFF). The use of this sub-program increases the
              necessary time to compress but it does not affect the
              RAM.
      [NB_G]: (real [0;1)) real number to define gamma. This value
              represents the decayment forgetting factor of the
              regular context model in definition.
      [NB_S]: (integer [0;20]) maximum number of editions allowed
              to use a substitutional tolerant model with the same
              memory model of the regular context model with
              order size equal to [NB_C]. The value 0 stands for
              turning the tolerant context model off. When the
              model is on, it pauses when the number of editions
              is higher that [NB_C], while it is turned on when
              a complete match of size [NB_C] is seen again. This
              is probabilistic-algorithmic model very useful to
              handle the high substitutional nature of genomic
              sequences. When [NB_S] > 0, the compressor used more
              processing time, but uses the same RAM and, usually,
              achieves a substantial higher compression ratio. The
              impact of this model is usually only noticed for
              higher [NB_C].
      [NB_R]: (integer {0,1}) number to define if a sub-program
              which addresses the specific properties of DNA
              sequences (Inverted repeats) is used or not. It is
              similar to the [NR_I] but for tolerant models.
      [NB_E]: (integer [1;5000]) denominator to build alpha for
              substitutional tolerant context model. It is
              analogous to [NB_D], however to be only used in the
              probabilistic model for computing the statistics of
              the substitutional tolerant context model.
      [NB_A]: (real [0;1)) real number to define gamma. This value
              represents the decayment forgetting factor of the
              substitutional tolerant context model in definition.
              Its definition and use is analogus to [NB_G].

      ... (you may use several context models)


      -rm [NB_R]:[NB_C]:[NB_B]:[NB_L]:[NB_G]:[NB_I]:[NB_W]:[NB_Y]
      Template of a repeat model.
      Parameters:
      [NB_R]: (integer [1;10000] maximum number of repeat models
              for the class. On very repetive sequences the RAM
              increases along with this value, however it also
              improves the compression capability.
      [NB_C]: (integer [1;14]) order size of the repeat context
              model. Higher values use more RAM but, usually, are
              related to a better compression score.
      [NB_B]: (real (0;1]) beta is a real value, which is a
              parameter for discarding or maintaining a certain
              repeat model.
      [NB_L]: (integer (1;20]) a limit threshold to play with
              [NB_B]. It accepts or not a certain repeat model.
      [NB_G]: (real [0;1)) real number to define gamma. This value
              represents the decayment forgetting factor of the
              regular context model in definition.
      [NB_I]: (integer {0,1,2}) number to define if a sub-program
```

```
                which addresses the specific properties of DNA
                sequences (Inverted repeats) is used or not. The
                number 1 turns ON the sub-program using at the same
                time the regular context model. The number 0 does
                not contemple its use (Inverted repeats OFF). The
                number 2 uses exclusively Inverted repeats. The
                use of this sub-program increases the necessary time
                to compress but it does not affect the RAM.
       [NB_W]: (real (0;1)) initial weight for the repeat class.
       [NB_Y]: (integer {0}, [1;50]) maximum cache size. This will
                use a table cache with the specified size. The size
                must be in balance with the k-mer size [NB_C].
```

Nevertheless, several compression level models are already pre-computed. For accessing these pre-levels, the following command must run

```
./JARVIS3 -s
```

This option will output the following levels and model setups

```
Level 1: -rm 1:12:0.90:4:0.72:0:0.1:1
Level 2: -rm 1:12:0.90:4:0.72:1:0.1:1
Level 3: -rm 1:13:0.90:4:0.72:1:0.1:1
Level 4: -rm 1:14:0.90:4:0.72:1:0.1:1
Level 5: -rm 2:12:0.90:5:0.60:1:0.1:1
Level 6: -rm 4:12:0.94:7:0.70:1:0.05:3
Level 7: -rm 3:13:0.90:5:0.72:1:0.1:1
Level 8: -rm 3:14:0.90:5:0.72:1:0.1:1
Level 9: -rm 5:14:0.90:5:0.72:1:0.1:1
Level 10: -rm 6:12:0.90:6:0.78:1:0.03:1
Level 11: -rm 8:13:0.90:6:0.78:1:0.03:2
Level 12: -rm 10:12:0.91:7:0.80:1:0.02:3
Level 13: -rm 12:12:0.90:7:0.81:1:0.02:3
Level 14: -lr 0 -cm 1:1:0:0.9/0:0:0:0 -rm 2:12:0.92:7:0.80:1:0.05:2
Level 15: -lr 0 -cm 3:1:0:0.9/0:0:0:0 -rm 3:12:0.93:7:0.81:1:0.05:3
Level 16: -lr 0 -cm 3:1:0:0.9/0:0:0:0 -rm 4:12:0.92:7:0.81:1:0.03:2
Level 17: -lr 0 -cm 4:1:0:0.9/0:0:0:0 -rm 4:13:0.94:7:0.81:1:0.04:3
Level 18: -lr 0 -cm 6:1:0:0.9/0:0:0:0 -rm 4:13:0.94:7:0.81:1:0.04:3
Level 19: -lr 0 -cm 6:1:0:0.9/0:0:0:0 -rm 8:12:0.93:7:0.81:1:0.02:3
Level 20: -lr 0 -cm 4:1:0:0.9/0:0:0:0 -rm 20:12:0.9:7:0.85:1:0.01:4
Level 21: -lr 0 -cm 4:1:0:0.9/0:0:0:0 -rm 50:12:0.9:7:0.85:1:0.01:5
Level 22: -lr 0 -cm 4:1:0:0.9/0:0:0:0 -rm 100:12:0.9:7:0.85:1:0.01:5
Level 23: -lr 0 -cm 4:1:0:0.9/0:0:0:0 -rm 200:12:0.9:7:0.85:1:0.01:6
Level 24: -lr 0 -cm 6:1:0:0.9/0:0:0:0 -rm 6:15:0.93:6:0.81:1:0.02:1
Level 25: -lr 0.03 -hs 24 -cm 6:1:0:0.9/0:0:0:0 -rm 6:15:0.92:6:0.81:1:0.02:1
Level 26: -lr 0.03 -hs 32 -cm 4:1:0:0.9/0:0:0:0 -rm 20:15:0.90:7:0.82:1:0.02:1
Level 27: -lr 0.03 -hs 24 -cm 6:1:0:0.9/0:0:0:0 -rm 15:13:0.92:7:0.85:0:0.02:4 -rm
    13:12:0.92:7:0.84:2:0.01:3
Level 28: -lr 0.03 -hs 42 -cm 6:1:0:0.9/0:0:0:0 -rm 6:15:0.93:6:0.81:1:0.02:1
Level 29: -lr 0.03 -hs 42 -cm 6:1:0:0.9/0:0:0:0 -rm 10:15:0.93:6:0.81:1:0.02:1
Level 30: -lr 0.03 -hs 42 -cm 6:1:0:0.9/0:0:0:0 -rm 10:15:0.93:6:0.81:0:0.02:1 -rm
    10:15:0.93:6:0.81:2:0.02:1
Level 31: -lr 0.03 -hs 48 -cm 1:1:0:0.9/0:0:0:0 -cm 4:1:0:0.9/0:0:0:0 -cm 8:1:1:0.89/0:0:0:0 -
    cm 12:20:1:0.97/0:0:0:0 -rm 300:12:0.9:7:0.85:0:0.01:10 -rm 200:12:0.9:7:0.8:2:0.01:4
Level 32: -lr 0.04 -hs 64 -cm 1:1:0:0.9/0:0:0:0 -cm 4:1:0:0.9/0:0:0:0 -cm 8:1:1:0.89/0:0:0:0 -
    cm 12:20:1:0.97/0:0:0:0 -rm 500:12:0.9:7:0.85:0:0.01:12 -rm 200:12:0.9:7:0.8:2:0.01:4
Level 33: -lr 0.04 -hs 86 -cm 1:1:0:0.9/0:0:0:0 -cm 4:1:0:0.9/0:0:0:0 -cm 8:1:1:0.89/0:0:0:0 -
    cm 12:20:1:0.97/0:0:0:0 -rm 500:12:0.9:7:0.85:0:0.01:12 -rm 200:12:0.9:7:0.8:2:0.01:4
Level 34: -lr 0.04 -hs 256 -cm 1:1:0:0.9/0:0:0:0 -cm 4:1:0:0.9/0:0:0:0 -cm 8:1:1:0.9/0:0:0:0 -
    cm 12:20:1:0.97/0:0:0:0 -rm 1500:12:0.9:7:0.85:0:0.01:10 -rm 500:12:0.9:7:0.82:2:0.01:3
Level 35: -lr 0.04 -hs 248 -cm 1:1:0:0.9/0:0:0:0 -cm 3:1:0:0.9/0:0:0:0 -cm 7:1:0:0.9/0:0:0:0 -
    cm 9:1:1:0.9/0:0:0:0 -cm 11:10:0:0.9/0:0:0:0 -rm 100:14:0.9:7:0.85:1:0.01:3 -rm
    200:12:0.88:7:0.85:0:0.01:3 -rm 300:12:0.87:7:0.85:2:0.01:3
Level 36: -lr 0.04 -hs 248 -cm 1:1:0:0.9/0:0:0:0 -cm 3:1:0:0.9/0:0:0:0 -cm 7:1:0:0.9/0:0:0:0 -
    cm 9:1:1:0.9/0:0:0:0 -cm 11:10:0:0.9/0:0:0:0 -cm 13:200:1:0.9/1:10:1:0.9 -rm
    100:14:0.9:7:0.85:1:0.01:3 -rm 200:12:0.88:7:0.85:0:0.01:8 -rm 300:12:0.87:7:0.85:2:0.01:3
Level 37: -lr 0.01 -hs 248 -cm 1:1:0:0.9/0:0:0:0 -cm 3:1:0:0.9/0:0:0:0 -cm 6:1:0:0.9/0:0:0:0 -
    cm 9:1:0:0.9/0:0:0:0 -cm 11:10:1:0.9/0:0:0:0 -cm 14:200:1:0.9/1:10:1:0.9 -rm
    300:14:0.88:7:0.85:0:0.01:8 -rm 300:14:0.88:7:0.85:2:0.01:8 -rm
    500:12:0.88:7:0.85:0:0.01:15
Level 38: -lr 0 -cm 12:1:0:0.7/0:0:0:0 -rm 2:14:0.95:1:0.9:1:0.1:1
Level 39: -lr 0 -cm 12:1:0:0.7/0:0:0:0 -rm 3:14:0.95:1:0.9:1:0.1:1
Level 40: -lr 0.03 -lr 32 -cm 12:1:0:0.7/0:0:0:0 -rm 4:14:0.95:1:0.9:1:0.1:1
```

3.2 FASTA and FASTQ data

The command to access the main menu with the options/parameters of JARVIS3.sh is

```
./JARVIS3.sh -h
```

or

```
./JARVIS3.sh --help
```

This command will output the following content

```
----------------------------------------------------

 JARVIS3, v3.6. High reference-free compression of DNA
               sequences, FASTA data, and FASTQ data.

 Program options -------------------------------------

 -h, --help                    Show this,
 -a, --about                   Show program information,
 -c, --install                 Install/compile programs,
 -s, --show                    Show compression levels,

 -l <INT>, --level <INT>       JARVIS3 compression level,
 -b <INT>, --block <INT>       Block size to be splitted,
 -t <INT>, --threads <INT>     Number of JARVIS3 threads,

 -dn, --dna                    Assume DNA sequence type,
 -fa, --fasta                  Assume FASTA data type,
 -fq, --fastq                  Assume FASTQ data type,
 -au, --automatic              Detect data type (def),

 -d, --decompress              Decompression mode,

 Input options ---------------------------------------

 -i <FILE>, --input <FILE>     Input DNA filename.

 Example ---------------------------------------------

 ./JARVIS3.sh --block 16MB -t 8 -i sample.seq
 ./JARVIS3.sh --decompress -t 4 -i sample.seq.tar

 ----------------------------------------------------
```

4 Reproducibility

4.1 Reproducing the Benchmark

In the benchmark/ folder of the JARVIS3 repository, please type

```
bash ./RunAll.sh
```

The output is provided in the same folder, including the plots with the respective benchmark for each dataset.

5 Computing environment

In our study, a computer running Linux Ubuntu 22 LTS equipped with 8 Intel® Core™ i7-6700 CPU cores operating at 3.40GHz and 64 GB of RAM was used to execute the benchmark.

References

[1] A J Pinho, D Pratas, and P JSG Ferreira. Bacteria DNA sequence compression using a mixture of finite-context models. In *2011 IEEE Statistical Signal Processing Workshop (SSP)*, pages 125–128. IEEE, 2011.

[2] M Silva, D Pratas, and A J Pinho. Efficient DNA sequence compression with neural networks. *GigaScience*, 9(11):giaa119, 2020.

[3] D Pratas and A J Pinho. JARVIS2: a data compressor for large genome sequences. In *2023 Data Compression Conference (DCC)*, pages 288–297. IEEE, 2023.

[4] M Hiransha, E A Gopalakrishnan, V K Menon, and K P Soman. NSE stock market prediction using deep-learning models. *Procedia Computer Science*, 132:1351–1362, 2018.

[5] M Silva, D Pratas, and A J Pinho. AC2: An Efficient Protein Sequence Compression Tool Using Artificial Neural Networks and Cache-Hash Models. *Entropy*, 23(5):530, 2021.

[6] A J Pinho and D Pratas. Copy models for protein sequence compression. In *2024 Data Compression Conference (DCC)*. IEEE, 2024.

[7] H Robbins and S Monro. A stochastic approximation method. *The Annals of Mathematical Statistics*, pages 400–407, 1951.

[8] Weihong Qi, Yi-Wen Lim, Andrea Patrignani, Pascal Schläpfer, Anna Bratus-Neuenschwander, Simon Grüter, Christelle Chanez, Nathalie Rodde, Elisa Prat, Sonia Vautrin, et al. The haplotype-resolved chromosome pairs of a heterozygous diploid African cassava cultivar reveal novel pan-genome and allele-specific transcriptome features. *GigaScience*, 11:giac028, 2022.

[9] Diogo Pratas and Armando J Pinho. A DNA sequence corpus for compression benchmark. In *Practical Applications of Computational Biology and Bioinformatics, 12th International Conference*, pages 208–215. Springer, 2019.

[10] W James Kent, Charles W Sugnet, Terrence S Furey, Krishna M Roskin, Tom H Pringle, Alan M Zahler, and David Haussler. The human genome browser at UCSC. *Genome research*, 12(6):996–1006, 2002.
